# An accurate and efficient framework for modelling the surface chemistry of ionic materials

**DOI:** 10.1038/s41557-025-01884-y

**Published:** 2025-08-13

**Authors:** Benjamin X. Shi, Andrew S. Rosen, Tobias Schäfer, Andreas Grüneis, Venkat Kapil, Andrea Zen, Angelos Michaelides

**Affiliations:** 1 Yusuf Hamied Department of Chemistry, University of Cambridge, Cambridge, United Kingdom; 2 Initiative for Computational Catalysis, Flatiron Institute, New York, NY USA; 3 Department of Chemical and Biological Engineering, Princeton University, Princeton, NJ USA; 4 Institute for Theoretical Physics, TU Wien, Vienna, Austria; 5 Department of Physics and Astronomy, University College London, London, UK; 6 Thomas Young Centre and London Centre for Nanotechnology, London, UK; 7 Dipartimento di Fisica Ettore Pancini, Università di Napoli Federico II, Napoli, Italy; 8 Department of Earth Sciences, University College London, London, United Kingdom

**Keywords:** Quantum chemistry, Surface chemistry, Computational chemistry

## Abstract

Quantum-mechanical simulations can offer atomic-level insights into chemical processes on surfaces that are crucial to advancing applications in heterogeneous catalysis, energy storage and greenhouse gas sequestration. Unfortunately, achieving the accuracy needed for reliable predictions has proven challenging. Density functional theory, widely used for its efficiency, can be inconsistent, necessitating accurate methods from correlated wavefunction theory. But high computational demands and substantial user intervention have traditionally made correlated wavefunction theory impractical to carry out for surfaces. Here we present an automated framework that leverages multilevel embedding approaches to apply correlated wavefunction theory to the surfaces of ionic materials with computational costs approaching those of density functional theory. With this framework, we reproduce experimental adsorption enthalpies for a diverse set of 19 adsorbate–surface systems. We further resolve debates on the adsorption configuration of several systems, while offering benchmarks to assess density functional theory. This framework is open source, facilitating the routine application of correlated wavefunction theory to complex problems involving the surfaces of ionic materials.

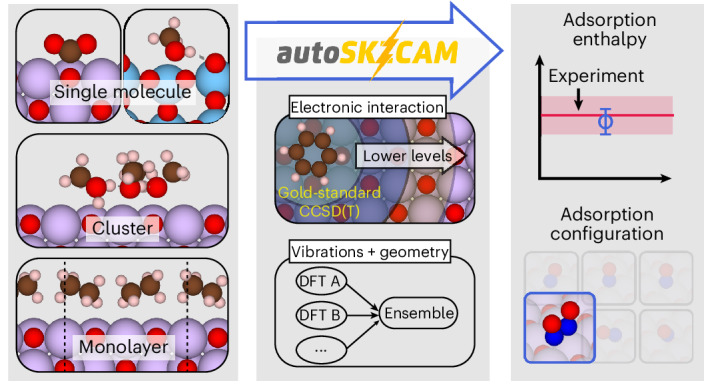

## Abstract (web summary)

Simulating molecular adsorption on surfaces presents considerable challenges, as computational methods typically suffer from either insufficient accuracy or prohibitive computational costs. Now, with an open-source multilevel embedding approach, adsorption processes on the surfaces of ionic materials can be modelled routinely with an accuracy comparable to that of experiments.

## Main

Understanding the chemical processes occurring on surfaces is critical to applications ranging from the production of fuels via heterogeneous catalysis^1^ to the storage of energy and sequestration of greenhouse gases. The adsorption and desorption of molecules from surfaces is a crucial process within all of these applications, and the strength of this binding is dictated by the adsorption enthalpy *H*_ads_, making it a fundamental quantity to accurately predict. For example, candidate materials for CO_2_ or H_2_ gas storage are screened based on their *H*_ads_ value, often to within tight energetic windows^2^ (~150 meV). High accuracy of *H*_ads_ is also needed to compare the competitive adsorption between two molecular species for the separation of flue gases^3^. Finally, *H*_ads_ is a necessary quantity within any (microkinetic) model of a surface chemical reaction, with an empirical dependence between the reaction rate and *H*_ads_ according to well-established volcano plots^4^.

The rational design of new materials for the aforementioned applications relies on an atomic-level understanding of surface processes, together with an accurate *H*_ads_. Determining the adsorption configuration—the geometry a molecule adopts on a surface—is particularly important, as it underpins all subsequent processes. Quantum-mechanical simulation techniques can provide the atomic-level detail needed to study the adsorption configuration. They have become widely used to complement experimental techniques, where such detail is typically hard to obtain. But achieving reliable agreement between theory and experiments in determining *H*_ads_ is challenging due to limitations and inaccuracies in the theoretical methods that are commonly employed and the frequent neglect of thermal contributions. Moreover, these inaccuracies can affect the predicted adsorption configuration, leading to incorrect identification of the most stable configuration, or a fortuitous match to experimental *H*_ads_ for a metastable configuration.

**Fig. 1:**
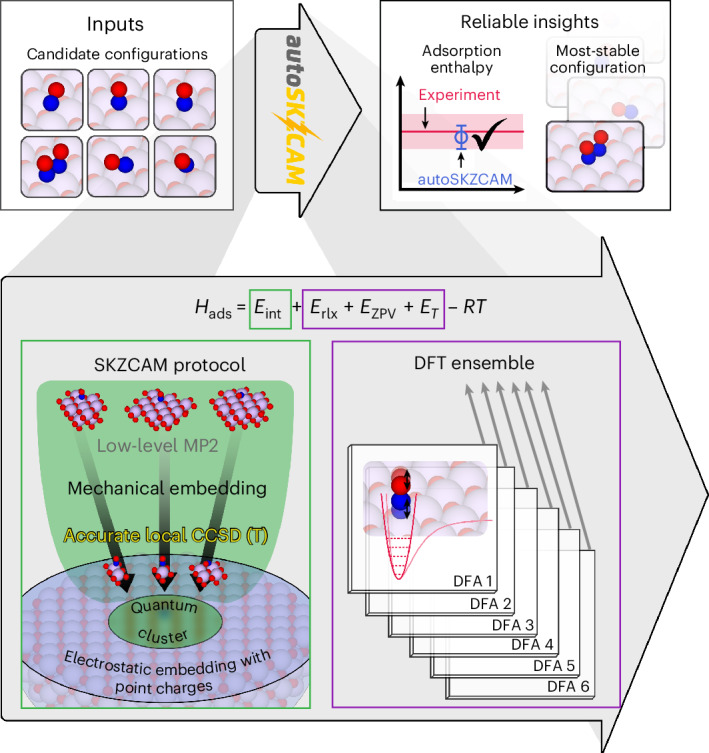
Reliable insights into the surface chemistry of ionic materials with the autoSKZCAM framework. Schematic description of the open-source autoSKZCAM framework. From a set of adsorbate–surface configurations, this framework can identify the most stable configuration and calculate an adsorption enthalpy *H*_ads_ that reproduces experiment. It partitions *H*_ads_ via a divide-and-conquer scheme. The dominant contribution—the interaction energy *E*_int_—is treated up to the gold-standard level of CCSD(T) through the SKZCAM protocol. This protocol ensures a low cost on *E*_int_ by employing a multilevel approach, where CCSD(T) with a local approximation is embedded within more affordable levels of theory such as second-order Møller–Plesset perturbation theory (MP2). The remaining relaxation energy *E*_rlx_, zero-point vibrational energy *E*_ZPV_ and thermal contributions *E*_T_ are treated through an ensemble of six widely used DFAs in DFT, enabling an (averaged) estimate with a corresponding error prediction. Within the *RT* contribution, *R* refers to the ideal gas constant, while *T* is the temperature.

To address these challenges, new techniques are needed that surpass the traditional cost–accuracy trade-off; they must achieve high accuracy of *H*_ads_ (rivalling that of experiments) while being fast enough to sample multiple adsorption sites and configurations to correctly identify the most stable configuration. Density functional theory (DFT) is the current workhorse technique, playing an important role in identifying the reactivity trends (for example, Brønsted–Evans–Polanyi relationships^4^^,^^5^ and volcano plots^4^) that now form pivotal tools for the in silico design of new solid catalysts^1^. Despite these successes, the density functional approximations (DFAs) to the exchange–correlation functional and dispersion interactions within DFT are not systematically improvable, presenting ongoing challenges in making reliable predictions. For example, six different adsorption configurations have been proposed by different DFT studies for NO adsorbed on the MgO(001) surface (Fig. 1).

Correlated wavefunction theory (cWFT) provides a broadly systematically improvable hierarchy of methods, where coupled cluster theory with single, double and perturbative triple excitations (CCSD(T)) is widely considered the method of choice. Its high cost and steep computational scaling, however, limit its direct application to adsorbate–surface systems. To lower this cost, the surface is approximated as a finite cluster placed within an appropriate embedding environment^6–8^. For ionic materials, this embedding environment typically consists of point charges to represent the long-range interactions from the rest of the surface. While this approach has demonstrated great success, it remains costly, and considerable (technical) complexity exists with applying it. Consequently, studies until now have been mostly limited to one or two systems; it is challenging to tackle the broad sets of adsorbate–surface systems or adsorption configurations commonly performed within DFT studies. To facilitate routine application of cWFT to surface problems, the methods need to be streamlined, automatized and simplified into black-box tools, providing reliable insights from simple inputs (as illustrated in the top panel of Fig. 1).

**Fig. 2:**
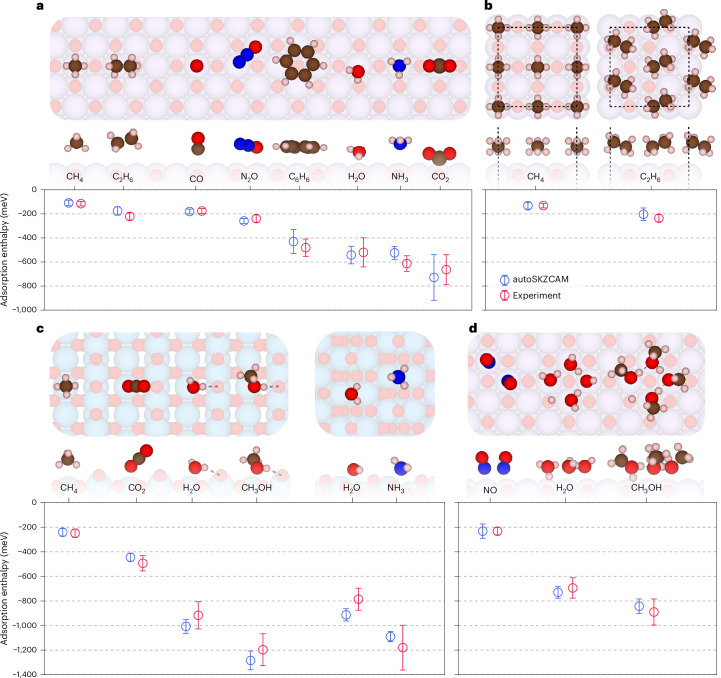
Consensus with experiments for a range of adsorbates on ionic surfaces. **a**–**d**, Comparison of adsorption enthalpies computed with the autoSKZCAM framework with those of high-quality experiments for a set of 19 adsorbate–surface combinations. These include single molecules adsorbed on the MgO(001) surface (**a**), monolayers adsorbed on MgO(001) (**b**) and single molecules adsorbed on rutile TiO_2_(110) and anatase TiO_2_(101) (**c**), as well as clusters adsorbed on MgO(001) (**d**). Experimental errors of *H*_ads_ values for most systems are based on temperature-programmed desorption (TPD) analysis by Campbell and Sellers^23^, taken as twice the standard deviation in the predicted pre-exponential factor against a test set of ~20 adsorbed molecules (Supplementary Section 11). Simulation errors are the root squared sum of several systematic contributions, described in Supplementary Section 9, with the majority arising from errors using a geometry optimized by DFT, which we estimate as twice the root mean square error from an ensemble of six DFAs. A top view and side view of the most stable geometry for each system are shown above each label, with C, H, N, O, Mg and Ti atoms corresponding to the brown, white, dark blue, red, purple and light blue spheres, respectively.

In this work we introduce the open-source autoSKZCAM framework, which delivers CCSD(T)-quality predictions to surface chemistry problems involving ionic materials at a cost and ease approaching that of DFT. As summarized in Fig. 1, these qualities are achieved by partitioning *H*_ads_ into separate contributions (discussed in the Methods and Supplementary Section 3) that are addressed with appropriate, accurate techniques within a divide-and-conquer scheme. We assess the performance of this framework at predicting *H*_ads_ for a set of 19 adsorbate–surface systems (visualized in Fig. 2 and Supplementary Section 4), including a diverse array of molecules adsorbed on MgO(001) as well as anatase TiO_2_(101) and rutile TiO_2_(110). We further leverage its low cost to study multiple adsorption configurations for some of the adsorbate–surface systems with the aim to resolve prior debates between experiments and simulations on the most stable adsorption configuration. Finally, we showcase its utility as a source of benchmarks for assessing the performance of DFAs in DFT to facilitate future advances in DFA development.

## Results

### Agreement across diverse systems

The 19 adsorbate–surface systems studied within this work are shown in Fig. 2, where the *H*_ads_ computed by the autoSKZCAM framework is evaluated against experiment. In all of the systems, the autoSKZCAM framework was able to reproduce experimental *H*_ads_ measurements (tabulated and visualized in greater detail in Supplementary Section 9) within their respective errors bars (discussed in the Methods). These systems have a range of *H*_ads_ values which cover almost 1.5 eV, spanning weak physisorption to strong chemisorption and including a diverse set of molecules (CO, NO, N_2_O, NH_3_, H_2_O, CO_2_, CH_3_OH, CH_4_, C_2_H_6_ and C_6_H_6_) on common surfaces of ionic materials (MgO(001) as well as anatase and rutile TiO_2_). Besides the adsorption of small single molecules, some of which have been tackled before, this work also studies monolayers (Fig. 2b) and larger molecules such as C_6_H_6_ or molecular clusters of CH_3_OH and H_2_O (Fig. 2d).

**Extended Data Fig. 1:**
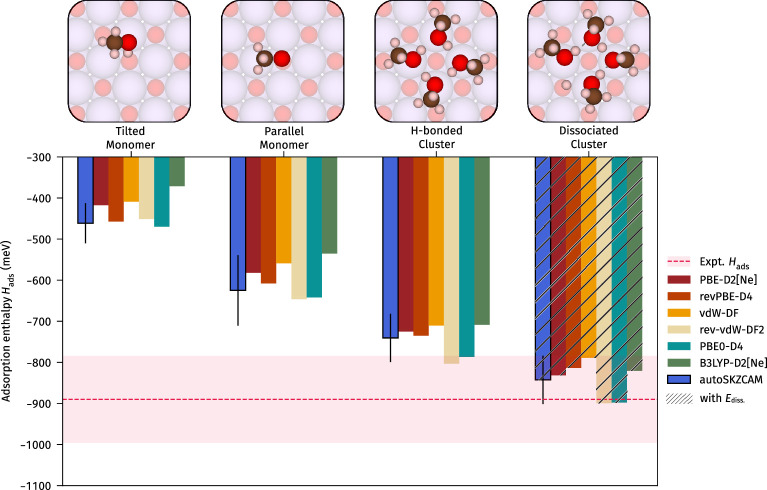
Stabilisation of CH_3_OH clusters on MgO(001) through H-bonding and dissociation. Adsorption enthalpy *H*_ads_ (per molecule) calculated with the autoSKZCAM framework and 6 different density functional approximations for CH_3_OH on MgO(001). These are compared against TPD measurements by Günster et al.^69^. We consider the tilted and parallel adsorption configuration of a single CH_3_OH molecule on MgO(001) as well as a molecularly adsorbed and dissociated tetramer. The *H*_ads_ of the latter was computed by adding an additional term, *E*_diss_, onto the molecularly adsorbed tetramer, as discussed in Supplementary Section 8E. The experimental error on *H*_ads_ for this system is based on temperature programmed desorption analysis by Campbell and Sellers^23^, taken as twice the standard deviation in the predicted pre-exponential factor against a test set of ~ 20 adsorbed molecules (Supplementary Section 11). The autoSKZCAM simulation errors are the root squared sum of several contributions described in Supplementary Section 9, with the majority arising from errors using a geometry optimised by density functional theory, which we estimate as twice the root-mean-squared-error from an ensemble of 6 density functional approximations.

**Extended Data Fig. 2:**
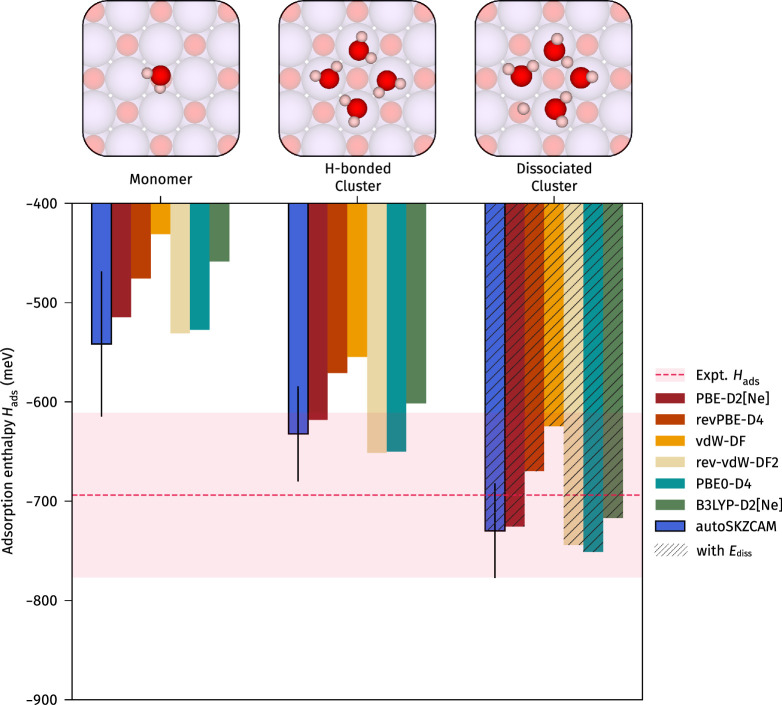
Stabilisation of H_2_O clusters on MgO(001) through H-bonding and dissociation. Adsorption enthalpy *H*_ads_ (per molecule) calculated with the autoSKZCAM framework and 6 different density functional approximations for H_2_O on MgO(001). These are compared against TPD measurements by Stirniman et al.^70^. We consider the adsorption of a single molecule, as well as a molecularly adsorbed and dissociated tetramer. The *H*_ads_ of the latter was computed by adding an additional term, *E*_diss_, onto the molecularly adsorbed tetramer, as discussed in Supplementary Section 8E. The experimental error on *H*_ads_ for this system is based on temperature programmed desorption analysis by Campbell and Sellers^23^, taken as twice the standard deviation in the predicted pre-exponential factor against a test set of ~ 20 adsorbed molecules (Supplementary Section 11). The autoSKZCAM simulation errors are the root squared sum of several contributions described in Supplementary Section 9, with the majority arising from errors using a geometry optimised by density functional theory, which we estimate as twice the root-mean-squared-error from an ensemble of 6 density functional approximations.

The ability to study large systems, including molecular clusters on the surface, with the autoSKZCAM framework has been crucial towards reproducing experiments. For example, we have studied several adsorption configurations of CH_3_OH on MgO(001), including hydrogen-bonded and partially dissociated clusters of CH_3_OH. We find that agreement with experiment in Fig. 2 can be obtained only when considering partially dissociated clusters. As discussed in Supplementary Section 1a and Extended Data Fig. 1, other studied structures predict less stable adsorbates (that is, a weaker absolute *H*_ads_). We show in Extended Data Fig. 2 that these insights are transferable to H_2_O, which also forms partially dissociated clusters on MgO(001).

### Reliable insights at the atomic level

The automated nature and affordable cost of the autoSKZCAM framework allows us to compare the *H*_ads_ values across several configurations, which the adsorbate can adopt in each adsorbate–surface system. Beyond H_2_O and CH_3_OH, we used this framework to identify the most stable adsorption configuration of N_2_O, CO_2_ and NO on MgO(001) as well as CO_2_ on rutile TiO_2_(110)—systems with debate within the literature. Here, inaccuracies in the DFAs within DFT can lead to ambiguities when determining the stable adsorption configuration through two possible paths: (1) the DFA predicts the wrong stable adsorption configuration or (2) a metastable adsorption configuration fortuitously matches the experimental *H*_ads_. The autoSKZCAM framework ensures that the experimental *H*_ads_ is reproduced only when the correct stable adsorption configuration (corresponding to the most negative *H*_ads_) has been identified.

**Fig. 3:**
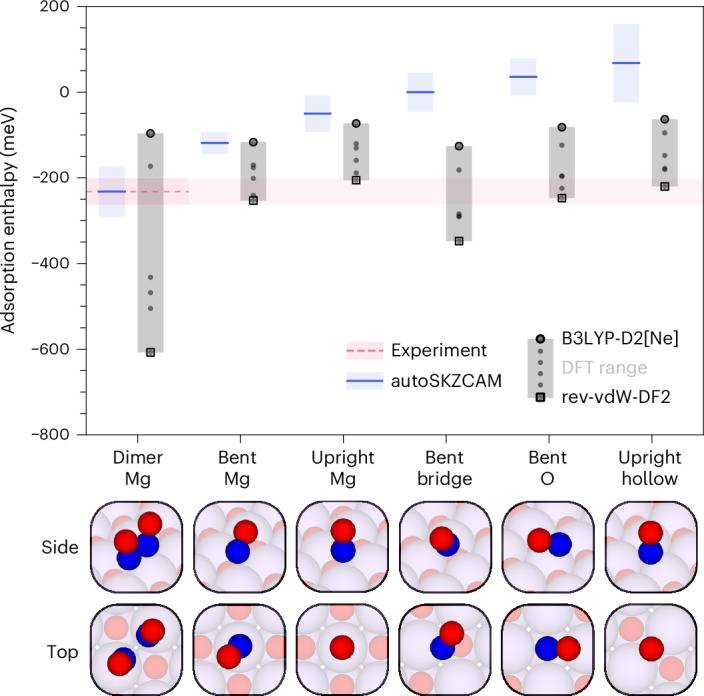
Correct identification of the NO on MgO(001) adsorption configuration. For NO on MgO(001), six adsorption configurations have been proposed: ‘dimer Mg’, ‘bent Mg’, ‘upright Mg’, ‘bent bridge’, ‘bent O’ and ‘upright hollow’. These names reflect the orientation and binding site on the surface. The adsorption enthalpy *H*_ads_ is calculated for each configuration with the autoSKZCAM framework and a set of six DFAs in DFT. The estimates from the six DFAs are plotted as grey-filled circles, with a light grey bar to highlight the range between the smallest and largest values. Experiments^10,11^ indicate that the dimer Mg configuration is the most stable. The autoSKZCAM framework, plotted as blue lines with a corresponding error bar, is the only method that correctly identifies this configuration while reproducing the experimental *H*_ads_ measurement by Wichtendahl et al.^43^. The experimental error on *H*_ads_ is based on temperature-programmed desorption (TPD) analysis by Campbell and Sellers^23^, taken as twice the standard deviation in the predicted pre-exponential factor against a test set of ~20 adsorbed molecules (Supplementary Section 11). The experimental error range is indicated with a light pink bar, which is highlighted with a dashed pink line for the lowest energy ‘dimer Mg’ configuration. The autoSKZCAM simulation errors are the root squared sum of several systematic contributions described in Supplementary Section 9, with the majority arising from errors using a geometry optimized by DFT, which we estimate as twice the root mean square error from an ensemble of six DFAs. The DFAs used are PBE-D2[Ne], revPBE-D4, vdW-DF2, rev-vdW-DF2, PBE0-D4 and B3LYP-D2[Ne], with B3LYP-D2[Ne] and rev-vdW-DF2 explicitly indicated with an open circle and square, respectively, as these sit at either end of the DFT range. The autoSKZCAM and DFT estimates are tabulated in Supplementary Section 1c.

The ambiguities from using DFT are particularly evident in the adsorption of NO on MgO(001), where different DFAs (and procedures) have led to the identification of multiple ‘stable’ geometries in six broad classes. In Fig. 3, we present *H*_ads_ estimates by several widely used DFAs for these six adsorption configurations. For all six configurations, DFAs exist that yield *H*_ads_ values that agree with experiment. Notably, the rev-vdW-DF2 DFA^9^ predicts *H*_ads_ values that agree with experiments for the ‘bent Mg’, ‘upright Mg’, ‘bent O’ and ‘upright hollow’ adsorption configurations. On the basis of such fortuitous agreement, prior studies (Supplementary Section 13) have misidentified several of these configurations as being the most stable. The autoSKZCAM framework identifies the (covalently bonded) dimer *cis*-(NO)_2_ configuration (dubbed the ‘dimer Mg’ configuration) to be the most stable, with an *H*_ads_ consistent with experiment, while all other (monomer) configurations are predicted to be less stable by more than 80 meV. This prediction is commensurate with findings from Fourier-transform infrared spectroscopy^10^ and electron paramagnetic resonance^11^ experiments, both of which suggest that NO exists as a dimer on MgO(001), aside from a small number of monomers adsorbed on defect sites.

**Extended Data Fig. 3:**
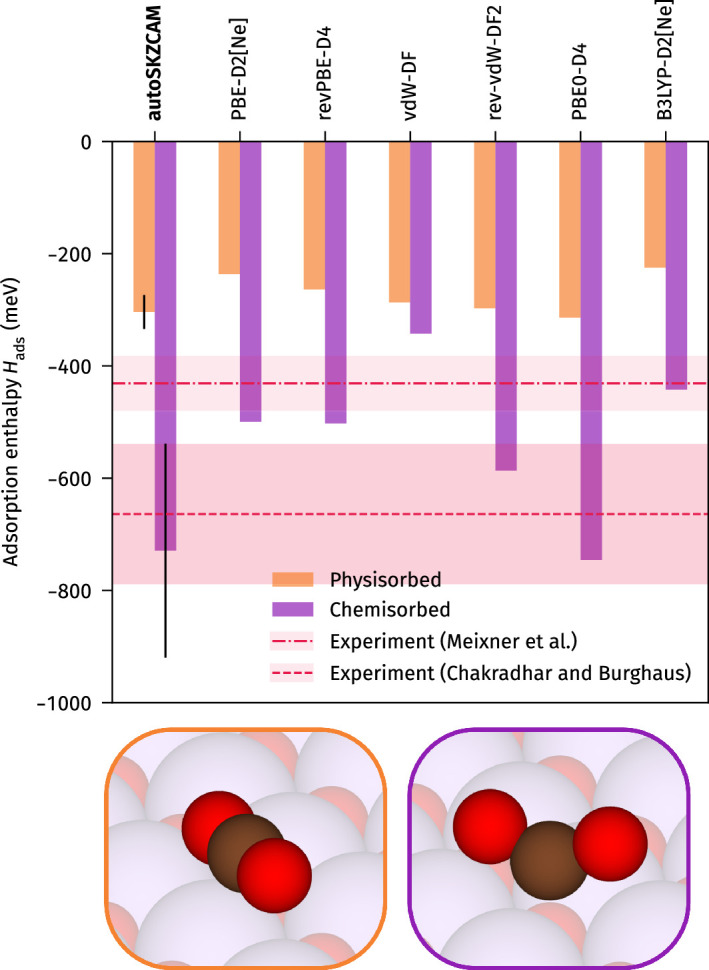
Resolving the chemisorbed state of CO_2_ on MgO(001). Adsorption enthalpy *H*_ads_ calculated with the autoSKZCAM framework and 6 different density functional approximations for the physisorbed (orange) and chemisorbed (purple) state of CO_2_ on MgO(001). These are compared against TPD measurements by Meixner et al.^13^ and Chakradhar and Burghaus^14^, which propose a physisorbed and chemisorbed state, respectively. The experimental errors on *H*_ads_ for this system is based on temperature programmed desorption analysis by Campbell and Sellers^23^, taken as twice the standard deviation in the predicted pre-exponential factor against a test set of ~ 20 adsorbed molecules (Supplementary Section 11). The autoSKZCAM simulation errors are the root squared sum of several contributions described in Supplementary Section 9, with the majority arising from errors using a geometry optimised by density functional theory, which we estimate as twice the root-mean-squared-error from an ensemble of 6 density functional approximations.

**Extended Data Fig. 4:**
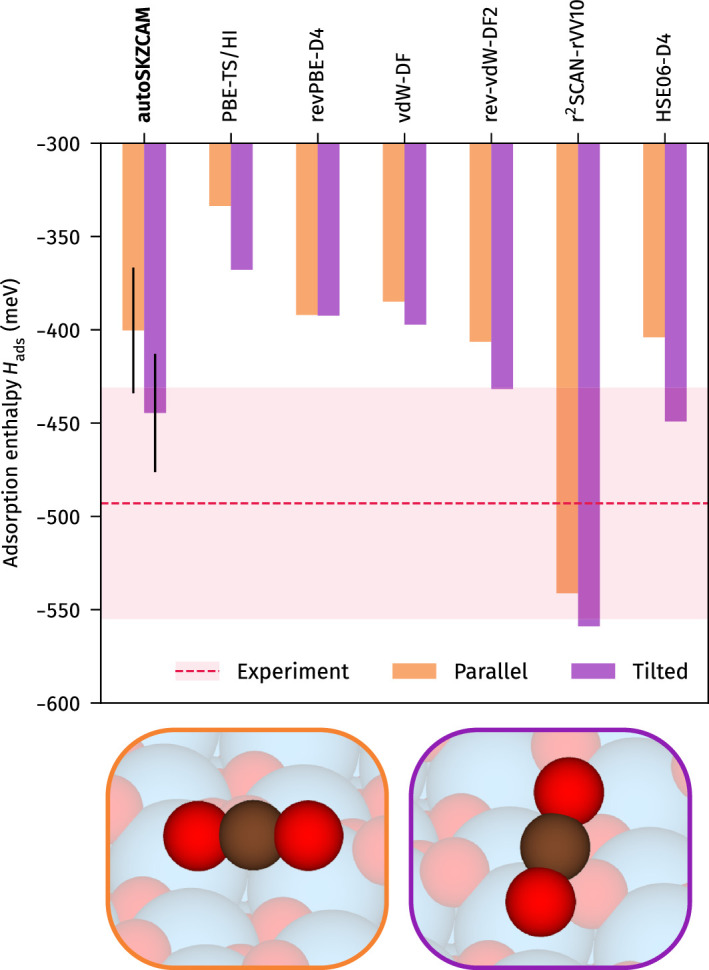
Preference towards the tilted configuration of CO_2_ on the TiO_2_ rutile(110). Adsorption enthalpy *H*_ads_ calculated with the autoSKZCAM framework and 6 different density functional approximations for the parallel (orange) and tilted (purple) adsorption configuration of CO_2_ on TiO_2_ rutile(110). These are compared against TPD measurements by Thompson et al.^71^. The experimental error on *H*_ads_ for this system is based on temperature programmed desorption analysis by Campbell and Sellers^23^, taken as twice the standard deviation in the predicted pre-exponential factor against a test set of ~ 20 adsorbed molecules (Supplementary Section 11). The autoSKZCAM simulation errors are the root squared sum of several contributions described in Supplementary Section 9, with the majority arising from errors using a geometry optimised by density functional theory, which we estimate as twice the root-mean-squared-error from an ensemble of 6 density functional approximations.

**Extended Data Fig. 5:**
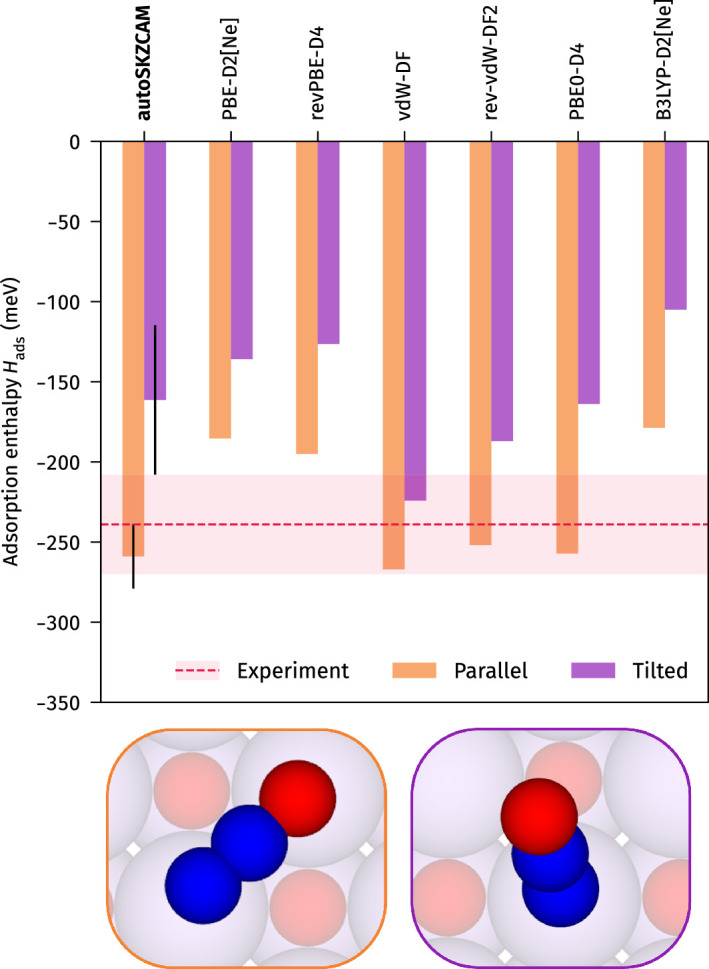
N_2_O on MgO(001) adopts a parallel configuration. Adsorption enthalpy *H*_ads_ calculated with the autoSKZCAM framework and 6 different density functional approximations for the parallel (orange) and tilted (purple) adsorption configuration of N_2_O on MgO(001). These are compared against TPD measurements by Lian et al.^72^. The experimental error on *H*_ads_ for this system is based on temperature programmed desorption analysis by Campbell and Sellers^23^, taken as twice the standard deviation in the predicted pre-exponential factor against a test set of ~ 20 adsorbed molecules (Supplementary Section 11). The autoSKZCAM simulation errors are the root squared sum of several contributions described in Supplementary Section 9, with the majority arising from errors using a geometry optimised by density functional theory, which we estimate as twice the root-mean-squared-error from an ensemble of 6 density functional approximations.

In many cases, debates on the most stable adsorption configuration cannot be resolved from experiments alone. For example, techniques like Fourier-transform infrared spectroscopy, low-energy electron diffraction, or X-ray and ultraviolet photoelectron spectroscopies provide only indirect evidence. Moreover, while scanning tunnelling microscopy offers real-space images, its resolution is often insufficient for definitive interpretation^12^. The autoSKZCAM framework can be valuable within such contexts. Notably, both experiments^13,14^ and simulations^15–18^ have debated between a chemisorbed or physisorbed configuration (Extended Data Fig. 3 and Supplementary Section 1b) of CO_2_ on MgO(001). With the autoSKZCAM framework, we show that it takes on a chemisorbed carbonate configuration, in agreement with previous temperature-programmed desorption (TPD) measurements^14,19^. Similarly, the adsorption of CO_2_ (refs. ^7,20^) on rutile TiO_2_(110) (Extended Data Fig. 4 and Supplementary Section 1d) and N_2_O (ref. ^21^) on MgO(001) (Extended Data Fig. 5 and Supplementary Section 1e) have been debated to take on either a tilted or parallel geometry; the autoSKZCAM framework predicts the tilted geometry to be the most stable for the former and the parallel geometry for the latter. Ultimately, the free energy of adsorption dictates the relative stability of the geometries, but we expect missing entropic contributions from *H*_ads_ to be small and within the error estimates of *H*_ads_ for the systems studied here, becoming prominent only for large molecules or under confinement.

### Gold-standard benchmarks

The predictions from the autoSKZCAM framework for the systems studied in this work can be valuable as a benchmark dataset for non-covalent interactions, which are crucial for modelling the binding of adsorbates to surfaces. These interactions are physically reflected within the interaction energy *E*_int_ contribution to *H*_ads_ (Methods), which quantifies the strength of this binding. Previous studies^22^ have shown that DFAs struggle to consistently describe these interactions for adsorbate–surface systems and that different DFAs can vary over a range of *E*_int_ values exceeding 500 meV, even for a simple system like CO on MgO(001). Here, CCSD(T) is considered a widely trusted approach for treating non-covalent interactions. But while it has become common to generate reference datasets at the CCSD(T) level for small-molecule interaction energies, it has not been possible for adsorbate–surface systems so far. These datasets are commonly used to, for example, parametrize the exchange–correlation functional or dispersion corrections in many modern DFAs. Their poor performance for adsorbate–surface systems arises in part from the lack of available references, particularly those involving metal oxides^23^; this gap can be addressed with CCSD(T)-level references provided by the autoSKZCAM framework.

**Fig. 4:**
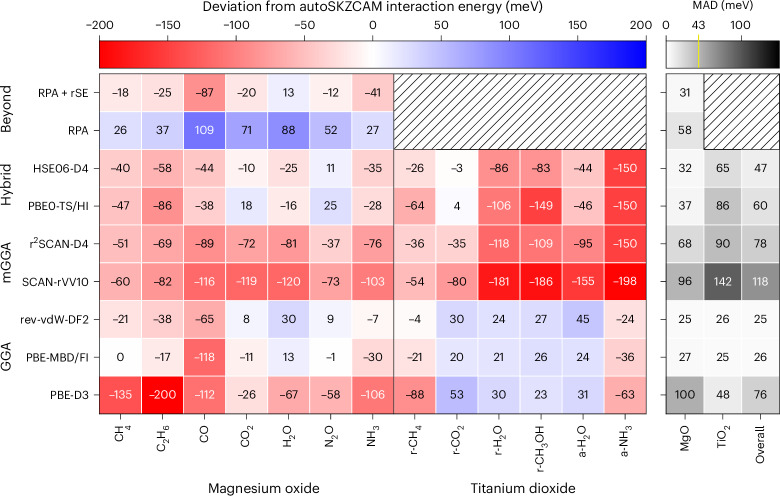
The Surf13 benchmark for lower-level theories. The autoSKZCAM framework interaction energy benchmarks are used to assess a selection of functionals along the hierarchy of DFAs, from the GGA to the meta-GGA (mGGA) and hybrid functionals, as well as the RPA with and without the rSE. The deviation from the autoSKZCAM estimate is given as a colour map, with red and blue indicating overbinding and underbinding, respectively. We consider a range of molecules physisorbed on the MgO(001), rutile (r) TiO_2_(110) and anatase (a) TiO_2_(101) surfaces. The mean absolute deviations (MADs) across all of the systems (labelled ‘overall’), as well as the subsets involving the MgO(001) and TiO_2_ surfaces, are given in grey in the right panel. We indicate the typical ‘chemical accuracy’ of 43 meV in yellow on the colour bar. The autoSKZCAM and DFT interaction energies are tabulated in Supplementary Section 2.

In Fig. 4, a set of DFAs selected from a recent benchmark study^22^ is compared against the autoSKZCAM *E*_int_ benchmarks for the 13 adsorbate–surface systems involving single molecules. The values are tabulated in Supplementary Section 2 and we will refer to this benchmark dataset as Surf13. We do not aim here to provide a comprehensive overview of current DFAs nor to make definitive statements about the performance of different types of exchange–correlation functionals. However, a broad set of exchange–correlation functionals has been considered, starting from the generalized gradient approximation (GGA) and going all the way up to the state-of-the-art random phase approximation (RPA). Each of these exchange–correlation functionals (besides RPA) has been further paired with a wide range of dispersion corrections to improve their description of adsorbate–surface systems. The resulting selection of DFAs includes the workhorse PBE-D3 (ref. ^24^) and newly developed DFAs such as r^2^SCAN-D4 (ref. ^25^) and SCAN-rVV10 (ref. ^26^), as well as sophisticated hybrids and RPA. Of the investigated DFAs, we observe that two GGA-based DFAs (PBE-MBD/FI^27^ and rev-vdW-DF2 (ref. ^9^)) perform best, with a mean absolute deviation of 26 meV and 25 meV, respectively, across all the systems (labelled ‘overall’ in Fig. 4). However, RPA—considered the current state-of-the-art method for surface chemistry—has a mean absolute deviation of 58 meV for the subset of MgO(001) adsorbate–surface systems that were studied. These errors arise from a well-known systematic underbinding of RPA, which is improved by incorporating the renormalized singles contribution (rSE)^28^, instead overbinding with a mean absolute deviation of 31 meV for the MgO(001) adsorbate–surface systems. Unfortunately, the higher cost of RPA prevented its application to the TiO_2_ surfaces and thus, these insights for RPA are limited to only the specific set of molecules adsorbed on MgO(001); broader comments require a more complete dataset involving more surfaces.

These benchmarks provide important insights towards designing improved DFAs. For example, PBE-D3 (ref. ^24^) with zero damping, SCAN-rVV10 (ref. ^26^) and r^2^SCAN-D4 (ref. ^25^) are all found to overbind *E*_int_ for systems involving MgO(001), with the latter two also overbinding for the TiO_2_ surfaces. These observations are commensurate with previous findings, where the overbinding in PBE-D3 has been attributed to an overestimated Mg *C*_6_ dispersion coefficient in the D3 dispersion correction^29^. The SCAN and r^2^SCAN functionals have recently been shown to overbind solids^30^, and this work confirms that this overbinding persists for adsorbate–surface systems, with the rVV10 dispersion correction further exacerbating this overbinding.

## Discussion

**Fig. 5:**
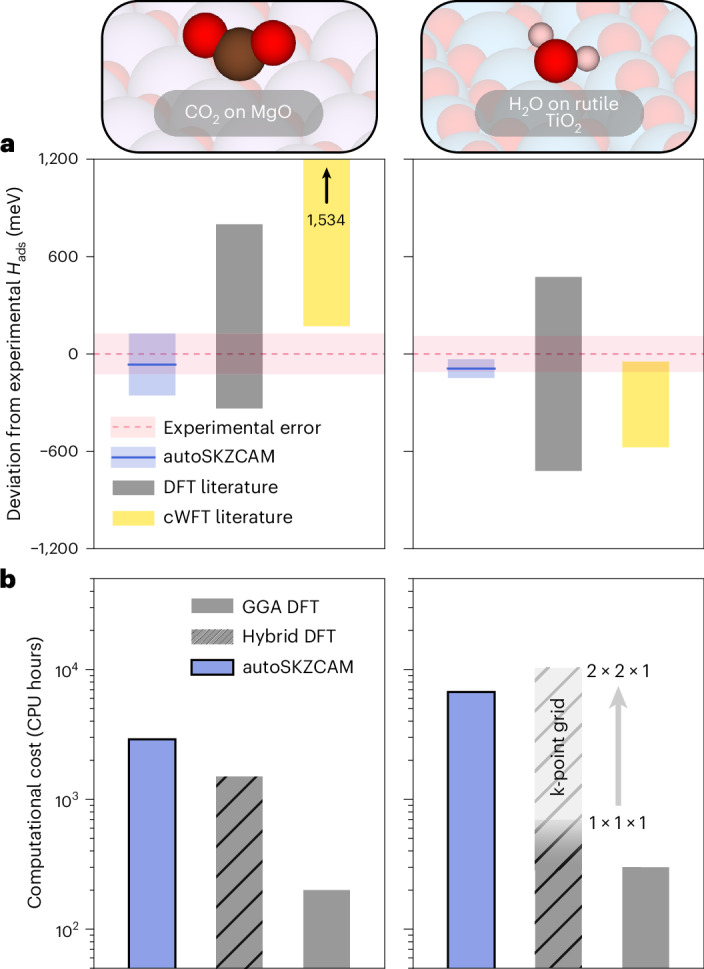
High accuracy at comparable cost to that of periodic hybrid DFT. **a**, For the chemisorbed CO_2_ on MgO(001) and H_2_O on rutile TiO_2_(110), we demonstrate the improved agreement with experimental TPD measurements^14,44^ for the autoSKZCAM framework relative to previous DFT (grey) and cWFT (yellow) data in the literature, both of which are plotted as a range of values, collated in Supplementary Section 13. **b**, We show that the computational cost to calculate the interaction energy is competitive with that of periodic hybrid DFT. For H_2_O on rutile TiO_2_(110), the cost of periodic hybrid DFT can vary depending on the choice of k-point grid (as discussed in Supplementary Section 14), highlighted by the faded region, with changes to the interaction energy on the order of 20 meV. Experimental errors in *H*_ads_ for these systems are based on TPD analysis by Campbell and Sellers^23^, taken as twice the standard deviation in the predicted pre-exponential factor against a test set of ~20 adsorbed molecules (Supplementary Section 11). Simulation errors are the root squared sum of several contributions, described in Supplementary Section 9, with the majority arising from errors using a geometry optimized by DFT, which we estimate as twice the root mean square error from an ensemble of six DFAs.

The agreement achieved in *H*_ads_ values between experiment and the autoSKZCAM framework is not trivial. For example, we show in Fig. 5a and Supplementary Section 13 that DFT estimates, from the literature, on the value of *H*_ads_ for CO_2_ adsorbed on MgO(001) and H_2_O adsorbed on rutile TiO_2_(110) can span a range of nearly 1,000 meV. Beyond the errors in *E*_int_ arising from the choice of DFA (as highlighted in Fig. 4), these errors also stem from the use of unconverged structural models in the embedded cluster or periodic slab approaches. Additionally, electronic structure parameters such as the basis set size, treatment of frozen cores and pseudopotentials must be carefully controlled, with the majority of studies neglecting thermal and vibrational contributions to *H*_ads_. These challenges become more pronounced for methods from cWFT, where ensuring converged electronic structure parameters or structural models is limited by the high computational cost. We highlight this challenge in Fig. 5a, where a range of 528 meV has been observed across cWFT-based studies of H_2_O on rutile TiO_2_(110) and an even larger range of 1,210 meV for CO_2_ on MgO(001), with similar discrepancies noted for CO on MgO(001) (ref. ^31^).

Despite the challenges in applying cWFT methods like CCSD(T) to adsorbate–surface systems, other successful applications appear in the literature, in addition to this work, for example, the collection of molecules adsorbed on MgO(001) as well as zeolites and metal–organic frameworks studied by Sauer and coworkers^6^, and the landmark study by Kubas et al.^7^ tackling five molecules on the rutile TiO_2_(110) surface, both with an embedded cluster approach. More recently, this success has extended towards periodic slab approaches with CCSD(T)^31,32^ thanks to algorithmic and methodological advances. Besides agreeing with these previous estimates, we find that our autoSKZCAM estimates agree with previous quantum diffusion Monte Carlo—another widely trusted cWFT method—estimates for CO on MgO(001) (ref. ^31^) as well as H_2_O on MgO(001) (refs. ^33,34^), upon inclusion of missing geometrical relaxation contributions in the latter. The key advance in the present study is the breadth, size and number of adsorbate–surface systems that can now be tackled, driven by the (1) low cost, (2) general applicability and (3) automated nature of the autoSKZCAM framework. These qualities are enabled by combining the mechanical embedding approach of Sauer and coworkers^6^ with the electrostatic embedding procedure pioneered by Catlow and coworkers^35^ and Reuter and coworkers^7^. The method is made more economical by using CCSD(T) with local approximations.

We highlight the low computational cost of the autoSKZCAM framework in Fig. 5b, where it is compared to periodic DFT—performed using reasonable electronic structure settings^36^—with a hybrid and GGA DFA. The cost to perform the autoSKZCAM framework is competitive with periodic hybrid DFT for both CO_2_ on MgO(001) and H_2_O on rutile TiO_2_(110). Importantly, this cost does not change much with the increase in complexity from a MgO(001) surface to a rutile TiO_2_(110) surface, as the embedding procedure ensures that the largest system (that is, embedded cluster) tackled remains consistent in size across these surfaces. In Supplementary Section 14, we compare our autoSKZCAM framework costs for CO on MgO(001) to previous work. Its automation in the present work (as discussed in Supplementary Section 6g) enabled further levels of mechanical embedding, which led to an overall cost of ~600 central processing unit (CPU) hours to compute *E*_int_ for CO on MgO(001) with the autoSKZCAM framework. This cost is almost one order of magnitude lower than that of RPA and nearly two orders of magnitude lower than a previous (non-automated) application of the SKZCAM protocol^31^ and an efficient periodic CCSD(T) calculation^31,37^, while being more than three orders of magnitude cheaper than periodic diffusion Monte Carlo^31^.

Beyond the applications demonstrated so far, we discuss here the potential of the autoSKZCAM framework to validate experimental results. As shown in Fig. 2, it provides conservative error estimates for *H*_ads_ that are lower than experimental uncertainties for the majority of systems. These experimental values were re-analysed from previous TPD measurements, following Campbell and Sellers^23^, using more accurate system-specific^38^ pre-exponential factors (*ν*). The majority of the experimental error arises from uncertainties in *ν*, with additional minor contributions discussed in Supplementary Section 11. We show that using the original analysis procedure (setting *ν* to a default value of 10^13^) leads to *H*_ads_ values that are in worse agreement with autoSKZCAM, with a root mean square deviation of 102 meV, compared with 58 meV for the system-specific procedure. Going further, the autoSKZCAM framework can shed light on discrepancies between different TPD experiments, as we have found for CO_2_ on MgO(001). Two measurements exist: one reports a weak *H*_ads_ characteristic of physisorption^13^, and the other reports a strong *H*_ads_ indicative of chemisorption^14^. For this system, our autoSKZCAM estimates agree with the chemisorption data but cannot replicate the physisorption results. In Supplementary Section 1b, we highlight inconsistencies in the physisorption experiment that cast doubt on these measurements. When these inconsistencies are accounted for, the experimental data align with our chemisorption estimates.

Before concluding, we should highlight some limitations of the autoSKZCAM protocol. First, the (electrostatic) embedding procedure that underlies the autoSKZCAM framework limits its application to ionic materials. Second, while the accuracy of CCSD(T) has been well validated for small and weakly correlated systems, open questions exist on its applicability towards the binding of larger molecules and surfaces with complex electronic properties. These points are further discussed in the Methods.

## Conclusion

To conclude, we have developed and implemented the autoSKZCAM framework to calculate accurate yet low-cost adsorption enthalpies *H*_ads_ of molecules on the surfaces of ionic materials. This has enabled agreement with experimental measurements for a diverse set of 19 adsorbate–surface systems, beyond the accuracy of any DFA considered, while being at a cost comparable to that of hybrid periodic DFT. We have revealed insights into several of these systems, notably the following: CO_2_ binds on MgO(001) in the long-debated chemisorbed state, and it can take on a tilted configuration on rutile TiO_2_(110), albeit close in stability to a horizontal parallel configuration; N_2_O binds in a horizontal parallel fashion; NO exists as bound dimers on MgO(001); and CH_3_OH and H_2_O form partially dissociated hydrogen-bonded clusters on top of MgO(001). In addition, we show that this dataset—dubbed Surf13—can be used to benchmark DFT exchange–correlation functionals and dispersion corrections, providing direct insights into their performance for adsorbate–surface systems.

This framework has been coded into an open-source package on Github (https://github.com/benshi97/autoSKZCAM), making it a readily available tool to compute accurate reference data for adsorbate–surface systems to facilitate reliable surface chemistry studies (as elaborated in Supplementary Section 10). This reliability will be paramount in complementing experiments towards understanding important catalytic reaction processes, serving to unlock new directions for improving such processes. Furthermore, the framework’s automated nature means it can serve as a standalone tool within a computational catalyst discovery pipeline to screen for new solid catalysts. Similarly, it can be used to provide large databases containing *E*_int_ benchmarks that can be used to directly parametrize improved (machine-learned) DFAs^39^ and electronic structure methods.

Due to their technological relevance and the ready availability of experimental data, this work focuses on metal oxide surfaces, but we expect the autoSKZCAM framework to be applicable to the surfaces of most ionic materials (possessing a bandgap). Evidence in support of this statement is provided in Supplementary Section 6f, where we have calculated the *E*_int_ values of both H_2_O on LiH(001) and acetylene on NaCl(001), reaching agreement with available theoretical (diffusion Monte Carlo and CCSD(T)) estimates in the former and experimental measurements in the latter. Although this work provides new tools for answering questions about the surface chemistry of ionic materials, it cannot tackle many important classes of systems. It will be important to develop new embedded cluster approaches that can treat transition metal surfaces^8,40^ and covalent materials like metal–organic frameworks^41^ and zeolites^6,42^. Furthermore, it is desirable to go beyond a simple (local) harmonic description of *H*_ads_ to treat anharmonicities and non-localized phenomena (that is, adsorption at high temperatures). Towards this end, exciting prospects exist in integrating embedded cluster models with machine-learned interatomic potentials to extend the system sizes tackled and enable finite temperature effects to be incorporated.

## Methods

The autoSKZCAM framework partitions the adsorption enthalpy *H*_ads_ into several key contributions^6^:

1 $${H}_{{\rm{ads}}}={E}_{{\rm{int}}}+{E}_{{\rm{rlx}}}+{E}_{{\rm{ZPV}}}+{E}_{{\rm{T}}}-RT.$$

The interaction energy *E*_int_ is defined as the energetic difference between the adsorbate–surface complex and the separate adsorbate and surface, both of which are fixed at their geometries in the complex. This term is treated efficiently up to the CCSD(T) level through the SKZCAM protocol^22,31,45^ developed by Shi, Kapil, Zen, Chen, Alavi and Michaelides^45^. It is made more economical by employing recent local correlation approximations to CCSD(T) (that is, LNO-CCSD(T)^46^ and DLPNO-CCSD(T)^47^). The relaxation energy *E*_rlx_ is the energy for the fixed adsorbate and surface to relax into their equilibrium geometries, while the zero-point vibrational and thermal contributions are given by *E*_ZPV_ and *E*_T_, respectively. We show in Supplementary Section 8 that these remaining contributions can be estimated effectively with DFT by employing an ensemble of six widely used DFAs. When studying clusters or monolayers, additional terms are used for the lateral interaction energy between the molecules, which are treated at the CCSD(T) level, as discussed in Supplementary Section 7. For chemisorbed CO_2_ on MgO(001), there is also an additional term (coming out of *E*_rlx_) for the large conformational energy change in the CO_2_ molecule. For the dissociated H_2_O and CH_3_OH clusters, we include a dissociation contribution *E*_diss_, which accounts for the energy change arising from dissociation of the parent molecularly adsorbed cluster.

The autoSKZCAM framework has been coded within an open-source package on Github (https://github.com/benshi97/autoSKZCAM), with examples and documentation provided within. It makes extensive use of the QuAcc workflow library^48^, which can be used to generate the relaxed adsorbate–surface geometries starting from just a molecule and crystal unit cell, as discussed in Supplementary Section 10. Scripts are also provided to perform a random structure search to obtain candidate adsorption configurations if the adsorption configuration is not known.

### Accurate interaction energies with the SKZCAM protocol

The most common embedding approach for adsorbate–surface systems involving ionic materials is electrostatic embedding, where the system is modelled as a central ‘quantum’ cluster surrounded by a field of point charges representing the long-range interactions from the rest of the surface. This approach has been applied to systems ranging from simple ionic materials^49^ to challenging quantum materials^50^, not only on their surfaces but in the bulk^51^, as well as on steps, edges and kinks^52^. However, designing efficient quantum clusters amenable to methods such as CCSD(T) while being converged to the bulk limit is not trivial, requiring considerable manual effort and chemical intuition.

The SKZCAM protocol aims to address the challenges with applying electrostatic embedding. It defines rubrics for a converging series of clusters that can be generalized to the adsorption of molecules on diverse sets of ionic crystals and their surface terminations. The resulting series of clusters typically exhibit a smooth convergence of *E*_int_ along the series of clusters, allowing the bulk infinite-size limit *E*_int_ to be reached by extrapolating from a set of small clusters, as described in Supplementary Section 6. We use the lower-level MP2 perturbation theory to perform this (bulk) extrapolation with moderately sized clusters (<75 atoms). This MP2 prediction can then be elevated to the CCSD(T) level from smaller clusters (<35 atoms) through an ONIOM-like^53^ mechanical embedding approach. While previously requiring a notable amount of user intervention, this protocol has been automated within the present work, eliminating any manual intervention and facilitating use with a wide range of adsorbate–surface systems involving ionic materials. Moreover, this automation has allowed CCSD(T) to be mechanically embedded within additional ONIOM layers, corresponding to more affordable (that is, smaller basis sets or a bigger frozen core) levels of theory, as discussed in Supplementary Section 6g. This has reduced its cost by one order of magnitude compared with previous works (Supplementary Section 14), making it now competitive with periodic hybrid DFT.

The electrostatic embedding environment was constructed using Py-ChemShell v.20.0 (ref. ^35^), setting formal point charges (that is, Ti4^+^, Mg^2+^ and O^2–^) in a 50 Å (60 Å) field around the quantum cluster centre for the MgO(001) surface (rutile TiO_2_(110) or anatase TiO_2_(101) surface). A further region of effective core potentials was placed on the positive point charges within 4 Å (6 Å) of the quantum cluster to prevent spurious charge leakage. MP2 was performed within ORCA v.5.0.3 (ref. ^54^) with the resolution-of-identity approximation, while CCSD(T) was performed within MRCC^55^ using the local natural orbital (LNO) approximation^46^. A two-point (double-zeta/triple-zeta) complete basis set extrapolation^56^, together with counterpoise corrections, was used to calculate the MP2 and LNO-CCSD(T) *E*_int_. Subsequent basis set and core–valence correlation contributions were added at the MP2 level, as discussed in Supplementary Section 5.

### Robust geometrical and vibrational contributions with an ensemble of DFAs

The remaining terms (that is, *E*_rlx_, *E*_ZPV_ and *E*_T_) form a small overall contribution to *H*_ads_ that can be adequately treated with DFT. These terms are estimated by employing an ensemble of six widely used DFAs of differing exchange–correlation functionals (up to hybrids) and dispersion corrections. The six DFAs used for MgO(001) were PBE-D2[Ne], revPBE-D4, vdW-DF, rev-vdW-DF2, PBE0-D4 and B3LYP-D2[Ne], where [Ne] indicates the use of neon *C*_6_ parameters for the Mg atoms. The rutile TiO_2_(110) and anatase TiO_2_(101) surfaces used PBE-TS/HI, revPBE-D4, vdW-DF, rev-vdW-DF2, r^2^SCAN-rVV10 and HSE06-D4. Through averaging, this choice can provide estimates with corresponding 2*σ* standard deviations as an error measurement—typically much better than chemical accuracy (Supplementary Section 8). We discuss in Supplementary Section 8c how this ensemble can be further used to assess inaccuracies from using a DFT geometry for the CCSD(T) treatment. As a result, this DFA ensemble allows for conservative errors bars on the final *H*_ads_ estimate when comparing against experiments.

The DFT calculations were performed in the Vienna Ab initio Simulation Package v.6.3.0 (refs. ^57,58^). Of the 13 systems involving MgO(001), we used a 4 × 4 supercell for all systems except for the C_6_H_6_, CH_3_OH cluster and H_2_O cluster, where an 8 × 8 supercell was used. The MgO(001) surface slab consisted of four layers, with the bottom two layers fixed. The rutile TiO_2_(110) surface slab consisted of a 4 × 2 supercell with five tri-layers (and the bottom three fixed), while the anatase TiO_2_(101) surface slab consisted of a 3 × 1 supercell with four tri-layers and the bottom layer fixed. All surfaces incorporated 15 Å of vacuum with geometrical relaxation performed with a force convergence cut-off of 0.01 eV Å^−1^. A plane-wave kinetic energy cut-off of 600 eV was used, which was reduced to 520 eV for the hybrid HSE06-D4 calculations on the TiO_2_ surface systems. A 2 × 2 × 1 Γ-centred Monkhorst–Pack k-point mesh was used for the MgO(001) surface (reduced to only the Γ point for the larger surface), and a 2 × 2 × 1 mesh for the rutile (110) surface and 3 × 3 × 1 mesh for the anatase (101) surface. To calculate the *E*_ZPV_ and *E*_T_ contributions, the contributions from individual vibrational modes were computed with the quasi-rigid-rotor harmonic oscillator approximation^59^. Further details, particularly the parameters for the benchmarks in Fig. 4, are given in Supplementary Sections 2 and 8a.

### Error estimates

The experimental estimates were largely taken from single-crystal TPD measurements compiled by Campbell and Sellers^23^, where the effects of surface disorder or defects are expected to be minimal. The error bars on these measurements correspond to a 95% confidence interval on the experimental pre-exponential (*ν*) factor, coming from predictions for the standard entropy of the adsorbate by Campbell and Sellers^38^. A similar confidence interval has been calculated for the the individual terms in the autoSKZCAM framework and the overall *H*_ads_ estimate, as discussed in Supplementary Sections 11 and 9, respectively. We connect static adsorption energies to *H*_ads_ using the quasi-rigid-rotor harmonic oscillator method^59^, which improves over the standard harmonic approximation for treating low-lying vibrational modes of the adsorbate. We expect the error bars (from the DFT ensemble) we have computed on these thermal contributions to be greater than or comparable to the remaining anharmonic contributions.

### Limitations

The key limitation of the autoSKZCAM framework is that it can treat the surfaces of only ionic materials. Going beyond electrostatic embedding towards (quantum) embedding approaches^60^ that couple the environment to the quantum cluster through variables such as the density, the single-particle Green’s function or the single-particle density matrix would allow covalently bonded and metallic systems to be treated. This improved treatment of the boundaries can also enable smaller and more efficient (high-level) quantum clusters to be used. However, getting the coupling of the quantum cluster to the environment right is currently not trivial, often requiring several parameters to be converged. Furthermore, these calculations^61,62^ build on a prior DFT or Hartree–Fock (HF) calculation that has to be performed on the full adsorbate–slab system—typically a periodic model, which can be computationally expensive. Recent work^63^ has highlighted that it is possible to overcome this need for periodic calculations through a multilevel embedding procedure by combining the quantum embedding approaches with efficient (cluster) surface models that can be generated by, for example, the SKZCAM protocol.

Limitations also exist with using CCSD(T) as the target level of theory for the autoSKZCAM framework. While it is trusted for studying small and weakly correlated systems, open questions exist on the applicability of CCSD(T) towards more challenging systems. For example, it cannot treat systems without a bandgap (notably metals), and it performs poorly for open-shell molecules of radical character. More recently, it has been shown to disagree with quantum diffusion Monte Carlo—another widely trusted method—when studying large dispersion-bound molecules of *π*–*π* character^64,65^ and medium-sized hydrogen-bonded molecules^66^. Moreover, beyond the adsorbate, challenges can exist in describing the surface of some transition metal oxides, as they may exhibit antiferromagnetic or more exotic (strongly correlated) properties. To study such systems accurately, CCSD(T) must be replaced with a more appropriate level of theory, such as multi-reference approaches or quantum embedding approaches (for example, dynamical mean-field theory and density matrix embedding theory^67^).

## Online content

Any methods, additional references, Nature Portfolio reporting summaries, source data, extended data, supplementary information, acknowledgements, peer review information; details of author contributions and competing interests; and statements of data and code availability are available at 10.1038/s41557-025-01884-y.

## Supplementary information

Supplementary Information Supplementary Figs. 1–17, Tables 1–37 and Sections 1–14.

## Data Availability

Data that support the points made throughout the text are available in the article and Supplementary Information. The input and output files associated with this work and all analysis are available via GitHub at https://github.com/benshi97/Data_autoSKZCAM and via Zenodo at 10.5281/zenodo.15651018 (ref. ^68^). The files can also be viewed (and analysed) online via Colab at https://colab.research.google.com/github/benshi97/Data_autoSKZCAM/blob/master/analyse.ipynb.

## Code availability

The autoSKZCAM framework is freely available via GitHub at https://github.com/benshi97/autoSKZCAM, with documentation found at https://www.benjaminshi.com/autoSKZCAM/ containing instructions and examples on how to run the code.
