# Supplementary material for: An accurate and efficient framework for modelling the surface chemistry of ionic materials
Source: Nat Chem. 2025 Aug 13;17(11):1688–95. doi: 10.1038/s41557-025-01884-y (PMC12580338; doi:10.1038/s41557-025-01884-y)
Supplement: Supplementary file 1 — Supplementary Figs. 1–17, Tables 1–37 and Sections 1–14. [file 41557_2025_1884_MOESM1_ESM.pdf]

nature chemistry

Supplementary information

https://doi.org/10.1038/s41557-025-01884-y

An accurate and efficient framework for modelling the surface chemistry of ionic materials

In the format provided by the authors and unedited

CONTENTS

Part I: Additional results and discussion 5

1. Insights into adsorption configuration 6
   - A. Cluster CH_3OH and H_2O on MgO(001) 6
   - B. CO_2 on MgO(001) 8
   - C. NO on MgO(001) 9
   - D. CO_2 on TiO_2 rutile(110) 10
   - E. N_2O on MgO(001) 12
   - F. Previously debated systems 13

2. A benchmark dataset for non-covalent interactions of adsorbate–surface systems 14
   - A. Computational details for the DFA benchmark 14

Part II: Supporting data 18

3. The approach to calculating the adsorption enthalpy 19

4. The adsorbate–surface complexes studied in this work 20

5. Computational details for correlated wave-function theory 23

6. Interaction energies computed with the SKZCAM protocol 24
   - A. Generating a systematic series of clusters within electrostatic embedding 24
   - B. Extrapolating towards the bulk limit with the series of clusters 27
   - C. Multilevel ΔCC contribution through mechanical embedding with small clusters 38
   - D. Further multilevel contributions for basis set and semi-core electron correlation 41
   - E. The final E_{int} estimates and their error bars 44
   - F. Validating the SKZCAM protocol beyond metal-oxides 46
   - G. Improvements to SKZCAM protocol in present work 48

7. Contributions for the cohesive and conformational energy from cWFT in selected systems 49
   - A. Conformational energy of the chemisorbed CO_2 on MgO(001) 49
   - B. Cohesive energy in CH_4 and C_2H_6 monolayers on MgO(001) 52
   - C. Cohesive and dissociation energy in H_2O and CH_3OH clusters on MgO(001) 55
   - D. Cohesive energy of NO dimer on MgO(001) 56

8. Geometry relaxation and enthalpic contributions from a DFT ensemble 59
A. Computational details for periodic density functional theory 59
B. The relaxation energy 61
C. Estimating geometrical errors 64
D. Zero-point vibrational and enthalpic contributions 71
E. Dissociation energy for the H_2O and CH_3OH clusters 72

9. Final autoSKZCAM estimates 75
A. Validating autoSKZCAM error estimates 77

10. Automation of the autoSKZCAM framework 78
A. QuAcc computational workflow details 78
B. Automated SKZCAM protocol 80

11. Analysing experimental estimates and techniques 81

12. Comparison of H_{ads} between autoSKZCAM and experiments 85

13. Previous computational literature 89

14. Benchmarking the cost of the autoSKZCAM framework 91

References 94

We provide additional supporting data as well as contextual information to the main text here. All output files are provided on Github [1], with a corresponding Jupyter Notebook file that analyses all of the data. This data can also be viewed and analysed on the browser with Google Colab.

Within this supplementary data, we start by providing more detail on two key developments of this work:

- Firstly, we highlight the atomic-level insights provided by the autoSKZCAM framework into the adsorption configuration for several of the studied systems in Section 1, together with a discussion of the prior literature.

- Then, we tabulate the CCSD(T)-level dataset of references on the interaction energy for the set of adsorbate–surface system studied within this work, provided in Section 2. These values can serve as a useful benchmark tool for assessing the performance of newly developed density functional approximations as well as approximate correlated wave-function methods.

In the second part to the supplementary information, we provide more elaborate details and concrete numbers to support the claims made within the main text:

- We discuss the approach we take towards computing the adsorption enthalpy in Section 3 which forms the basis of the autoSKZCAM framework developed within the present work.

- We show the adsorbate–surface complexes studied within this work in Section 4.

- We give the details of the correlated wave-function methods [MP2 and CCSD(T)] used in this work in Section 5.

- We describe the SKZCAM protocol – used to calculate the interaction energy contribution to the adsorption enthalpy – in Section 6. The set of clusters generated by this protocol and the MP2 and CCSD(T) estimates for each cluster are also provided.

- We discuss additional contributions calculated at the CCSD(T)-level, such as the conformational energy and cohesive energy (for the clusters and monolayers) contributions to the final adsorption enthalpy in Section 7.

- We describe how the remaining contributions to the adsorption enthalpy – geometrical relaxation, zero-point vibrational and thermal – are calculated using an ensemble of density functional approximations in Section 8.

- We provide the final adsorption enthalpy estimates made with the autoSKZCAM framework in Section 9 and make some additional validation tests on its reliability in Section 9 A.
- We describe how the autoSKZCAM framework has been automated using the QuAcc computational materials science workflow library in Section 10.
- We describe how we analyse accurate experimental adsorption enthalpies with reliable error bars in Section 11.
- The final autoSKZCAM adsorption enthalpy estimates are compared to experiments in Section 12.
- These estimates are further compared to the previous literature in Section 13.
- Finally, we benchmark and highlight the low-cost of the autoSKZCAM framework relative to DFT in Section 14.

Part I: Further insights and discussion

1. INSIGHTS INTO ADSORPTION CONFIGURATION

We now discuss specific subset of systems within the 19 studied adsorbate–surface systems (see Section 4) where there have been debates on the adsorption configuration. We apply the autoSKZCAM framework to compute H_{ads} for each of these systems and show that the lowest energy configuration has an H_{ads} that agrees with experiment; with all other configurations predicted to be less stable with an H_{ads} that cannot reproduce experiment.

A. Cluster CH_3OH and H_2O on MgO(001)

For both CH_3OH and H_2O on MgO(001), we have observed that it is necessary to account for H-bonded clustering of the molecules on the surface together with partial dissociation of the cluster to achieve agreement on H_{ads} with experiments. In Fig. 1, we compare the H_{ads} with the revPBE-D4 [4, 5] density functional approximation (DFA) for several adsorption configurations of CH_3OH on MgO(001), involving a single molecule up to clusters adsorbed on the surface. This DFA was chosen because it can successfully reproduce autoSKZCAM estimates in Extended Data Fig. 1 and 2 of the main text. For single molecules, we considered both a tilted [6, 7] and parallel [8, 9] adsorption configuration. These have been proposed within previous literature and we find that the tilted configuration is more stable, albeit unable to reproduce experiment. This hints at missing contributions found in e.g., H-bonded and dissociated clusters. For the H-bonded clusters, we have considered the lowest-energy, as discovered through a random structure search, geometries for the dimer, trimer and tetramer, while dissociation is induced by moving an H-atom onto a nearby O atom. There is significant stabilisation for the H-bonded tetramer of CH_3OH relative to the dimer or trimer, as it can form a complete H-bonded network commensurate with the underlying geometry of the MgO(001) surface. However, this is still insufficient for reproduce the experimental H_{ads}. There is further stabilisation when dissociation is induced. While dissociation is isoenergetic (to within 2 meV) to the molecular form for the dimer, there is stabilisation of 43 meV for the trimer, which goes up to 78 meV for the tetramer. This stabilisation brings the dissociated tetramer H_{ads} into agreement with experiments. Such stabilisation upon dissociation has also been observed for clusters of H_2O, where we find that dissociation stabilises the H_2O tetramer by 81 meV (Extended Data Fig. 2 of the main text), which would bring the autoSKZCAM estimate into excellent agreement with experiment.

In Extended Data Figs. 1 and 2 of the main text, we plot the H_{ads} for H_2O and CH_3OH

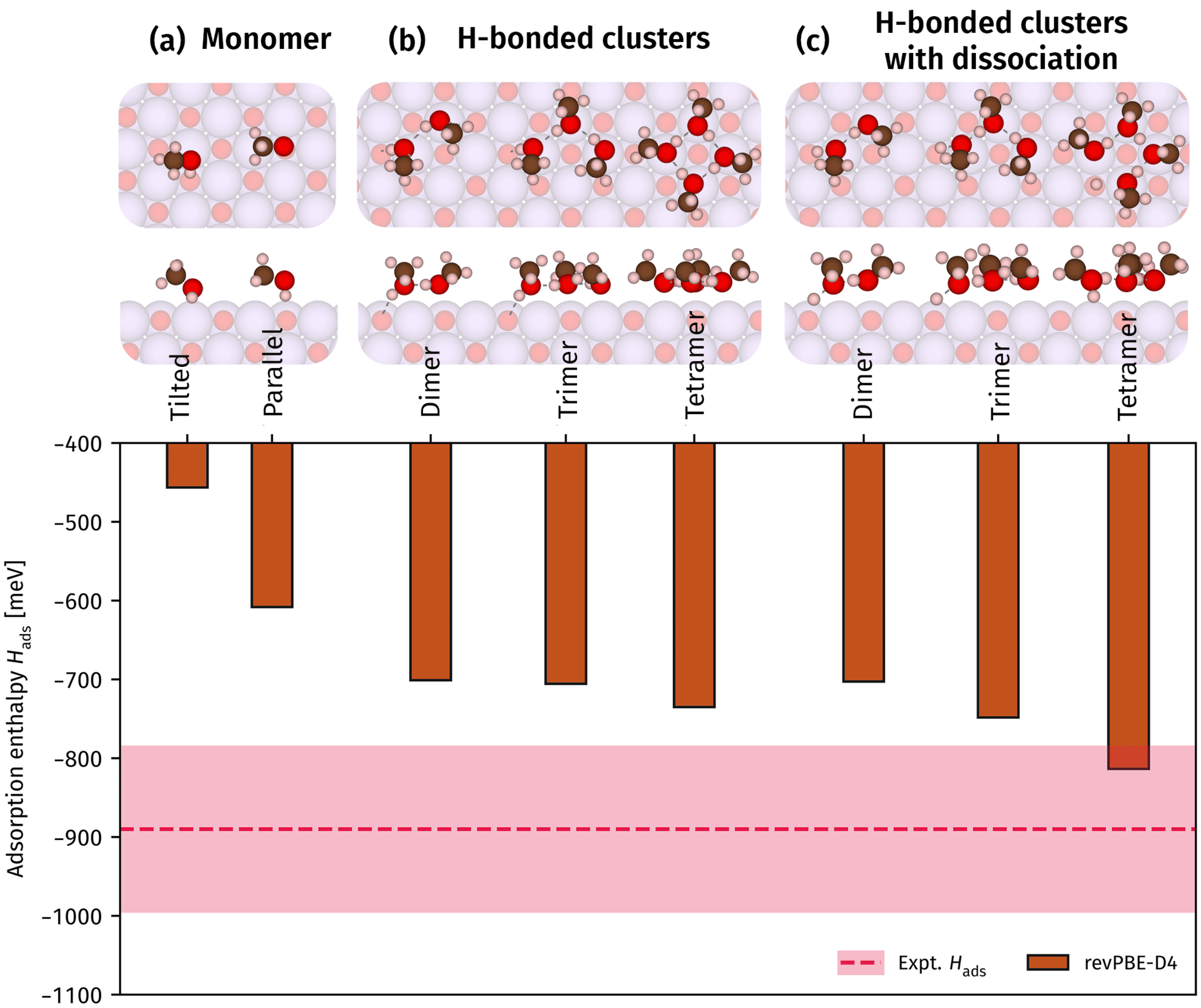

FIG. 1: Comparison of the H_{ads} (per molecule) calculated with the revPBE-D4 functional for the adsorption of (a) monomer CH_3OH on MgO(001), (b) H-bonded clusters and (c) H-bonded CH_3OH cluster with partial dissociation. We consider the lowest energy dimer, trimer and tetramer adsorption configurations. These are compared against experimental TPD measurements by Günster *et al.* [2]. The experimental error on H_{ads} is based on temperature programmed desorption analysis by Campbell and Sellers [3], taken as twice the standard deviation in the predicted pre-exponential factor against a test set of ∼20 adsorbed molecules (Section 11). Simulation errors are the root squared sum of several systematic contributions described in Section 9, with the majority arising from errors using a geometry optimised by density functional theory, which we estimate as twice the root-mean-squared-error from an ensemble of 6 density functional approximations.

on MgO(001) for the monomer and tetramer configurations using the autoSKZCAM framework and a set of density functional approximations. As discussed in Section 8 E, we predict the H_{ads} for the dissociated form of the tetramer by using the DFT ensemble to calculate E_{diss} – the stabilisation energy to form the dissociated cluster from the (molecular) H-bonded cluster. This enables excellent agreement to the experimental H_{ads} for both H_2O and CH_3OH.

Our insights are in agreement with experimental evidence, which suggests that even at low coverage limits, CH_3OH molecules will form 2D islands [10]. In particular, we suggest that these 2D islands contain H-bonded networks together with partial dissociation. It should be noted that while an H_{ads} value was obtained for a single-monomer of H_2O in Refs. 11 and 12, this was done by subtracting the lateral interactions (including both H-bonding and partial dissocation) of a 2D monolayer of water from the monolayer H_{ads} of H_2O. Physically, we expect a similar behaviour for H_2O molecules on MgO(001), forming clusters even at the low coverage limits.

B. CO_2 on MgO(001)

The adsorption of CO_2 on MgO has been the subject of many theoretical and experimental studies over the years. These have largely revolved around a physisorbed or chemisorbed ('monodentate') state. Within experiments, early work by Meixner *et al.* [13] used laser-induced thermal desorption experiments to predict an H_{ads} of −431 meV, which was attributed to a physisorbed state due to its low (absolute) value. However, a TPD experiment by Chakradhar and Burghaus [14] came to an estimate of −664 meV on H_{ads} and attributed this to the formation of surface carbonates (i.e., chemisorption) as confirmed via XPS, with no evidence of a physisorbed state. On the simulations front, there are several studies which have found the chemisorbed state to be completely unstable [15, 16], predicting a physisorbed state [17–20], while many have also predicted a chemisorbed state to show significant stability [21–23].

We use the autoSKZCAM framework to calculate the H_{ads} for both the physisorbed and chemisorbed structure in Extended Data Fig. 3 of the main text. We find that the autoSKZCAM framework comes into good agreement with the experiment of Chakradhar and Burghaus [14] for the chemisorbed state while it does not come into agreement for the physisorbed state. The DFAs all predict the chemisorbed state to be more stable than the physisorbed state by more than 200 meV, except for vdW-DF, where the differences are less than 30 meV. It can be seen that while the chemisorbed state is predicted to be the most stable by all DFAs, most of the DFAs are unable to reach agreement with experiment. Similarly, most DFAs are unable to match

with the experiment by Meixner *et al.* for the physisorbed state. This suggests potential errors within the original measurements by Meixner *et al.*. For example, Chakradhar and Burghaus have surmised that the temperature reading (~120 K) of Meixner *et al.* was 'un-calibrated' as the low desorption temperatures do not agree with previous TPD measurements [24, 25] including their own (~230 K). In fact, data by Meixner *et al.* indicates a 'lack of surface mobility' of the CO_2, which points towards a chemisorbed state. If the measurements by Meixner *et al.* were re-analysed with a desorption temperature of 230 K, their experiment would predict H_{ads} of -826 ± 94 meV, in good agreement with our autoSKZCAM predictions. Beyond previous TPD measurements, there has been significant recent interest in MgO for CO_2 storage [26, 27], with the chemisorbed state now supported with new evidence from NMR experiments [28, 29].

C. NO on MgO(001)

The adsorption of nitric oxide on the MgO(001) surface has been widely studied by both experiments and computational simulations. Within the computational literature, a wide range of adsorption configurations have been proposed, which we show in Fig. 3 of the main text. For each given geometry, most studies (see Table 35) employing DFT have found E_{ads} in a range in agreement with the experimental H_{ads} estimate [30]. In particular, most studies have looked at the adsorption of monomers on the surface. On the other hand, experiments have largely pointed towards the absence of monomers on the surface. For example, EPR [31] indicates that only 0.5% of sites contained NO monomers (bonded mostly to defects), with FTIR [32] showing that most NO species exist as a closed-shell diamagnetic *cis*-$(NO)_2$ species, hereafter dubbed the NO dimer.

In Fig. 2, we have studied all proposed (monomer and dimer) geometries of NO on the MgO(001) surface with the autoSKZCAM framework and the ensemble of DFAs (providing specific values in Table 1). We find that the monomer species are not as stable as many DFAs predict, being 100 meV higher in H_{ads} than the NO dimer. On top of being the configuration with the lowest H_{ads}, the autoSKZCAM framework also predicts an H_{ads} which reproduces experiment, coming into agreement with previous experimental evidence that NO exists in its *cis*-$(NO)_2$ geometry on top of the MgO surface. In addition, our estimates show that monomers are unlikely to exist on the terrace sites at low temperatures, with only the NO dimer expected to form.

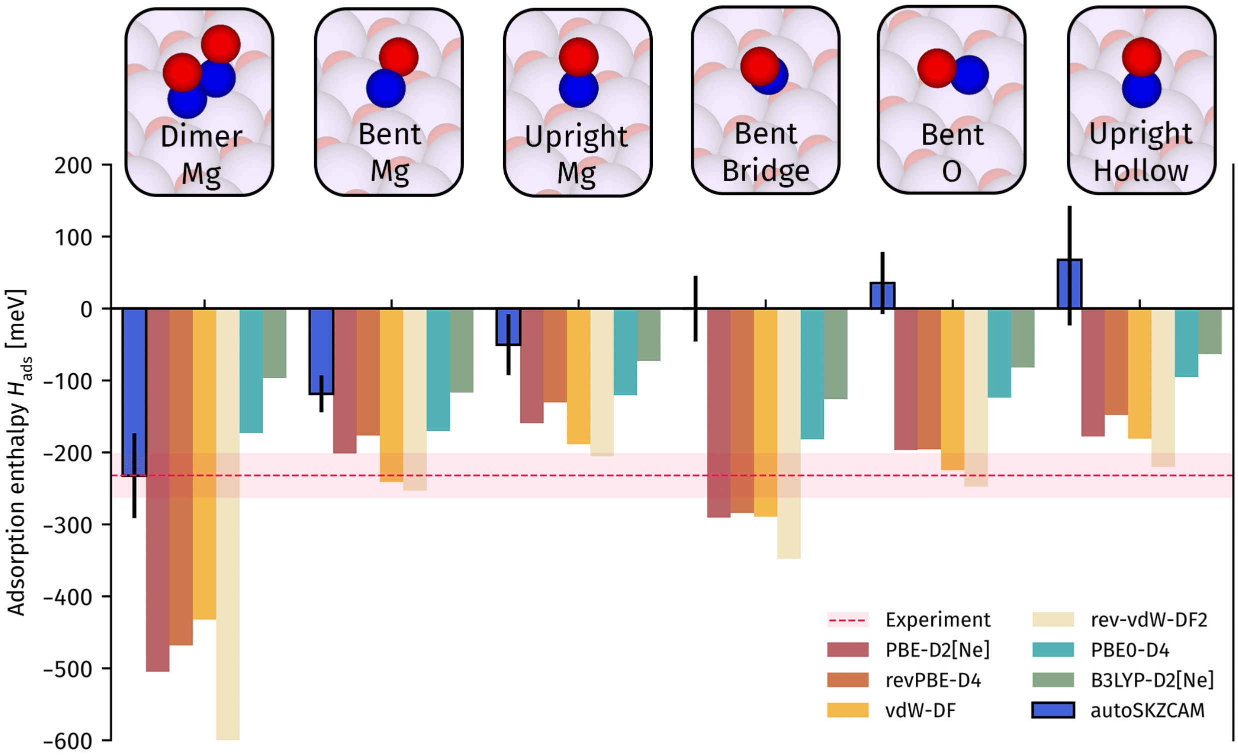

FIG. 2: Comparison of H_{ads} (per molecule) calculated with the autoSKZCAM framework and several DFAs (from the ensemble) for the various adsorption configurations of NO on the MgO(001) surface. These are compared against experimental TPD measurements by Wichtendahl *et al.* [30]. The experimental error on H_{ads} is based on temperature programmed desorption analysis by Campbell and Sellers [3], taken as twice the standard deviation in the predicted pre-exponential factor against a test set of ∼20 adsorbed molecules (Section 11). Simulation errors are the root squared sum of several systematic contributions described in Section 9, with the majority arising from errors using a geometry optimised by density functional theory, which we estimate as twice the root-mean-squared-error from an ensemble of 6 density functional approximations.

D. CO_2 on TiO_2 rutile(110)

The adsorption of CO_2 on TiO_2 rutile(110) has been the subject of many theoretical and experimental studies over the years. Besides one study [33], the majority of computational simulations have demonstrated CO_2 on TiO_2 rutile(110) to take on a tilted geometry on top of the surface. Similarly, barring one experiment, the majority of experiments have pointed towards a tilted geometry on the rutile(110) surface. A study by Sorescu *et al.* [34] found that a simulated STM image of the parallel configuration was more consistent with experimental STM

TABLE 1: Comparison between autoSKZCAM and 6 DFAs in their predicted H_{ads} (in meV) for the different configurations of NO on MgO(001). The experimental H_{ads} is $-232{\pm}31$ meV.

	autoSKZCAM	PBE-D2[Ne]	revPBE-D4	vdW-DF	rev-vdW-DF2	PBE0-D4	B3LYP-D2[Ne]
Dimer	-232 ± 59	-505 ± 5	-468 ± 5	-432 ± 5	-607 ± 5	-173 ± 5	-96 ± 5
Bent-Mg	-119 ± 26	-201 ± 3	-177 ± 3	-241 ± 3	-253 ± 3	-170 ± 3	-117 ± 3
Vertical-Mg	-50 ± 42	-159 ± 3	-130 ± 3	-189 ± 3	-205 ± 3	-120 ± 3	-73 ± 3
Bent-Bridge	0 ± 46	-291 ± 5	-284 ± 5	-289 ± 5	-348 ± 5	-182 ± 5	-126 ± 5
Bent-O	35 ± 43	-197 ± 3	-196 ± 3	-225 ± 3	-247 ± 3	-124 ± 3	-82 ± 3
Vertical-Hollow	68 ± 91	-178 ± 6	-148 ± 6	-181 ± 6	-220 ± 6	-95 ± 6	-63 ± 6

images. However, a later study with some of the original authors showed a preference for a tilted geometry [35] which rotates about the z-axis to diffuse across the TiO_2 rutile(110) surface. This was later confirmed by further independent studies with STM [36] and FTIR [37].

In Extended Data Fig. 4, we have compared the H_{ads} computed with the autoSKZCAM framework (and several DFAs) against experiment for the parallel and tilted geometry. We find that the tilted geometry is more stable than the parallel geometry by 45 meV, albeit with some overlap in their error bars. With the tilted geometry, we are able to reach agreement with experiment, providing strong evidence that it is the expected geometry on the TiO_2 rutile(110) surface, although the small energy difference with the parallel state suggests that under normal temperatures, it can easily move into this metastable state, commensurate with its easy diffusion observed in experiments. We find that all of the DFAs also predict a more stable tilted geometry, although revPBE-D4 predicts the two geometries to be nearly isoenergetic with a 0.3 meV difference.

Our work has also allowed us to re-examine some of the previous experimental and computational work that have predicted a more stable parallel adsorption configuration. The experiment of Sorescu *et al.* [34] with STM had indicated a more consistent agreement towards a parallel geometry. However, this is in disagreement with later STM studies and recent work [38] have actually simulated the STM images of a tilted, parallel and vertical geometry of CO_2 on rutile(110). The differences between the simulated STM images were found to be minor between the adsorption configurations and in fact, the low resolution of STM (in general) meant that they

TABLE 2: Comparison of the E_{ads} in meV of the CO_2 on TiO_2 rutile(110) adsorption configurations from previous DFT studies using PBE-D3 (with zero-damping).

	Parallel	Tilted	Δ
Sorescu et al. [39]	-371	-389	-18
Kubas et al. [33]	-446	-397	49
Dohnalek et al. [36]	-420	-450	-30
This work	-345	-382	-37

found the vertical geometry to be most consistent, in disagreement with Sorescu *et al.*, and found to be the least stable configuration from their simulations [38]. This overall suggests that STM does not have the resolution to discern the geometry of CO_2 on rutile(110). The computational simulation by Kubas *et al.* [33] was the only work we found which predicted a parallel geometry to be lower in energy than the tilted geometry, using not only DFT but also DLPNO-CCSD(T) In Table 2, we show that the surface model which they used - an embedded cluster approach - differs from previous DFT simulations as well as our simulations for the PBE-D3 geometry. Specifically, we have compared PBE-D3 predictions using their embedded cluster model with periodic slab models from two previous DFT simulations as well as our own periodic DFT calculations. All the slab calculations come into agreement that the tilted geometry is between 18 meV to 37 meV more stable than the parallel geometry, while Kubas *et al.* predicts it to be less stable by 49 meV. Similarly, all other studied functionals within their work have predicted an unstable parallel geometry, while the autoSKZCAM framework calculations as well as periodic DFT calculations from this work suggest a tilted geometry.

E. N_2O on MgO(001)

N_2O on MgO(001) is an example of a system that has been very sparsely studied within previous computational simulations, with Scagnelli *et al.* [40] predicting no binding for a geometry with O pointing towards the Mg atom on the MgO surface, while Huesges *et al.* [41] found a tilted geometry with either N or O pointing towards the Mg atom on the MgO surface that are roughly isoenergetic (to within 10 meV). With our DFT ensemble, we find that the tilted geometry with the O pointing towards the Mg atom relaxes to a geometry close to parallel to the surface, such

that both N and O close to an Mg atom. This was confirmed through an additional random structure search, which did not find a tilted O-down geometry, Thus, we compare the parallel configuration with a (N-down) tilted configuration in Extended Data Fig. 5 of the main text. Both the autoSKZCAM framework and the 6 studied DFAs predict the parallel configuration to be the lowest in energy, with autoSKZCAM coming into agreement with the experimental H_{ads}. The variation between DFAs is relatively small (within 70 meV), with four of the six DFAs lying within the experimental H_{ads} error bars.

F. Previously debated systems

Most of the other systems within this work have got geometries that are now well-resolved, however some of these systems were previously topics of debate and we briefly mention some of these systems. For example, it was previously debated whether the CO molecule adsorbs with the C or O atom pointing towards the Mg atom within early simulations [42] but this was later understood to arise from errors with using Hartree-Fock theory with a small basis set [43]. Another system which has been previously under debate is the arrangement of the alkane monolayers on the MgO(001) surface. In particular, for methane, it was not known whether the molecules take up a dipod or tripod [44, 45] configuration, containing two and three H atoms pointing downwards respectively. Furthermore, there were questions whether the methane molecules were rotated with respect to its neighbours [46]. Both of these questions were resolved through a combination of experiments [47, 48] and theory [46, 49]. Similarly, the ethane monolayer has been determined by LEED [50–52] to take its current configuration in Fig. 4, which is in agreement with work by Alessio *et al.* [53]. As seen in Fig. 2 of the main text, we are able achieve excellent agreement to experiment on the H_{ads} for both alkane monolayers on MgO.

2. A BENCHMARK DATASET FOR NON-COVALENT INTERACTIONS OF ADSORBATE–SURFACE SYSTEMS

We have highlighted in Fig. 4 of the main text the possibility to use the numbers generated by the autoSKZCAM framework to benchmark the performance of density functional approximations for adsorbate–surface systems – currently lacking [54]. Specifically, the final E_{int} estimates used in Fig. 4 of the main text are given in Table 3. This forms a database covering a range of adsorbate–surface interactions on three prototypical ionic surfaces. Such databases [55, 56] have been used to calibrate the performance of modern density functional approximations. The poor performance of many sophisticated (hybrid) DFAs in Fig. 4 of the main text can be attributed to the lack of adsorbate–surface benchmarks to calibrate them. The corresponding geometries used to calculate E_{int} are available in the Github repository [1] at: https://github.com/benshi97/Data_autoSKZCAM/tree/master/Data/Miscellaneous/DFT_Comparison/Geometries for comparison to the benchmark values in Table 3.

A. Computational details for the DFA benchmark

We have compared a set of 8 DFAs against the E_{int} calculated by the SKZCAM protocol in Section 6. This was performed for a set of adsorbate–surface systems which cover an E_{int} of over 1.6 eV. Discussion of the performance of these methods can be found in the main text and we describe the computational details here.

The same k-point grids were used as those in Table 23. The GGA and meta-GGA calculations were performed with a 1000 eV energy cutoff together with small core (`Ti_sv` and `Mg_sv`) PAW potentials together with hard C, H, N and O PAW potentials. The hybrid DFT calculations were performed with the `Ti_pv` and `Mg_pv` PAW potentials with standard C, H, N and O PAW potentials with an energy cutoff of 600 eV. A correction for errors (typically less than 10 meV) in the PAW potential was calculated at the GGA (PBE-TS/HI for PBE0-TS/HI and PBE-D4 for HSE06-D4) level.

The random phase approximation (RPA) calculations were performed in VASP with an energy cutoff of 550 eV and a cutoff of 366 eV for the response function, using the same k-point grid as the DFT calculations. Using PBE orbitals, we calculated RPA and RPA+rSE [57] energies for MgO(001) in a (4×4) supercell with a 2×2×1 k-point mesh. In Table 4, we show that the RPA adsorption energy E_{ads} for CO on MgO(001) changes by only 4 meV when compared to a

TABLE 3: Comparison of E_{int} (in meV) from a set of DFAs against autoSKZCAM estimates.

	CH_4 on MgO(001)	C_2H_6 on MgO(001)	CO on MgO(001)	Physisorbed CO_2 on MgO(001)	Monomer H_2O on MgO(001)	Parallel N_2O on MgO(001)	NH_3 on MgO(001)	CH_4 on TiO_2 rutile(110)	Tilted CO_2 on TiO_2 rutile(110)	H_2O on TiO_2 rutile(110)	CH_3OH on TiO_2 rutile(110)	H_2O on TiO_2 anatase(101)	NH_3 on TiO_2 anatase(101)
autoSKZCAM	-122	-175	-207	-308	-703	-256	-657	-269	-493	-1310	-1634	-1208	-1377
RPA+rSE	-141	-200	-294	-328	-689	-269	-698	-	-	-	-	-	-
RPA	-96	-137	-98	-236	-614	-204	-630	-	-	-	-	-	-
HSE06-D4	-162	-233	-252	-319	-728	-245	-692	-295	-496	-1397	-1718	-1252	-1528
PBE0-TS/HI	-169	-262	-245	-289	-719	-231	-686	-333	-489	-1417	-1784	-1254	-1528
r^{2}SCAN-D4	-173	-244	-296	-380	-784	-294	-734	-306	-528	-1429	-1743	-1303	-1527
SCAN-rVV10	-182	-257	-323	-427	-823	-329	-761	-323	-573	-1492	-1821	-1363	-1576
rev-vdW-DF2	-144	-213	-272	-299	-672	-246	-665	-273	-462	-1286	-1607	-1163	-1402
PBE-MBD/FI	-121	-192	-325	-319	-689	-258	-687	-291	-472	-1289	-1608	-1183	-1414
PBE-D3	-257	-375	-320	-334	-770	-314	-764	-357	-439	-1280	-1611	-1176	-1441

3×3×1 k-point mesh. We used the `GW` variants of the `Mg_sv`, `C`, `H`, `N` and `O` PAW potentials and employed VASP's low-scaling implementation [58] with a plane-wave cutoff of 550 eV and a 12-point frequency grid. The complete basis set limit was estimated via the built-in extrapolation technique. We show in Table 5 that the RPA correlation contribution to the CO on MgO(001) E_{ads} changes by less than 10 meV for finite energy cutoffs (prior to extrapolation). The above convergence tests were performed on E_{ads} (using revPBE-D4 geometries) rather than E_{int} to enable comparison to previous literature, as discussed below.

TABLE 4: Convergence of the RPA energy contributions: exact exchange (EXX), RPA correlation (RPAc) and total RPA, to the adsorption energy E_{ads} (in meV) of CO on MgO(001) as a function of k-point grid size. These tests were performed with a smaller energy cutoff of 414 eV.

	EXX	RPAc	RPA
$2 \times 2 \times 1$	283	-362	-79
$3 \times 3 \times 1$	282	-365	-83

TABLE 5: Convergence of the RPA correlation (RPAc) contribution to the adsorption energy E_{ads} of CO on MgO(001) as a function of the energy cutoff for the response function (to evaluate RPAc). In VASP, the RPAc energy cutoff is normally $\frac{2}{3}$ of the standard energy cutoff (of 550 eV) and extrapolated to the infinite (∞) basis set limit from smaller energy cutoffs.

RPAc energy cutoff (eV)	RPAc E_{ads} (meV)
∞	-373
366	-376
349	-376
332	-376
316	-376
301	-376
287	-377
273	-377
260	-378

In Table 6, we compare our RPA estimates of the adsorption energy E_{ads} for CO and H_2O on MgO(001) against estimates by Bajdich *et al.* [59]. The agreement is to within 20 meV for both systems, confirming the validity and reproducibility of our chosen RPA settings.

TABLE 6: Comparison of the adsorption energy E_{ads} in meV for CO and H_2O on MgO(001) computed in this work to the work of Bajdich *et al.* [59].

	This work	Bajdich *et al.* [59]
CO	-92	-72
H_2O	-479	-492

Part II: Supporting data

3. THE APPROACH TO CALCULATING THE ADSORPTION ENTHALPY

The adsorption enthalpy H_{ads} is the central quantity in surface chemistry. It represents the enthalpy released when a molecule binds to a surface, giving a physical description for the strength of this binding. Adsorption and desorption represents primary processes within any chemical reaction happening on a surface and as such, H_{ads} is a key quantity that can control the reaction rate, as empirically shown by the Sabatier principle [60].

The standard path towards computing H_{ads} starts from E_{ads} – the adsorption energy under static conditions (i.e., zero temperature and pressure) – and adds contributions for the zero-point vibrational (ZPV) energies E_{ZPV} and vibrational temperature contributions E_{T} alongside an RT term for the work done due to pressure [61]:

$$H_{\text{ads}} = E_{\text{ads}} + E_{\text{ZPV}} + E_{\text{T}} - RT. \tag{1}$$

E_{ads} can be obtained from the total energy calculations from e.g., standard correlated wave-function theory (cWFT) and DFT calculations. It is defined as the difference between the energy of the adsorbate–surface complex and the energies of the gas-phase molecule (M) and the pristine surface (S), all in their equilibrium geometries. E_{ZPV} and E_{T} can be obtained from vibrational energies by considering a Taylor expansion of the potential energy surface (PES) around the energetically most stable equilibrium structure. This approach assumes that the molecules are localised within a potential well to one specific adsorption site. More challenging cases may require incorporating anharmonic effects, models that sample more adsorption sites or to perform global molecular dynamics to obtain ensemble averages [62]: $H_{\text{ads}} = \langle H_{\text{MS}} \rangle - \langle H_{\text{M}} \rangle - \langle H_{\text{S}} \rangle$. However, for most surface phenomena, these effects are not expected to contribute significantly to the adsorption enthalpy, especially at lower temperatures.

Within this work, we make a further breakdown of E_{ads} into a contribution from the interaction energy E_{int} and the geometric relaxation energy E_{rlx}:

$$E_{\text{ads}} = E_{\text{int}} + E_{\text{rlx}}. \tag{2}$$

The former aims to obtain only the interaction between the molecule and surface and is defined as the difference between the energy of the adsorbate–surface complex against the energies of the molecule (M//MS) and surface (M//S) constrained to their geometry in the complex. The latter accounts for the geometric relaxation of the molecule and surface in the complex from their respective equilibrium positions:

$$E_{\text{rlx}} = E_{\text{M//MS}} - E_{\text{M}} + E_{\text{S//MS}} - E_{\text{S}} \tag{3}$$

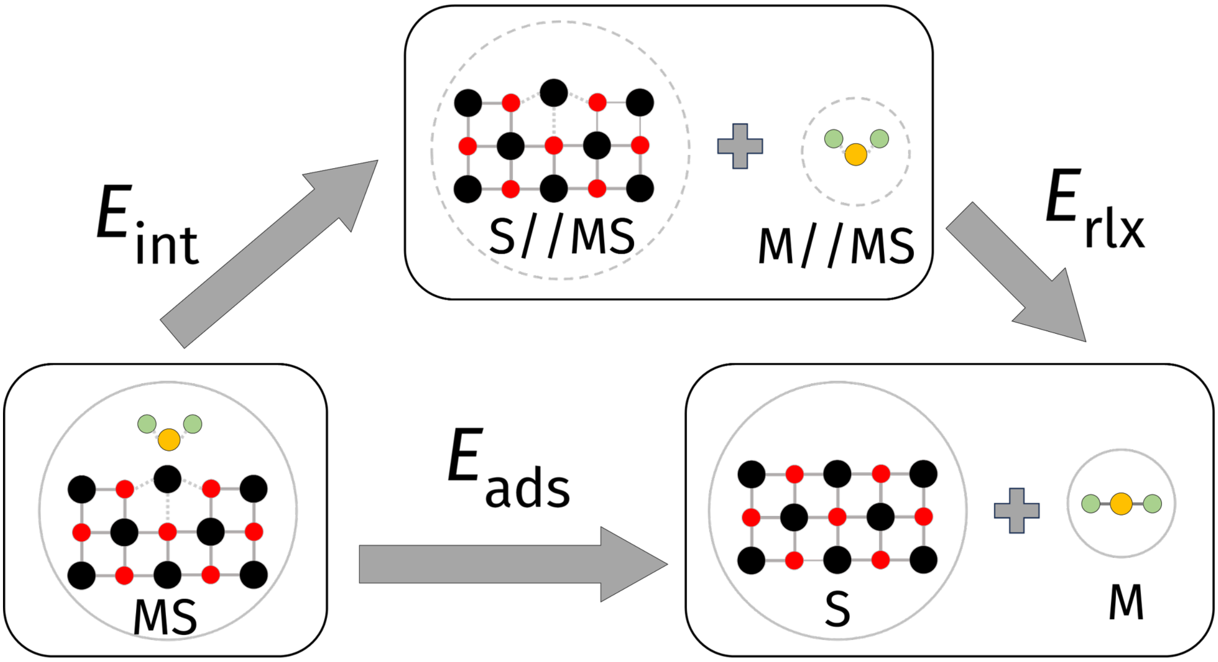

FIG. 3: The physical description of the adsorption energy E_{ads} in terms of the total energy of the adsorbate–surface complex (MS), pristine surface (S) and gas-phase molecule (M) in their equilibrium positions. This can be further broken down in a thermodynamic cycle into an interaction energy E_{int} contribution, defined as the energetic difference between MS against M and S fixed to their geometries in MS (i.e., M//MS and S//MS respectively), followed by a relaxation term E_{rlx} bring these two systems into their equilibrium geometries. The circles represent a single system/calculation, with a dashed-line circle indicating a geometry fixed to that found in the adsorbate–surface complex while a line circle indicates an equilibrium geometry.

In the subsequent sections, we will discuss how these individual terms are obtained in the autoSKZCAM framework, leading to the results in Sections 1 and 2.

4. THE ADSORBATE–SURFACE COMPLEXES STUDIED IN THIS WORK

Within this work, we have studied 19 adsorbate–surface systems in total, considering several adsorption configurations (29 in total) to obtain new insights into their binding mechanism as shown in Fig. 4. The systems consists of several molecular adsorbates of technological relevance (CO, NO, N_2O, NH_3, H_2O, CO_2, CH_3OH, CH_4, C_2H_6 and C_6H_6). The adsorption of these molecules have been considered on the MgO(001) surface and on both the TiO_2 anatase(101) and rutile(110) surfaces, the prototypical metal-oxide surfaces, all with important technological

applications. For example TiO_2 has been under heavy investigation for the photocatalytic conversion of H_2O to hydrogen [63, 64], while MgO is being investigated to be used as an adsorbent of harmful CO_2 [26] and NO_x [65] gases. These systems have been chosen because high quality experimental estimates [3] exist for their adsorption enthalpy, with open questions on their adsorption mechanism. Some of these systems have also been well-studied with computational simulations, namely density functional theory (DFT), and only a handful have been studied with methods from correlated wave-function theory (cWFT). There are often discrepancies in the predicted binding mechanism (see Section 1, which we resolve with new predictions from the autoSKZCAM framework).

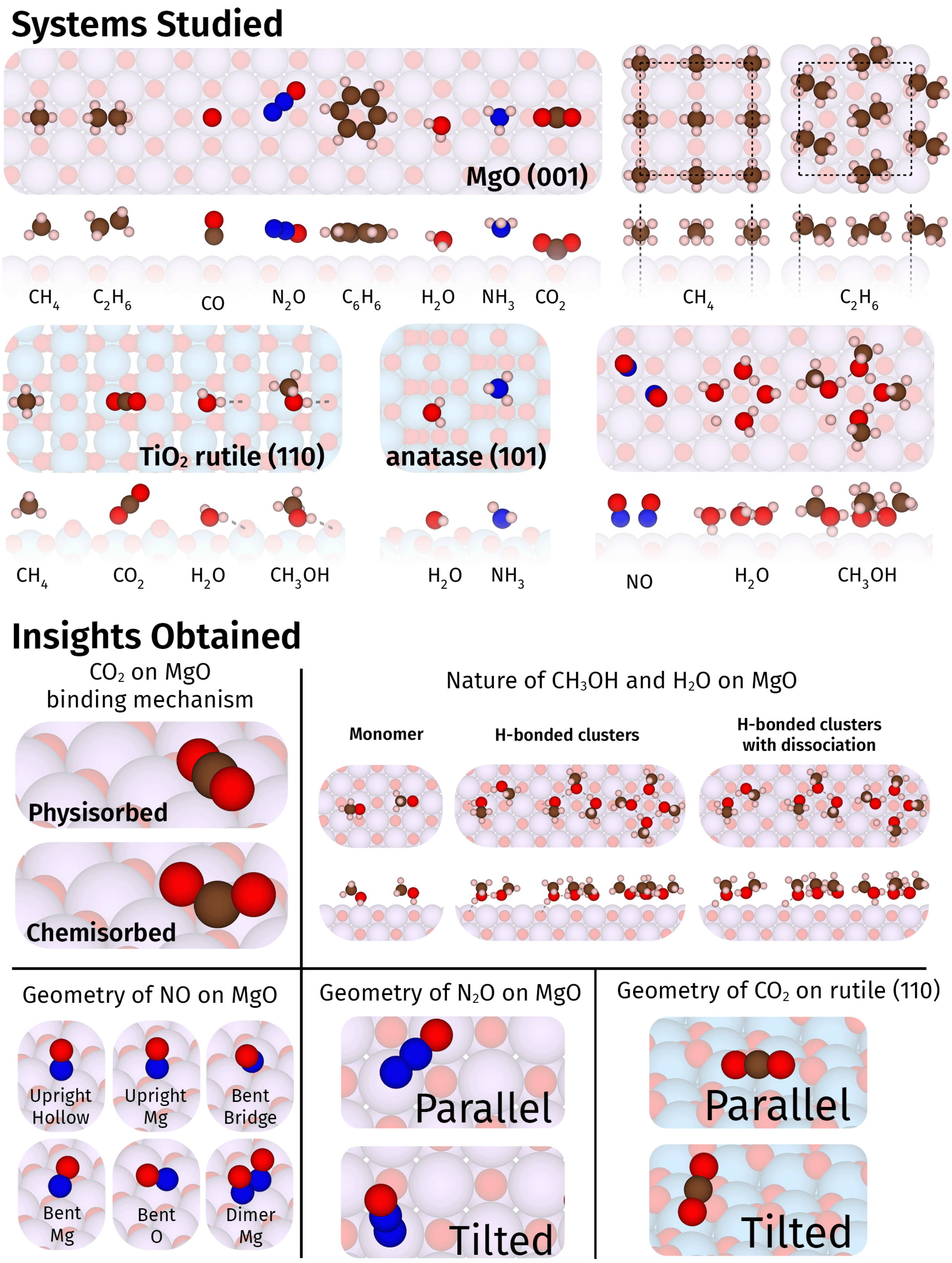

FIG. 4: The adsorbate–surface systems that we study within this work. There are 19 adsorbate–surface systems in total. For several of these systems, we have examined multiple competing adsorption configurations to reveal new atomic-level insights on how the molecules bind onto the surface.

5. COMPUTATIONAL DETAILS FOR CORRELATED WAVE-FUNCTION THEORY

The ability to reach an accurate E_{int} and ultimately H_{ads} value which agrees with experiments rests upon employing methods from correlated wave-function theory (cWFT). Specifically, we make use of two levels of theory: second-order Møller-Plesset perturbation theory (MP2) [66] and coupled cluster theory with single, double, and perturbative triple excitations [CCSD(T)] [67]. We leverage the developments within two two efficient quantum chemistry codes, ORCA 5.0.3 [68] and MRCC 2023 [69], to perform our cWFT calculations. Here, the MP2 calculations were performed with the ORCA program using the resolution-of-identity approximation. We leverage the developments in efficient local approximations to CCSD(T) – specifically the local natural orbital (LNO) [70, 71] approximation in MRCC and the domain-based local pair natural orbital (DLPNO) [72–75] approximation in ORCA. The former was used for the majority of CCSD(T) calculations while the latter was used for treating the open-shell NO monomers adsorbed on MgO. In addition, while all other systems started from a restricted Hartree-Fock (HF) reference determinant, the open-shell NO monomers on MgO(001) used an unrestricted Hartree-Fock determinant (of doublet multiplicity) for the adsorbate and adsorbate–slab complex.

We have used the Dunning family [76] of correlation consistent basis sets, where aug-cc-pVXZ are used on the non-metal (C, H, N, O) atoms, with X representing its size in terms of double (DZ), triple (TZ) or quadruple (QZ) zeta. For the metal cations (Mg and Ti), we do not include augmentation and use either the cc-pVXZ or cc-pwCVXZ basis sets alongside an associated treatment of correlation from semicore electrons on the metal cations. The former treats only valence (i.e., $3s^2$ or $3d^2 4s^2$) electrons while the latter includes weighted core-valence basis functions [77] to incorporate sub-valence s and p electron contributions to the electron correlation treatment [78]. The combinations that we specifically compute for E_{int} will be shortened to: aVDZ, aV(DZ/TZ), aV(TZ/QZ), awCVDZ, awCV(DZ/TZ), awCV(TZ/QZ). Here, the 'a' in front indicates that there is an augmentation treatment (only on the non-metal atoms), while the inclusion of wC indicates the use of core-valence basis sets on the metal atoms. Those with (DZ/TZ) or (TZ/QZ) involve a two-point complete basis set (CBS) extrapolation, using parameters taken from Neese and Valeev [79], for the enclosed pair of basis functions.

We use the def2-QZVPP-RI-JK auxiliary basis function for density-fitting/resolution-of-identity Hartree–Fock (HF) computations, and the RI auxiliary basis sets from Weigend [80, 81] corresponding to the AO basis sets for subsequent cWFT calculations. For the metal atoms, where RI basis functions were not available, we generated automatic auxiliary basis functions

using the approach of Stoychev *et al.* [82, 83]. The interaction energy calculations all employed counterpoise corrections to overcome basis set superposition errors. We used `TightPNO` settings for the DLPNO-CCSD(T) treatment in ORCA and `tight` LNO thresholds together with setting `bpedo=0.99999` for improved correlation energy capture in MRCC. The corresponding DLPNO-MP2 and LMP2 estimates with the same settings were used when calculating the ΔCC quantity. Within LNO-CCSD(T), the LMP2 energy comes out naturally as the MP2 amplitudes are used to construct local natural orbitals and (local) truncation errors are corrected at the MP2 level [71]. On the other hand, DLPNO-MP2 is evaluated in a separate calculation from DLPNO-CCSD(T).

6. INTERACTION ENERGIES COMPUTED WITH THE SKZCAM PROTOCOL

The interaction energy E_{int} forms the majority of the contribution towards H_{ads} and it can be challenging for DFT to predict correctly (see Fig. 4 of the main text). The SKZCAM protocol was developed in Refs. 84 and 85 to reach a CCSD(T)-quality E_{int} at low cost for adsorbate–surface systems involving ionic materials. There are four major steps in the SKZCAM protocol (illustrated in Fig. 5), starting with (a) constructing an electrostatic embedding environment, (b) generating the (embedded) clusters and (c) extrapolating E_{int} to the bulk limit at the lower-level second-order Møller-Plesset perturbation theory before (d) elevating E_{int} to the CCSD(T) level with a ΔCC contribution through mechanical embedding. We will describe each of these steps in the next few sub-sections, providing tables with the contributions towards the final E_{int}.

A. Generating a systematic series of clusters within electrostatic embedding

The SKZCAM protocol models the surface through an electrostatic embedding scheme [86], where a (quantum) cluster that has been cleaved out of the surface is coupled to an environment that represents the interactions arising from the atoms outside the cluster. This environment consists of point charges placed at the positions of the metal cations and oxygen anions, taking formal values of +4 and +2 for Ti and Mg respectively, while taking a value of -2 for O. We have used the py-ChemShell 2020 package [87] to generate the embedding environment, placing point charges within a hemisphere (of radius `radius_cluster=50` Å for TiO_2 [both rutile(110) and anatase(101)] and 60 Å for the MgO(001) surface, respectively) around the adsorbate molecule, with a further set of (fitting) point charges placed (`bq_layer=6` Å) on the

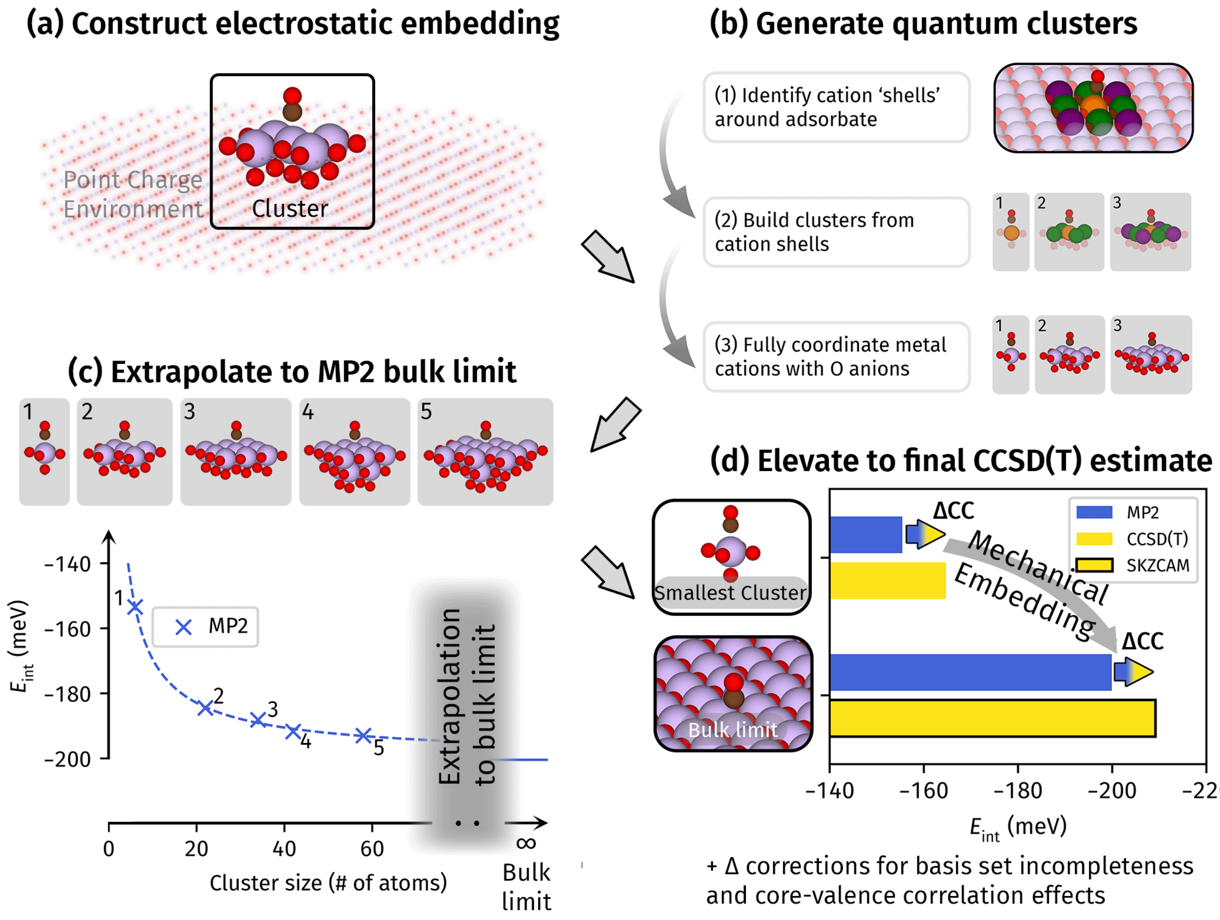

FIG. 5: Schematic of the SKZCAM protocol to calculate E_{int}. It starts by (a) generating the electrostatic embedding environment, followed by (b) creating the series of systematically converging clusters. From these clusters, the (c) MP2 bulk limit can be obtained through extrapolation. Finally, we reach the CCSD(T) estimate by adding a ΔCC correction (from the smallest cluster) onto this MP2 bulk limit.

outer edge to reproduce the Madelung potential within (`radius_active`=)40Å the adsorbate. Subsequently, after cleaving the quantum cluster, capped effective core potentials (ECP) are placed on positive (metal cation) point charges within a radius of `cutoff_boundary`=6Å and 4Å for the TiO_2 and 60 Å for MgO surfaces, respectively, to prevent spurious charge leakage.

With the electrostatic embedding environment created, the key remaining question is how to design the quantum cluster with particular attention required on controlling its size. High-level methods from cWFT, such as CCSD(T), can quickly become intractable for larger clusters while small clusters are not typically converged towards the bulk (infinite size) limit. The SKZCAM protocol provides a set of rubrics - hence its description as a 'protocol' - to automatically gen-

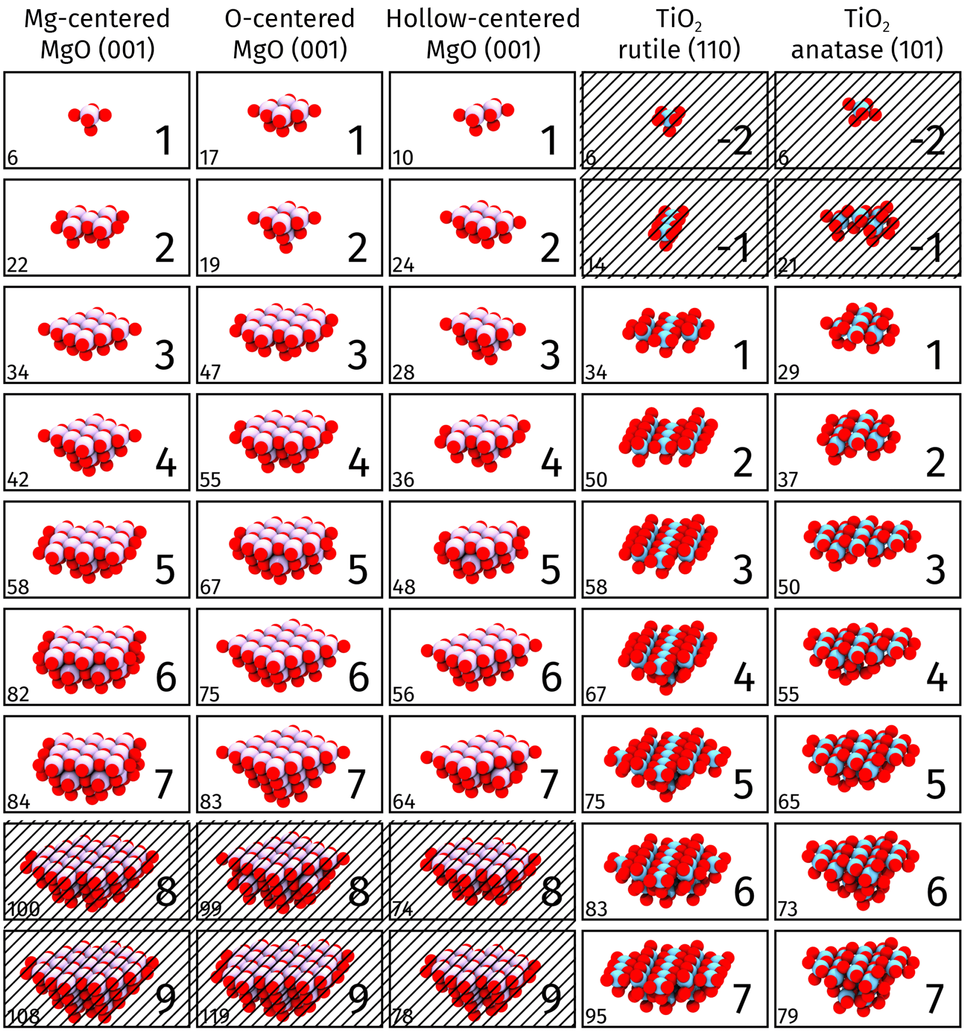

FIG. 6: The series of clusters generated by the SKZCAM protocol for the systems studied within this work. We have generated the first 9 clusters from the SKZCAM protocol and shade in hatched lines those which we do not study within this work, as explained within the text.

erate a set of small clusters [as shown in part (b) of Fig. 5]. It involves a simple two-step process, whereby cation metal shells (from e.g., a radial distribution function around the adsorbate molecule) are used to progressively build up the metal cations in clusters of growing size. This is followed by fully coordinating the metal cations with O anions to complete each cluster. The first step is intuitive since it is expected that atoms closer to the adsorbate contribute most significantly to E_{int} while we have found that convergence of properties such as vacancy formation energies [84] and adsorption energies [85] become significantly faster when the metal cations are fully coordinated by O anions in the second step to prevent any dangling bonds on the metal cations.

In Fig. 6, we visualise the set of clusters generated by the SKZCAM protocol for the MgO(001),

TiO_2 rutile(110) and anatase(101) surfaces respectively. Based on the rubrics explained above, the generated clusters will be adapted to be efficient and centered around each specific adsorbate–surface system. For the MgO(001) surface, there are three different sets of clusters which will be generated based on the adsorption site - an 'Mg-centered', 'O-centered' or 'Hollow-centered' cluster. The adsorbate–surface systems for the rutile(110) and anatase(101) surfaces have their adsorbates all centred on the Ti-site, so only one set of unique clusters ended up being generated for each of the system.

We specifically show the first 9 clusters generated by the SKZCAM protocol (although clusters up to arbitrary size can be created). We find that only the first 7 clusters are required to reach accurate estimates of E_{int}, so only the first 7 clusters were used for the MgO(001) surface as described in the subsequent sections. For the clusters used in the TiO_2 rutile(110) and anatase(101) surfaces, we find that the first two clusters (dubbed '-2' and '-1' clusters) were too small to provide sensible E_{int} values, instead starting the SKZCAM protocol from the third cluster onwards (dubbed '1', '2', '3',. . . ,'7')

B. Extrapolating towards the bulk limit with the series of clusters

The series of clusters generated by the SKZCAM protocol achieves several qualities that can be exploited to reach the CCSD(T) bulk limit accurately and efficient, which we discuss in this section and the next. One of the key properties is that there is a smooth and fast convergence of E_{int} with cluster size (as a function of the number of atoms N in the cluster). For the MgO surfaces, we use this smooth convergence to extrapolate towards the bulk limit $E_{\text{int}}^{\text{bulk}}$, using a formula of the form:

$$E_{\text{int}}(N) = E_{\text{int}}^{\text{bulk}} + \frac{A}{N}, \tag{4}$$

where A and $E_{\text{int}}^{\text{bulk}}$ can be obtained by fitting E_{int} to the series of clusters. This formula was inspired by the N^{-1} scaling of finite-effects observed in cWFT methods [88] and the dependence of E_{int} on a pairwise dispersive additive interaction is N^{-2}, which has been verified for H_2O adsorption on the 2D hBN surface [89]. Regardless of the formula, given the systematic convergence of E_{int} to $E_{\text{int}}^{\text{bulk}}$, we expect to reach the correct extrapolated value once sufficient number of clusters are included within the extrapolation. As a rule of thumb, we find that fits involving the first 5 clusters from the SKZCAM protocol are sufficient at reaching a converged estimate to within 5 meV, which we observe for most of the systems (see Table 7). The largest cluster may involve sizes up to 70 atoms, and while this size may be too large for CCSD(T), it can be affordably

tackled with MP2 at the complete basis set (CBS) limit using a two-point extrapolation with the double-zeta (DZ) and triple-zeta (TZ) basis sets. Furthermore, we can estimate the error on this fit by using MP2 with the smaller DZ basis set to observe how $E_{\text{int}}^{\text{bulk}}$ changes as more clusters are included. We have specifically estimated the error of our CBS(DZ/TZ) estimate of $E_{\text{int}}^{\text{bulk}}$ with the fifth cluster from the SKZCAM protocol by finding its maximum deviation at the DZ level when compared to incorporating 6 or 7 clusters in the fit.

For both the TiO_2 surfaces, we find that the convergence is not as smooth as with the MgO surface, with E_{int} oscillating as a function of cluster size. Such behaviour is reminiscent of the odd-even oscillations common to TiO_2 studies [90] and what we observed for oxygen vacancy formation energies [84]. For these systems, we simply set $E_{\text{int}}^{\text{bulk}}$ to be the E_{int} value from the fifth cluster, and set the error bar to the maximum deviation observed with respect to larger clusters with the DZ basis set. We find errors that are typically below 20 meV for most of the studied TiO_2 adsorbate–surface systems, with only the H_2O and CH_3OH molecules on rutile(110) leading to slightly larger errors that are still within 40 meV.

TABLE 7: E_{int} (in meV) of the clusters generated by the SKZCAM protocol for the 19 adsorbate–surface systems and their studied adsorption configurations. The type of clusters used is given within the brackets and the corresponding size for each cluster is provided in Figure 6.

CH_4 on MgO(001) (Mg-centered)	1	2	3	4	5	6	7
E_{int} MP2 awCVDZ	-25	-55	-62	-65	-69	-72	-73
$E_{\text{int}}^{\text{bulk}}$ MP2 awCVDZ	-	-	-	-70	-71	-72	-73
E_{int} MP2 CBS(awCVDZ/awCVTZ)	-71	-96	-101	-103	-105	-	-
$E_{\text{int}}^{\text{bulk}}$ MP2 CBS(awCVDZ/awCVTZ)	-	-	-	-107	-108	-	-
E_{int} MP2 CBS(awCVTZ/awCVQZ)	-68	-95	-99	-	-	-	-
E_{int} LMP2 CBS(awCVDZ/awCVTZ)	-70	-91	-93	-	-	-	-
E_{int} LNO-CCSD(T) CBS(awCVDZ/awCVTZ)	-86	-107	-109	-	-	-	-
**Monolayer CH_4 on MgO(001) (Mg-centered)**	**1**	**2**	**3**	**4**	**5**	**6**	**7**
E_{int} MP2 awCVDZ	-24	-54	-62	-64	-68	-71	-73
$E_{\text{int}}^{\text{bulk}}$ MP2 awCVDZ	-	-	-	-69	-70	-72	-73
E_{int} MP2 CBS(awCVDZ/awCVTZ)	-70	-95	-100	-102	-104	-	-
$E_{\text{int}}^{\text{bulk}}$ MP2 CBS(awCVDZ/awCVTZ)	-	-	-	-106	-107	-	-

Continued on next page

TABLE 7: (continued)

E_{int} MP2 CBS(awCVTZ/awCVQZ)	-67	-94	-98	-	-	-	-
E_{int} LMP2 CBS(awCVDZ/awCVTZ)	-69	-90	-94	-	-	-	-
E_{int} LNO-CCSD(T) CBS(awCVDZ/awCVTZ)	-85	-106	-110	-	-	-	-
C_2H_6 on MgO(001) (Mg-centered)	1	2	3	4	5	6	7
E_{int} MP2 awCVDZ	-30	-79	-93	-97	-104	-108	-110
E_{int}^{bulk} MP2 awCVDZ	-	-	-	-105	-107	-109	-110
E_{int} MP2 CBS(awCVDZ/awCVTZ)	-90	-136	-146	-149	-153	-	-
E_{int}^{bulk} MP2 CBS(awCVDZ/awCVTZ)	-	-	-	-157	-158	-	-
E_{int} MP2 CBS(awCVTZ/awCVQZ)	-87	-133	-141	-	-	-	-
E_{int} LMP2 CBS(awCVDZ/awCVTZ)	-92	-131	-140	-	-	-	-
E_{int} LNO-CCSD(T) CBS(awCVDZ/awCVTZ)	-112	-151	-160	-	-	-	-
Monolayer C_2H_6 on MgO(001) (Mg-centered)	1	2	3	4	5	6	7
E_{int} MP2 awCVDZ	-21	-71	-86	-89	-96	-101	-102
E_{int}^{bulk} MP2 awCVDZ	-	-	-	-98	-100	-102	-103
E_{int} MP2 CBS(awCVDZ/awCVTZ)	-73	-121	-131	-134	-139	-	-
E_{int}^{bulk} MP2 CBS(awCVDZ/awCVTZ)	-	-	-	-142	-144	-	-
E_{int} MP2 CBS(awCVTZ/awCVQZ)	-71	-118	-127	-	-	-	-
E_{int} LMP2 CBS(awCVDZ/awCVTZ)	-72	-115	-124	-	-	-	-
E_{int} LNO-CCSD(T) CBS(awCVDZ/awCVTZ)	-91	-135	-144	-	-	-	-
CO on MgO(001) (Mg-centered)	1	2	3	4	5	6	7
E_{int} MP2 awCVDZ	-47	-100	-111	-117	-122	-127	-128
E_{int}^{bulk} MP2 awCVDZ	-	-	-	-125	-127	-128	-129
E_{int} MP2 CBS(awCVDZ/awCVTZ)	-155	-184	-192	-195	-197	-	-
E_{int}^{bulk} MP2 CBS(awCVDZ/awCVTZ)	-	-	-	-199	-200	-	-
E_{int} MP2 CBS(awCVTZ/awCVQZ)	-155	-186	-189	-	-	-	-
E_{int} LMP2 CBS(awCVDZ/awCVTZ)	-153	-183	-185	-	-	-	-
E_{int} LNO-CCSD(T) CBS(awCVDZ/awCVTZ)	-160	-190	-194	-	-	-	-

Continued on next page

TABLE 7: (continued)

C_6H_6 on MgO(001) (O-centered)	1	2	3	4	5	6	7
E_{int} MP2 awCVDZ	-197	-203	-305	-313	-322	-330	-335
$E_{\text{int}}^{\text{bulk}}$ MP2 awCVDZ	-	-	-	-367	-367	-368	-369
E_{int} MP2 CBS(awCVDZ/awCVTZ)	-338	-344	-414	-422	-428	-	-
$E_{\text{int}}^{\text{bulk}}$ MP2 CBS(awCVDZ/awCVTZ)	-	-	-	-460	-460	-	-
E_{int} MP2 CBS(awCVTZ/awCVQZ)	-354	-359	-416	-	-	-	-
E_{int} LMP2 CBS(awCVDZ/awCVTZ)	-335	-340	-404	-	-	-	-
E_{int} LNO-CCSD(T) CBS(awCVDZ/awCVTZ)	-311	-316	-379	-	-	-	-
**Parallel N_2O on MgO(001) (Hollow-centered)**	**1**	**2**	**3**	**4**	**5**	**6**	**7**
E_{int} MP2 awCVDZ	-143	-175	-179	-185	-188	-193	-195
$E_{\text{int}}^{\text{bulk}}$ MP2 awCVDZ	-	-	-	-200	-200	-201	-202
E_{int} MP2 CBS(awCVDZ/awCVTZ)	-206	-229	-232	-238	-242	-	-
$E_{\text{int}}^{\text{bulk}}$ MP2 CBS(awCVDZ/awCVTZ)	-	-	-	-248	-249	-	-
E_{int} MP2 CBS(awCVTZ/awCVQZ)	-207	-231	-230	-	-	-	-
E_{int} LMP2 CBS(awCVDZ/awCVTZ)	-203	-225	-226	-	-	-	-
E_{int} LNO-CCSD(T) CBS(awCVDZ/awCVTZ)	-209	-231	-233	-	-	-	-
**Tilted N_2O on MgO(001) (Mg-centered)**	**1**	**2**	**3**	**4**	**5**	**6**	**7**
E_{int} MP2 awCVDZ	-44	-98	-111	-116	-122	-126	-129
$E_{\text{int}}^{\text{bulk}}$ MP2 awCVDZ	-	-	-	-125	-127	-128	-130
E_{int} MP2 CBS(awCVDZ/awCVTZ)	-122	-159	-168	-172	-175	-	-
$E_{\text{int}}^{\text{bulk}}$ MP2 CBS(awCVDZ/awCVTZ)	-	-	-	-178	-179	-	-
E_{int} MP2 CBS(awCVTZ/awCVQZ)	-129	-165	-171	-	-	-	-
E_{int} LMP2 CBS(awCVDZ/awCVTZ)	-120	-155	-159	-	-	-	-
E_{int} LNO-CCSD(T) CBS(awCVDZ/awCVTZ)	-104	-139	-144	-	-	-	-
**Vertical-Hollow NO on MgO(001) (Hollow-centered)**	**1**	**2**	**3**	**4**	**5**	**6**	**7**
E_{int} MP2 aVDZ	-159	-177	-183	-185	-186	-185	-186
$E_{\text{int}}^{\text{bulk}}$ MP2 aVDZ	-	-	-	-194	-194	-193	-192

Continued on next page

TABLE 7: (continued)

E_{int} MP2 CBS(aVDZ/aVTZ)	-224	-236	-233	-240	-241	-	-
$E_{\text{int}}^{\text{bulk}}$ MP2 CBS(aVDZ/aVTZ)	-	-	-	-243	-244	-	-
E_{int} MP2 CBS(awCVDZ/awCVTZ)	-242	-259	-257	-	-	-	-
E_{int} MP2 CBS(aVTZ/aVQZ)	-213	-227	-225	-	-	-	-
E_{int} DLPNO-MP2 CBS(aVDZ/aVTZ)	-219	-	-	-	-	-	-
E_{int} DLPNO-CCSD(T) CBS(aVDZ/aVTZ)	71	-	-	-	-	-	-
Vertical-Mg NO on MgO(001) (Mg-centered)	1	2	3	4	5	6	7
E_{int} MP2 aVDZ	135	94	91	86	82	80	77
$E_{\text{int}}^{\text{bulk}}$ MP2 aVDZ	-	-	-	79	78	78	77
E_{int} MP2 CBS(aVDZ/aVTZ)	65	35	33	33	30	-	-
$E_{\text{int}}^{\text{bulk}}$ MP2 CBS(aVDZ/aVTZ)	-	-	-	26	26	-	-
E_{int} MP2 CBS(awCVDZ/awCVTZ)	43	9	5	-	-	-	-
E_{int} MP2 CBS(aVTZ/aVQZ)	67	42	41	-	-	-	-
E_{int} DLPNO-MP2 CBS(aVDZ/aVTZ)	64	-	-	-	-	-	-
E_{int} DLPNO-CCSD(T) CBS(aVDZ/aVTZ)	-4	-	-	-	-	-	-
Bent-Bridge NO on MgO(001) (Mg-centered)	1	2	3	4	5	6	7
E_{int} MP2 aVDZ	-460	-486	-494	-503	-505	-507	-510
$E_{\text{int}}^{\text{bulk}}$ MP2 aVDZ	-	-	-	-503	-505	-507	-508
E_{int} MP2 CBS(aVDZ/aVTZ)	-620	-649	-654	-656	-658	-	-
$E_{\text{int}}^{\text{bulk}}$ MP2 CBS(aVDZ/aVTZ)	-	-	-	-661	-661	-	-
E_{int} MP2 CBS(awCVDZ/awCVTZ)	-639	-675	-682	-	-	-	-
E_{int} MP2 CBS(aVTZ/aVQZ)	-618	-654	-658	-	-	-	-
E_{int} DLPNO-MP2 CBS(aVDZ/aVTZ)	-620	-	-	-	-	-	-
E_{int} DLPNO-CCSD(T) CBS(aVDZ/aVTZ)	6	-	-	-	-	-	-
Bent-Mg NO on MgO(001) (Mg-centered)	1	2	3	4	5	6	7
E_{int} MP2 aVDZ	52	9	5	2	-2	-4	-7
$E_{\text{int}}^{\text{bulk}}$ MP2 aVDZ	-	-	-	-6	-7	-7	-8
E_{int} MP2 CBS(aVDZ/aVTZ)	-15	-43	-46	-47	-50	-	-

Continued on next page

TABLE 7: (continued)

$E_{\text{int}}^{\text{bulk}}$ MP2 CBS(aVDZ/aVTZ)	-	-	-	-53	-53	-	-
E_{int} MP2 CBS(awCVDZ/awCVTZ)	-37	-70	-75	-	-	-	-
E_{int} MP2 CBS(aVTZ/aVQZ)	-17	-42	-44	-	-	-	-
E_{int} DLPNO-MP2 CBS(aVDZ/aVTZ)	-14	-	-	-	-	-	-
E_{int} DLPNO-CCSD(T) CBS(aVDZ/aVTZ)	-62	-	-	-	-	-	-
Bent-O NO on MgO(001) (O-centered)	1	2	3	4	5	6	7
E_{int} MP2 aVDZ	-496	-500	-502	-502	-504	-503	-503
$E_{\text{int}}^{\text{bulk}}$ MP2 aVDZ	-	-	-	-505	-506	-505	-505
E_{int} MP2 CBS(aVDZ/aVTZ)	-640	-641	-643	-645	-646	-	-
$E_{\text{int}}^{\text{bulk}}$ MP2 CBS(aVDZ/aVTZ)	-	-	-	-646	-647	-	-
E_{int} MP2 CBS(awCVDZ/awCVTZ)	-649	-656	-661	-	-	-	-
E_{int} MP2 CBS(aVTZ/aVQZ)	-639	-638	-648	-	-	-	-
E_{int} DLPNO-MP2 CBS(aVDZ/aVTZ)	-630	-	-	-	-	-	-
E_{int} DLPNO-CCSD(T) CBS(aVDZ/aVTZ)	29	-	-	-	-	-	-
Dimer NO on MgO(001) (Hollow-centered)	1	2	3	4	5	6	7
E_{int} MP2 awCVDZ	-138	-172	-177	-184	-188	-191	-193
$E_{\text{int}}^{\text{bulk}}$ MP2 awCVDZ	-	-	-	-200	-200	-201	-202
E_{int} MP2 CBS(awCVDZ/awCVTZ)	-222	-244	-246	-251	-253	-	-
$E_{\text{int}}^{\text{bulk}}$ MP2 CBS(awCVDZ/awCVTZ)	-	-	-	-261	-261	-	-
E_{int} MP2 CBS(awCVTZ/awCVQZ)	-226	-246	-246	-	-	-	-
E_{int} LMP2 CBS(awCVDZ/awCVTZ)	-217	-239	-	-	-	-	-
E_{int} LNO-CCSD(T) CBS(awCVDZ/awCVTZ)	-199	-222	-	-	-	-	-
Monomer H_2O on MgO(001) (Mg-centered)	1	2	3	4	5	6	7
E_{int} MP2 awCVDZ	-514	-542	-546	-552	-556	-559	-559
$E_{\text{int}}^{\text{bulk}}$ MP2 awCVDZ	-	-	-	-555	-557	-558	-559
E_{int} MP2 CBS(awCVDZ/awCVTZ)	-641	-667	-671	-671	-674	-	-
$E_{\text{int}}^{\text{bulk}}$ MP2 CBS(awCVDZ/awCVTZ)	-	-	-	-677	-677	-	-
E_{int} MP2 CBS(awCVTZ/awCVQZ)	-641	-670	-668	-	-	-	-

Continued on next page

TABLE 7: (continued)

E_{int} LMP2 CBS(awCVDZ/awCVTZ)	-639	-663	-664	-	-	-	-
E_{int} LNO-CCSD(T) CBS(awCVDZ/awCVTZ)	-665	-688	-689	-	-	-	-
Tetramer H_2O on MgO(001) (O-centered)	1	2	3	4	5	6	7
E_{int} MP2 awCVDZ	-296	-299	-333	-335	-339	-344	-347
E_{int}^{bulk} MP2 awCVDZ	-	-	-	-354	-354	-355	-357
E_{int} MP2 CBS(awCVDZ/awCVTZ)	-392	-394	-409	-412	-414	-	-
E_{int}^{bulk} MP2 CBS(awCVDZ/awCVTZ)	-	-	-	-420	-421	-	-
E_{int} MP2 CBS(awCVTZ/awCVQZ)	-411	-412	-418	-	-	-	-
E_{int} LMP2 CBS(awCVDZ/awCVTZ)	-390	-392	-407	-	-	-	-
E_{int} LNO-CCSD(T) CBS(awCVDZ/awCVTZ)	-417	-419	-434	-	-	-	-
Tilted CH_3OH on MgO(001) (Mg-centered)	1	2	3	4	5	6	7
E_{int} MP2 awCVDZ	-569	-603	-613	-619	-625	-630	-632
E_{int}^{bulk} MP2 awCVDZ	-	-	-	-623	-625	-628	-629
E_{int} MP2 CBS(awCVDZ/awCVTZ)	-703	-745	-754	-756	-760	-	-
E_{int}^{bulk} MP2 CBS(awCVDZ/awCVTZ)	-	-	-	-764	-765	-	-
E_{int} MP2 CBS(awCVTZ/awCVQZ)	-704	-749	-752	-	-	-	-
E_{int} LMP2 CBS(awCVDZ/awCVTZ)	-701	-739	-746	-	-	-	-
E_{int} LNO-CCSD(T) CBS(awCVDZ/awCVTZ)	-723	-759	-768	-	-	-	-
Parallel CH_3OH on MgO(001) (Mg-centered)	1	2	3	4	5	6	7
E_{int} MP2 awCVDZ	-395	-424	-423	-429	-427	-429	-428
E_{int}^{bulk} MP2 awCVDZ	-	-	-	-433	-432	-432	-432
E_{int} MP2 CBS(awCVDZ/awCVTZ)	-443	-493	-499	-496	-499	-	-
E_{int}^{bulk} MP2 CBS(awCVDZ/awCVTZ)	-	-	-	-509	-508	-	-
E_{int} MP2 CBS(awCVTZ/awCVQZ)	-426	-474	-481	-	-	-	-
E_{int} LMP2 CBS(awCVDZ/awCVTZ)	-442	-488	-491	-	-	-	-
E_{int} LNO-CCSD(T) CBS(awCVDZ/awCVTZ)	-458	-505	-507	-	-	-	-
Tetramer CH_3OH on MgO(001) (O-centered)	1	2	3	4	5	6	7

Continued on next page

TABLE 7: (continued)

E_{int} MP2 awCVDZ	-320	-323	-370	-373	-378	-386	-389
E_{int}^{bulk} MP2 awCVDZ	-	-	-	-399	-399	-401	-403
E_{int} MP2 CBS(awCVDZ/awCVTZ)	-423	-425	-451	-455	-457	-	-
E_{int}^{bulk} MP2 CBS(awCVDZ/awCVTZ)	-	-	-	-469	-469	-	-
E_{int} MP2 CBS(awCVTZ/awCVQZ)	-440	-443	-462	-	-	-	-
E_{int} LMP2 CBS(awCVDZ/awCVTZ)	-420	-422	-449	-	-	-	-
E_{int} LNO-CCSD(T) CBS(awCVDZ/awCVTZ)	-447	-449	-476	-	-	-	-
NH_3 on MgO(001) (Mg-centered)	1	2	3	4	5	6	7
E_{int} MP2 awCVDZ	-377	-449	-475	-482	-493	-500	-501
E_{int}^{bulk} MP2 awCVDZ	-	-	-	-492	-497	-500	-502
E_{int} MP2 CBS(awCVDZ/awCVTZ)	-551	-582	-602	-606	-608	-	-
E_{int}^{bulk} MP2 CBS(awCVDZ/awCVTZ)	-	-	-	-608	-610	-	-
E_{int} MP2 CBS(awCVTZ/awCVQZ)	-570	-602	-611	-	-	-	-
E_{int} LMP2 CBS(awCVDZ/awCVTZ)	-547	-580	-599	-	-	-	-
E_{int} LNO-CCSD(T) CBS(awCVDZ/awCVTZ)	-578	-610	-630	-	-	-	-
Physisorbed CO_2 on MgO(001) (Hollow-centered)	1	2	3	4	5	6	7
E_{int} MP2 awCVDZ	-147	-180	-184	-186	-188	-189	-192
E_{int}^{bulk} MP2 awCVDZ	-	-	-	-202	-201	-200	-200
E_{int} MP2 CBS(awCVDZ/awCVTZ)	-240	-264	-265	-269	-272	-	-
E_{int}^{bulk} MP2 CBS(awCVDZ/awCVTZ)	-	-	-	-280	-280	-	-
E_{int} MP2 CBS(awCVTZ/awCVQZ)	-239	-262	-261	-	-	-	-
E_{int} LMP2 CBS(awCVDZ/awCVTZ)	-237	-258	-261	-	-	-	-
E_{int} LNO-CCSD(T) CBS(awCVDZ/awCVTZ)	-267	-288	-293	-	-	-	-
Chemisorbed CO_2 on MgO(001) (O-centered)	1	2	3	4	5	6	7
E_{int} MP2 awCVDZ	-2843	-2857	-2760	-2767	-2774	-2754	-2743
E_{int}^{bulk} MP2 awCVDZ	-	-	-	-2767	-2774	-2754	-2743
E_{int} MP2 CBS(awCVDZ/awCVTZ)	-3334	-3332	-3292	-3295	-3309	-	-

Continued on next page

TABLE 7: (continued)

E_{int}^{bulk} MP2 CBS(awCVDZ/awCVTZ)	-	-	-	-3295	-3309	-	-
E_{int} MP2 CBS(awCVTZ/awCVQZ)	-3311	-3301	-3272	-	-	-	-
E_{int} LMP2 CBS(awCVDZ/awCVTZ)	-3330	-3328	-3283	-	-	-	-
E_{int} LNO-CCSD(T) CBS(awCVDZ/awCVTZ)	-3548	-3547	-3506	-	-	-	-
CH_4 on TiO_2 rutile(110)	1	2	3	4	5	6	7
E_{int} MP2 aVDZ	-219	-202	-208	-208	-211	-210	-213
E_{int}^{bulk} MP2 aVDZ	-	-	-	-208	-211	-210	-213
E_{int} MP2 CBS(aVDZ/aVTZ)	-282	-262	-267	-265	-267	-	-
E_{int}^{bulk} MP2 CBS(aVDZ/aVTZ)	-	-	-	-265	-267	-	-
E_{int} MP2 CBS(aVTZ/aVQZ)	-281	-261	-	-	-	-	-
E_{int} MP2 CBS(awCVTZ/awCVQZ)	-283	-263	-	-	-	-	-
E_{int} LMP2 CBS(aVDZ/aVTZ)	-282	-263	-	-	-	-	-
E_{int} LNO-CCSD(T) CBS(aVDZ/aVTZ)	-283	-263	-	-	-	-	-
Parallel CO_2 on TiO_2 rutile(110)	1	2	3	4	5	6	7
E_{int} MP2 aVDZ	-337	-315	-320	-323	-327	-326	-331
E_{int}^{bulk} MP2 aVDZ	-	-	-	-323	-327	-326	-331
E_{int} MP2 CBS(aVDZ/aVTZ)	-425	-401	-405	-405	-408	-	-
E_{int}^{bulk} MP2 CBS(aVDZ/aVTZ)	-	-	-	-405	-408	-	-
E_{int} MP2 CBS(aVTZ/aVQZ)	-425	-404	-	-	-	-	-
E_{int} MP2 CBS(awCVTZ/awCVQZ)	-433	-411	-	-	-	-	-
E_{int} LMP2 CBS(aVDZ/aVTZ)	-421	-398	-	-	-	-	-
E_{int} LNO-CCSD(T) CBS(aVDZ/aVTZ)	-413	-393	-	-	-	-	-
Tilted CO_2 on TiO_2 rutile(110)	1	2	3	4	5	6	7
E_{int} MP2 aVDZ	-441	-390	-401	-395	-399	-392	-396
E_{int}^{bulk} MP2 aVDZ	-	-	-	-395	-399	-392	-396
E_{int} MP2 CBS(aVDZ/aVTZ)	-521	-467	-476	-468	-470	-	-
E_{int}^{bulk} MP2 CBS(aVDZ/aVTZ)	-	-	-	-468	-470	-	-
E_{int} MP2 CBS(aVTZ/aVQZ)	-527	-475	-	-	-	-	-

Continued on next page

TABLE 7: (continued)

E_{int} MP2 CBS(awCVTZ/awCVQZ)	-519	-466	-	-	-	-	-
E_{int} LMP2 CBS(aVDZ/aVTZ)	-516	-461	-	-	-	-	-
E_{int} LNO-CCSD(T) CBS(aVDZ/aVTZ)	-539	-486	-	-	-	-	-
H_2O on TiO_2 rutile(110)	1	2	3	4	5	6	7
E_{int} MP2 aVDZ	-1256	-1141	-1161	-1139	-1146	-1113	-1119
E_{int}^{bulk} MP2 aVDZ	-	-	-	-1139	-1146	-1113	-1119
E_{int} MP2 CBS(aVDZ/aVTZ)	-1420	-1304	-1319	-1292	-1299	-	-
E_{int}^{bulk} MP2 CBS(aVDZ/aVTZ)	-	-	-	-1292	-1299	-	-
E_{int} MP2 CBS(aVTZ/aVQZ)	-1436	-1324	-	-	-	-	-
E_{int} MP2 CBS(awCVTZ/awCVQZ)	-1399	-1280	-	-	-	-	-
E_{int} LMP2 CBS(aVDZ/aVTZ)	-1418	-1301	-	-	-	-	-
E_{int} LNO-CCSD(T) CBS(aVDZ/aVTZ)	-1447	-1340	-	-	-	-	-
CH_3OH on TiO_2 rutile(110)	1	2	3	4	5	6	7
E_{int} MP2 aVDZ	-1555	-1427	-1451	-1433	-1441	-1405	-1413
E_{int}^{bulk} MP2 aVDZ	-	-	-	-1433	-1441	-1405	-1413
E_{int} MP2 CBS(aVDZ/aVTZ)	-1752	-1622	-1641	-1616	-1624	-	-
E_{int}^{bulk} MP2 CBS(aVDZ/aVTZ)	-	-	-	-1616	-1624	-	-
E_{int} MP2 CBS(aVTZ/aVQZ)	-1768	-1643	-	-	-	-	-
E_{int} MP2 CBS(awCVTZ/awCVQZ)	-1741	-1608	-	-	-	-	-
E_{int} LMP2 CBS(aVDZ/aVTZ)	-1748	-1617	-	-	-	-	-
E_{int} LNO-CCSD(T) CBS(aVDZ/aVTZ)	-1767	-1644	-	-	-	-	-
H_2O on TiO_2 anatase(101)	1	2	3	4	5	6	7
E_{int} MP2 aVDZ	-1273	-1266	-1118	-1107	-1074	-1061	-1059
E_{int}^{bulk} MP2 aVDZ	-	-	-	-1107	-1074	-1061	-1059
E_{int} MP2 CBS(aVDZ/aVTZ)	-1406	-1396	-1249	-1236	-1202	-	-
E_{int}^{bulk} MP2 CBS(aVDZ/aVTZ)	-	-	-	-1236	-1202	-	-
E_{int} MP2 CBS(aVTZ/aVQZ)	-1407	-1399	-	-	-	-	-
E_{int} MP2 CBS(awCVTZ/awCVQZ)	-1394	-1384	-	-	-	-	-

Continued on next page

TABLE 7: (continued)

E_{int} LMP2 CBS(aVDZ/aVTZ)	-1401	-1391	-	-	-	-	-
E_{int} LNO-CCSD(T) CBS(aVDZ/aVTZ)	-1419	-1409	-	-	-	-	-
NH_3 on TiO_2 anatase(101)	1	2	3	4	5	6	7
E_{int} MP2 aVDZ	-1348	-1333	-1272	-1262	-1223	-1209	-1206
E_{int}^{bulk} MP2 aVDZ	-	-	-	-1262	-1223	-1209	-1206
E_{int} MP2 CBS(aVDZ/aVTZ)	-1511	-1491	-1422	-1410	-1368	-	-
E_{int}^{bulk} MP2 CBS(aVDZ/aVTZ)	-	-	-	-1410	-1368	-	-
E_{int} MP2 CBS(aVTZ/aVQZ)	-1523	-1506	-	-	-	-	-
E_{int} MP2 CBS(awCVTZ/awCVQZ)	-1511	-1492	-	-	-	-	-
E_{int} LMP2 CBS(aVDZ/aVTZ)	-1506	-1485	-	-	-	-	-
E_{int} LNO-CCSD(T) CBS(aVDZ/aVTZ)	-1514	-1493	-	-	-	-	-

C. Multilevel ΔCC contribution through mechanical embedding with small clusters

Another quality of the quantum clusters generated by the SKZCAM protocol is that the difference between methods from cWFT, namely CCSD(T) and MP2, is relatively independent of the chosen cluster size. Thus, from the small quantum clusters generated by the SKZCAM protocol where local CCSD(T) is affordable, one can obtain a ΔCC contribution which can be used to elevate the MP2 bulk limit effectively up to the CCSD(T) bulk limit. This mechanical embedding procedure is based upon Morokumas ONIOM method [91] as well as the hybrid high-level:low-level approach of Sauer [61]. For each of the studied adsorbate–surface systems and adsorption configurations, we perform local CCSD(T) calculations up to the third SKZCAM cluster (where possible) as shown in Table 8. We set ΔCC to be the average of the three cluster, with its error taken to be the maximum deviation from the mean. We find that the deviations are very small; being 1 meV or less for most of the systems involving the MgO surface and TiO_2 anatase surface, while this error is typically below 5 meV for the TiO_2 rutile surface systems.

It should be noted that within this ΔCC contribution, there are also errors arising from using a local approximation for the CCSD(T) treatment. In Table 9, we have performed canonical CBS(DZ/TZ) CCSD(T) on the smallest cluster (corresponding to cluster '-2' for the TiO_2 clusters) for a selection of adsorbate–surface systems. Together with the canonical MP2 estimate we have already computed for these clusters, we compare these against the LNO-CCSD(T) and local-MP2 (LMP2) estimates of E_{int}. It can be seen that the errors in LMP2 and LNO-CCSD(T) are all below 5 meV, attesting to the accuracy of our final ΔCC contribution (using LNO-CCSD(T) and LMP2), where ΔCC itself has an MAD of 3 meV from the canonical ΔCC for this selection of systems. The final error which we compute for our ΔCC contribution is the root squared sum of the error from the mechanical embedding procedure and 5 meV for the maximum observed error from using the local approximation.

TABLE 8: Comparison of the ΔCC values for the clusters generated from SKZCAM protocol for all of the studied systems. The mean is calculated from the set of clusters used, with the error being the maximum deviation from the mean.

Cluster<br>System	1	2	3	Mean	Error
CH_4 on MgO(001)	-15	-16	-16	-16	0
Monolayer CH_4 on MgO(001)	-15	-16	-16	-16	0
C_2H_6 on MgO(001)	-20	-20	-20	-20	0
Monolayer C_2H_6 on MgO(001)	-19	-19	-20	-20	1
CO on MgO(001)	-7	-7	-9	-8	1
C_6H_6 on MgO(001)	24	24	25	24	1
Parallel N_2O on MgO(001)	-6	-7	-7	-6	1
Tilted N_2O on MgO(001)	17	16	16	16	1
Vertical-Hollow NO on MgO(001)	289	-	-	289	0
Vertical-Mg NO on MgO(001)	-68	-	-	-68	0
Bent-Bridge NO on MgO(001)	626	-	-	626	0
Bent-Mg NO on MgO(001)	-48	-	-	-48	0
Bent-O NO on MgO(001)	659	-	-	659	0
Dimer NO on MgO(001)	18	17	-	18	1
Monomer H_2O on MgO(001)	-26	-25	-26	-25	0
Tetramer H_2O on MgO(001)	-27	-27	-27	-27	0
Tilted CH_3OH on MgO(001)	-22	-21	-22	-21	1
Parallel CH_3OH on MgO(001)	-16	-17	-17	-17	0
Tetramer CH_3OH on MgO(001)	-27	-27	-27	-27	0
NH_3 on MgO(001)	-31	-30	-31	-31	1
Physisorbed CO_2 on MgO(001)	-30	-31	-32	-31	1
Chemisorbed CO_2 on MgO(001)	-218	-219	-224	-220	4
CH_4 on TiO_2 rutile(110)	-1	0	-	-1	1
Parallel CO_2 on TiO_2 rutile(110)	8	5	-	7	2
Tilted CO_2 on TiO_2 rutile(110)	-23	-24	-	-24	1
H_2O on TiO_2 rutile(110)	-29	-39	-	-34	5
CH_3OH on TiO_2 rutile(110)	-19	-27	-	-23	4
H_2O on TiO_2 anatase(101)	-18	-18	-	-18	0
NH_3 on TiO_2 anatase(101)	-8	-8	-	-8	0

TABLE 9: Comparing canonical (C-)MP2, canonical CCSD(T) and canonical ΔCC (in meV) against their local variants (i.e., LMP2, LNO-CCSD(T), L-ΔCC). This was compared for the first cluster generated by the SKZCAM protocol for the specific MgO adsorption site in Fig. 6, while it corresponds to cluster '−2' for the TiO_2 surfaces.

	C-MP2	L-MP2	L-MP2 Error	C-CCSD(T)	LNO-CCSD(T)	LNO-CCSD(T) Error	C-DeltaCC	(L-)DeltaCC	(L-)DeltaCC Error
CH_4 on MgO(001)	-71	-70	0	-91	-86	5	-20	-15	4
C_2H_6 on MgO(001)	-90	-92	2	-116	-112	4	-26	-20	6
CO on MgO(001)	-155	-153	2	-165	-160	5	-9	-7	3
Parallel N_2O on MgO(001)	-206	-203	2	-215	-209	6	-9	-6	3
Tilted N_2O on MgO(001)	-122	-120	2	-107	-104	4	15	17	2
Dimer NO on MgO(001)	-222	-217	5	-205	-199	6	17	18	2
Monomer H_2O on MgO(001)	-641	-639	2	-670	-665	5	-29	-26	3
Tilted CH_3OH on MgO(001)	-703	-701	2	-729	-723	7	-27	-22	5
Parallel CH_3OH on MgO(001)	-443	-442	1	-466	-458	8	-23	-16	6
NH_3 on MgO(001)	-551	-547	4	-583	-578	5	-32	-31	1
Physisorbed CO_2 on MgO(001)	-240	-237	3	-265	-267	2	-25	-30	5
CH_4 on TiO_2 rutile(110)	-395	-395	0	-398	-394	5	-3	2	4
Parallel CO_2 on TiO_2 rutile(110)	-454	-451	3	-467	-463	4	-13	-12	1
Tilted CO_2 on TiO_2 rutile(110)	-830	-828	3	-851	-850	0	-20	-23	2
H_2O on TiO_2 rutile(110)	-2469	-2466	3	-2477	-2477	0	-9	-12	3
CH_3OH on TiO_2 rutile(110)	-2921	-2916	5	-2922	-2921	1	-1	-5	4
H_2O on TiO_2 anatase(101)	-1874	-1871	3	-1896	-1895	1	-22	-23	1
NH_3 on TiO_2 anatase(101)	-1801	-1799	3	-1822	-1824	3	-20	-26	5
MAD	0	0	3	0	0	4	0	0	3

D. Further multilevel contributions for basis set and semi-core electron correlation

Besides the ΔCC contribution, it is possible to make further multilevel contributions (especially now that it has been automated in Section 10) in the vein of Pople's 'model chemistry' [92]. This lowers the cost of reaching an accurate estimate as these contributions (e.g., basis set size or frozen core treatment) need to only be evaluated on the small affordable clusters generated by the SKZCAM protocol. Specifically we consider further contributions to: (1) fix our basis set extrapolation treatment to more accurately reach the basis set limit and (2) incorporate electron correlation contributions beyond the valence electrons from semi-core electrons. Adding these contributions can lower the cost as it means that we can use cheaper settings (i.e., smaller basis sets while correlating only valence electrons) in the $E_{\text{int}}^{\text{bulk}}$ and ΔCC calculations. For the adsorbate–surface systems involving the TiO_2 surfaces and the open-shell NO monomers on the MgO surface, we have computed the MP2 $E_{\text{int}}^{\text{bulk}}$ and ΔCC with semi-core electrons (i.e., 2s2p on Mg or 3s3p on Ti) frozen in the correlation treatment. This has been particularly important towards making it possible to tackle these systems, involving more electrons or requiring unrestricted MP2/CCSD(T), more affordable. We use a Δ_{core} contribution to account for these missing effects at the MP2 level from the smaller and more affordable clusters of the SKZCAM protocol (up to cluster 3). The Δ_{core} contributions are shown in Table 10. We take the mean of the clusters as our final Δ_{core} estimate and use the maximum deviation from the mean as the error. The maximum deviation are all below 5 meV.

For all of the adsorbate–surface systems, we add a further Δ_{basis} contribution which corrects for potentials errors in our extrapolation treatment to reach the complete basis set limit; we used a two-point CBS(aVDZ/aVTZ) or CBS(awCDZ/awCVTZ) extrapolation. This contribution is calculated by considering the difference between the more accurate CBS(aVTZ/aVQZ) or CBS(awCVTZ/awCVQZ) extrapolation treatment with the CBS(aVDZ/aVTZ) or CBS(awCVDZ/awCVTZ) extrapolation treatment for the smallest clusters. The calculated Δ_{basis} contributions for the SKZCAM protocol clusters are shown in Table 11. We take the mean of the clusters as our final Δ_{basis} estimate and use the maximum deviation from the mean as the error, with most systems having errors below 5 meV, besides the larger systems (e.g., C_6H_6 and the tetramers on MgO(001)), where deviations may go up to 10 meV.

Both Δ_{core} and Δ_{basis} are important contributions which enable our final H_{ads} estimate to better match experiments while maintaining a low cost, with contributions up to 40 meV for the former and 13 meV in the latter.

TABLE 10: Comparison of the Δ_{core} values for the clusters generated from SKZCAM protocol for all of the studied systems. The mean is calculated from the set of clusters used, with the error being the maximum deviation from the mean.

Cluster<br>System	1	2	3	Mean	Error
Vertical-Hollow NO on MgO(001)	-18	-24	-24	-22	4
Vertical-Mg NO on MgO(001)	-22	-26	-28	-25	3
Bent-Bridge NO on MgO(001)	-19	-26	-28	-24	5
Bent-Mg NO on MgO(001)	-22	-27	-29	-26	3
Bent-O NO on MgO(001)	-9	-15	-18	-14	5
CH_4 on TiO_2 rutile(110)	-2	-3	-	-2	0
Parallel CO_2 on TiO_2 rutile(110)	-8	-7	-	-7	0
Tilted CO_2 on TiO_2 rutile(110)	8	10	-	9	1
H_2O on TiO_2 rutile(110)	37	44	-	41	4
CH_3OH on TiO_2 rutile(110)	28	35	-	31	4
H_2O on TiO_2 anatase(101)	13	15	-	14	1
NH_3 on TiO_2 anatase(101)	13	14	-	13	1

TABLE 11: Comparison of the Δ_{basis} values for the clusters generated from SKZCAM protocol for all of the studied systems. The mean is calculated from the set of clusters used, with the error being the maximum deviation from the mean.

Cluster	1	2	3	Mean	Error
System					
CH_4 on MgO(001)	3	0	2	2	1
Monolayer CH_4 on MgO(001)	3	1	2	2	1
C_2H_6 on MgO(001)	3	3	4	3	1
Monolayer C_2H_6 on MgO(001)	2	2	4	3	1
CO on MgO(001)	0	-1	3	1	2
C_6H_6 on MgO(001)	-15	-15	-2	-11	9
Parallel N_2O on MgO(001)	-2	-2	2	-1	2
Tilted N_2O on MgO(001)	-6	-6	-3	-5	2
Vertical-Hollow NO on MgO(001)	10	8	8	9	1
Vertical-Mg NO on MgO(001)	2	7	8	5	4
Bent-Bridge NO on MgO(001)	2	-5	-4	-2	4
Bent-Mg NO on MgO(001)	-2	2	2	1	3
Bent-O NO on MgO(001)	1	2	-6	-1	5
Dimer NO on MgO(001)	-4	-3	-1	-2	2
Monomer H_2O on MgO(001)	0	-3	3	0	3
Tetramer H_2O on MgO(001)	-19	-18	-9	-15	6
Tilted CH_3OH on MgO(001)	-1	-3	2	-1	3
Parallel CH_3OH on MgO(001)	17	19	18	18	1
Tetramer CH_3OH on MgO(001)	-18	-18	-11	-16	4
NH_3 on MgO(001)	-19	-20	-9	-16	7
Physisorbed CO_2 on MgO(001)	1	2	4	3	2
Chemisorbed CO_2 on MgO(001)	23	32	20	25	7
CH_4 on TiO_2 rutile(110)	1	1	-	1	0
Parallel CO_2 on TiO_2 rutile(110)	-1	-3	-	-2	1
Tilted CO_2 on TiO_2 rutile(110)	-6	-8	-	-7	1
H_2O on TiO_2 rutile(110)	-16	-20	-	-18	2
CH_3OH on TiO_2 rutile(110)	-16	-21	-	-19	2
H_2O on TiO_2 anatase(101)	0	-3	-	-2	1
NH_3 on TiO_2 anatase(101)	-13	-15	-	-14	1

E. The final E_{int} estimates and their error bars

To summarise the above sections, the final CCSD(T) interaction energy bulk-limit estimate, $E_{\text{int}}^{\text{CCSD(T)}}$, is given as the sum of the following contributions:

$$E_{\text{int}}^{\text{bulk CCSD(T)}} = E_{\text{int}}^{\text{bulk MP2}} + \Delta_{\text{basis}} + \Delta_{\text{core}} + \Delta\text{CC}. \quad (5)$$

The contributions are shown in Table 12 for all of the adsorbate–surface systems studied within this work. The series of clusters generated by the SKZCAM protocol provides a means to estimate the error from each of these contributions. For $E_{\text{int}}^{\text{bulk MP2}}$, we calculate the error in this term by finding the maximum deviation when a larger set of clusters are used to extrapolate $E_{\text{int}}^{\text{bulk MP2}}$ (with only the awCVDZ/aVDZ basis set) for the systems with the MgO surface (barring the chemisorbed CO_2 system). For the TiO_2 rutile(110) and anatase(101) surfaces, as well as the chemisorbed CO_2 on MgO(001), the larger clusters are used to estimate the maximum deviation. For ΔCC, Δ_{basis} and Δ_{core}, we estimate the error by taking the maximum deviation from the mean of the clusters used to calculate these terms. The final error on $E_{\text{int}}^{\text{bulk CCSD(T)}}$ is the root squared sum of the errors from each of these terms. It can be seen in Table 12 that the errors are typically below 10 meV for all of the MgO adsorbate–surface systems, with some of the TiO_2 adsorbate–surface systems having a larger error, but all are within chemical accuracy of 43 meV.

TABLE 12: Final E_{int} values (in meV) for the systems studied in this work. We show the individual terms which make up these final E_{int} values and also give final MP2, CCSD and CCSD(T) estimates, where the latter two are obtained by adding the ΔCC values to the final MP2 E_{int} value.

System	$E_{int}^{bulk\ MP2}$	ΔCC [CCSD(T)]	ΔCC [CCSD]	Δ_{basis}	Δ_{core}	$E_{int}^{autoSKZCAM}$ [MP2]	$E_{int}^{autoSKZCAM}$ [CCSD]	$E_{int}^{autoSKZCAM}$ [CCSD(T)]
CH_4 on MgO(001)	-108 ± 2	-16 ± 0	18 ± 7	2 ± 1	0 ± 0	-106 ± 2	-88 ± 7	-122 ± 2
Monolayer CH_4 on MgO(001)	-107 ± 2	-16 ± 0	18 ± 7	2 ± 1	0 ± 0	-105 ± 3	-87 ± 7	-121 ± 3
C_2H_6 on MgO(001)	-158 ± 3	-20 ± 0	32 ± 13	3 ± 1	0 ± 0	-155 ± 4	-122 ± 14	-175 ± 4
Monolayer C_2H_6 on MgO(001)	-144 ± 3	-20 ± 1	29 ± 13	3 ± 1	0 ± 0	-141 ± 3	-112 ± 13	-161 ± 3
CO on MgO(001)	-200 ± 3	-8 ± 1	33 ± 8	1 ± 2	0 ± 0	-199 ± 3	-166 ± 9	-207 ± 4
C_6H_6 on MgO(001)	-460 ± 2	24 ± 1	164 ± 24	-11 ± 9	0 ± 0	-470 ± 9	-307 ± 26	-446 ± 9
Parallel N_2O on MgO(001)	-249 ± 2	-6 ± 1	40 ± 6	-1 ± 2	0 ± 0	-250 ± 3	-210 ± 7	-256 ± 3
Tilted N_2O on MgO(001)	-179 ± 3	16 ± 1	64 ± 11	-5 ± 2	0 ± 0	-184 ± 4	-120 ± 11	-168 ± 4
Vertical-Hollow NO on MgO(001)	-244 ± 1	289 ± 0	334 ± 0	9 ± 1	-22 ± 4	-257 ± 4	76 ± 4	32 ± 4
Vertical-Mg NO on MgO(001)	26 ± 2	-68 ± 0	-49 ± 0	5 ± 4	-25 ± 3	6 ± 5	-43 ± 5	-62 ± 5
Bent-Bridge NO on MgO(001)	-661 ± 3	626 ± 0	693 ± 0	-2 ± 4	-24 ± 5	-688 ± 8	6 ± 8	-62 ± 8
Bent-Mg NO on MgO(001)	-53 ± 1	-48 ± 0	-23 ± 0	1 ± 3	-26 ± 3	-78 ± 5	-102 ± 5	-126 ± 5
Bent-O NO on MgO(001)	-647 ± 0	659 ± 0	720 ± 0	-1 ± 5	-14 ± 5	-661 ± 7	59 ± 7	-3 ± 7
Dimer NO on MgO(001)	-261 ± 2	18 ± 1	67 ± 5	-2 ± 2	0 ± 0	-263 ± 2	-197 ± 5	-246 ± 3
Monomer H_2O on MgO(001)	-677 ± 2	-25 ± 0	22 ± 9	0 ± 3	0 ± 0	-677 ± 4	-655 ± 10	-703 ± 4
Tetramer H_2O on MgO(001)	-421 ± 3	-27 ± 0	3 ± 7	-15 ± 6	0 ± 0	-436 ± 7	-433 ± 10	-463 ± 7
Tilted CH_3OH on MgO(001)	-765 ± 4	-21 ± 1	45 ± 14	-1 ± 3	0 ± 0	-765 ± 5	-720 ± 14	-787 ± 5
Parallel CH_3OH on MgO(001)	-508 ± 1	-17 ± 0	50 ± 10	18 ± 1	0 ± 0	-490 ± 1	-440 ± 10	-506 ± 1
Tetramer CH_3OH on MgO(001)	-469 ± 4	-27 ± 0	18 ± 10	-16 ± 4	0 ± 0	-485 ± 6	-466 ± 12	-511 ± 6
NH_3 on MgO(001)	-610 ± 5	-31 ± 1	9 ± 16	-16 ± 7	0 ± 0	-626 ± 9	-618 ± 18	-657 ± 9
Physisorbed CO_2 on MgO(001)	-280 ± 1	-31 ± 1	21 ± 7	3 ± 2	0 ± 0	-277 ± 2	-256 ± 7	-308 ± 2
Chemisorbed CO_2 on MgO(001)	-3309 ± 32	-220 ± 4	-447 ± 7	25 ± 7	0 ± 0	-3284 ± 32	-3731 ± 33	-3504 ± 32
CH_4 on TiO_2 rutile(110)	-267 ± 2	-1 ± 1	53 ± 0	1 ± 0	-2 ± 0	-268 ± 2	-216 ± 2	-269 ± 2
Parallel CO_2 on TiO_2 rutile(110)	-408 ± 4	7 ± 2	71 ± 1	-2 ± 1	-7 ± 0	-417 ± 4	-346 ± 4	-410 ± 5
Tilted CO_2 on TiO_2 rutile(110)	-470 ± 6	-24 ± 1	8 ± 1	-7 ± 1	9 ± 1	-469 ± 7	-460 ± 7	-493 ± 7
H_2O on TiO_2 rutile(110)	-1299 ± 33	-34 ± 5	-68 ± 6	-18 ± 2	41 ± 4	-1276 ± 33	-1344 ± 34	-1310 ± 33
CH_3OH on TiO_2 rutile(110)	-1624 ± 36	-23 ± 4	-31 ± 5	-19 ± 2	31 ± 4	-1612 ± 36	-1642 ± 37	-1634 ± 37
H_2O on TiO_2 anatase(101)	-1202 ± 15	-18 ± 0	-32 ± 0	-2 ± 1	14 ± 1	-1190 ± 16	-1222 ± 16	-1208 ± 16
NH_3 on TiO_2 anatase(101)	-1368 ± 18	-8 ± 0	-35 ± 1	-14 ± 1	13 ± 1	-1369 ± 18	-1404 ± 18	-1377 ± 18

F. Validating the SKZCAM protocol beyond metal-oxides

To check the validity of the SKZCAM protocol and thus, autoSKZCAM framework, beyond metal-oxides towards surfaces of traditional ionic materials, we also performed additional calculations to obtain the E_{int} for H_2O on LiH(001) and acetylene on NaCl(001). These two systems follow the same set of clusters and calculations as MgO(001) described in the previous subsections. An E_{int} of -265 ± 9 meV for H_2O on LiH(001) computed with the SKZCAM protocol is shown in Table 13 and compared to previous calculations by Tsatsoulis *et al.* [93], being within the statistical error bars of DMC (-250 ± 7 meV) and an alternative (gas-phase) cluster CCSD(T) approach (-256 meV).

TABLE 13: Final E_{int} values (in meV) with the autoSKCAM framework for the water adsorbed on LiH(001). The DMC and CCSD(T) values are taken from Ref. 93.

H_2O on LiH(001)	Contribution [meV]
$E_{\text{int}}^{\text{bulk MP2}}$	-247 ± 1
ΔCC	-17 ± 5
Δ_{basis}	-2 ± 8
Final autoSKZCAM E_{int}	-265 ± 9
Cluster CCSD(T) [93]	-256
DMC [93]	-250 ± 7

For acetylene on NaCl(001), we have inferred the experimental estimate of E_{int} from previous experimental estimates by Cabello-Cartagena *et al.* [94] and Dunn and Ewing [95]. This is shown in Table 14, where the lateral interactions [96, 97] are first removed from the H_{ads} estimates by the experiments, followed by a correction to convert the dilute limit H_{ads} to E_{int} using revPBE-D4. The final estimates of E_{int} for the dilute limit go from -130 meV to -237 meV by Cabello-Cartagena *et al.* and Dunn and Ewing, respectively, covering a large range of 107 meV. The E_{int} estimate by the SKZCAM protocol of -181 ± 3 lies in the middle of this range, confirming its applicability to this system.

TABLE 14: Converting to the experimental values for the adsorption enthalpy H_{ads} to an interaction energy E_{int} by removing lateral interaction and the contributions to convert from an H_{ads} to E_{int} at the revPBE-D4 level (encapsulated in $\Delta_{E_{\text{int}}}^{H_{\text{ads}}}$[revPBE-D4]).

	Cabello-Cartagena et al. [94]	Dunn and Ewing [95]
Coverage (θ)	1.0	0.5
H_{ads}	-249	-311
Lateral Interaction [96, 97]	-140	-95
$\Delta_{E_{\text{int}}}^{H_{\text{ads}}}$[revPBE-D4]	18	18
E_{int}	-127	-234

TABLE 15: Final E_{int} values (in meV) with the autoSKCAM framework for acetylene adsorbed on NaCl(001). The (inferred) experimental E_{int} is explained in the text and shown in Table 14.

Acetylene on NaCl(001)	Contribution [meV]
$E_{\text{int}}^{\text{bulk MP2}}$	-203 $\pm$ 1
ΔCC	22 $\pm$ 1
Δ_{basis}	0 $\pm$ 2
Final autoSKZCAM E_{int}	-181 $\pm$ 3
Experiment [from Table 14]	-127 to -234

G. Improvements to SKZCAM protocol in present work

We highlight here that because the SKZCAM protocol has been automatised in the present work (described in Section 10), it has allowed for a significant lowering of computational cost compared to previous applications of the SKZCAM protocol [85, 98]. The improvements are as follows:

1. More intermediate layers in the ONIOM mechanical embedding treatment. For example, we have added the Δ_{basis} and Δ_{core} terms to the final E_{int}, enabling cheaper calculations of ΔCC and $E_{\text{int}}^{\text{bulk MP2}}$ – the two dominant contributions to the overall computational cost – including only valence, no longer requiring semicore, electrons in the correlation treatment (due to Δ_{core}) and a smaller basis set treatment (due to Δ_{basis}).

2. The use of multiple different codes that are efficient for different types of calculations. We can utilise the ORCA code to perform efficient MP2 calculations [68] while still leveraging the LNO-CCSD(T) implementation [70, 71] in MRCC. Previously, we were limited towards only using one code due to the additional manual overhead.

Overall, these improvements enable significant lowering of costs. To put these improvements into context, our previous simulation [85] of CO on MgO(001) required arond 20,000 CPUh to perform while these improvements enable a cost to be lowered by two orders of magnitude to around 600 CPUh. Without these improvements, the application to the TiO_2 surface systems would have been largely unfeasible as well.

7. CONTRIBUTIONS FOR THE COHESIVE AND CONFORMATIONAL ENERGY FROM CWFT IN SELECTED SYSTEMS

For the adsorption of monomers on the MgO surface, the definition of E_{int} has been defined in Fig. 3, where its difference with the adsorption energy E_{ads} is the energy to relax the monomer molecule and surface from their geometries in the adsorbate–surface complex to their equilibrium geometry, given by the quantity E_{rlx}. When computing the adsorption energy of clusters and the monolayers on the MgO(001) surface, there are additional contributions towards E_{ads} arising from the cohesive energy E_{coh} (or lateral interaction) that binds the molecules together. In general, E_{rlx} does not form a large contribution towards E_{ads}, with E_{int} being the dominant contribution, oftentimes more than 80% of H_{ads}. This is one of the reasons why we have computed E_{rlx} with DFT. However, it is not the case for systems where the molecule undergoes significant conformational changes (due to e.g., charge transfer). This is specifically the case for the chemisorbed CO_2 on MgO(001), where there is a large energy change of almost $\sim$2 eV to bring the CO_2 from its bent geometry back into its linear geometry. This large conformational energy change E_{conf} is a quantity that is highly sensitive to the DFA. As such, for this system, we have computed E_{conf} with CCSD(T) - a negligible overall computational cost for the CO_2 molecule. We elaborate further on the value of these terms in the next sections.

A. Conformational energy of the chemisorbed CO_2 on MgO(001)

As illustrated in Fig. 7, for the chemisorbed CO_2 on MgO(001), we have effectively broken up the original E_{rlx}, containing the relaxation energy for both the molecule and surface, such that it only pertains to the surface, with E_{conf} corresponding to the relaxation energy for the molecule. The importance of this contribution is shown in Table 16 where the conformational energy change is predicted by several DFAs (as well as MP2, CCSD) and find that they can differ by more than 300 meV from CCSD(T). These are well-known effects arising from the delocalisation error inherent in most DFAs [99]. The canonical MP2, CCSD and CCSD(T) calculations were performed in `MRCC` with the CBS(aVTZ/aVQZ) basis set treatment.

TABLE 16: Comparison of the E_{conf} values (in meV) for chemisorbed CO_2 chemisorbed on MgO for several DFAs as well as MP2, CCSD and CCSD(T).

	E_{conf} [meV]
PBE-D2[Ne]	1841
revPBE-D4	1797
vdW-DF	1763
rev-vdW-DF2	1836
PBE0-D4	2153
B3LYP-D2[Ne]	2075
MP2	2045
CCSD	2304
CCSD(T)	2094

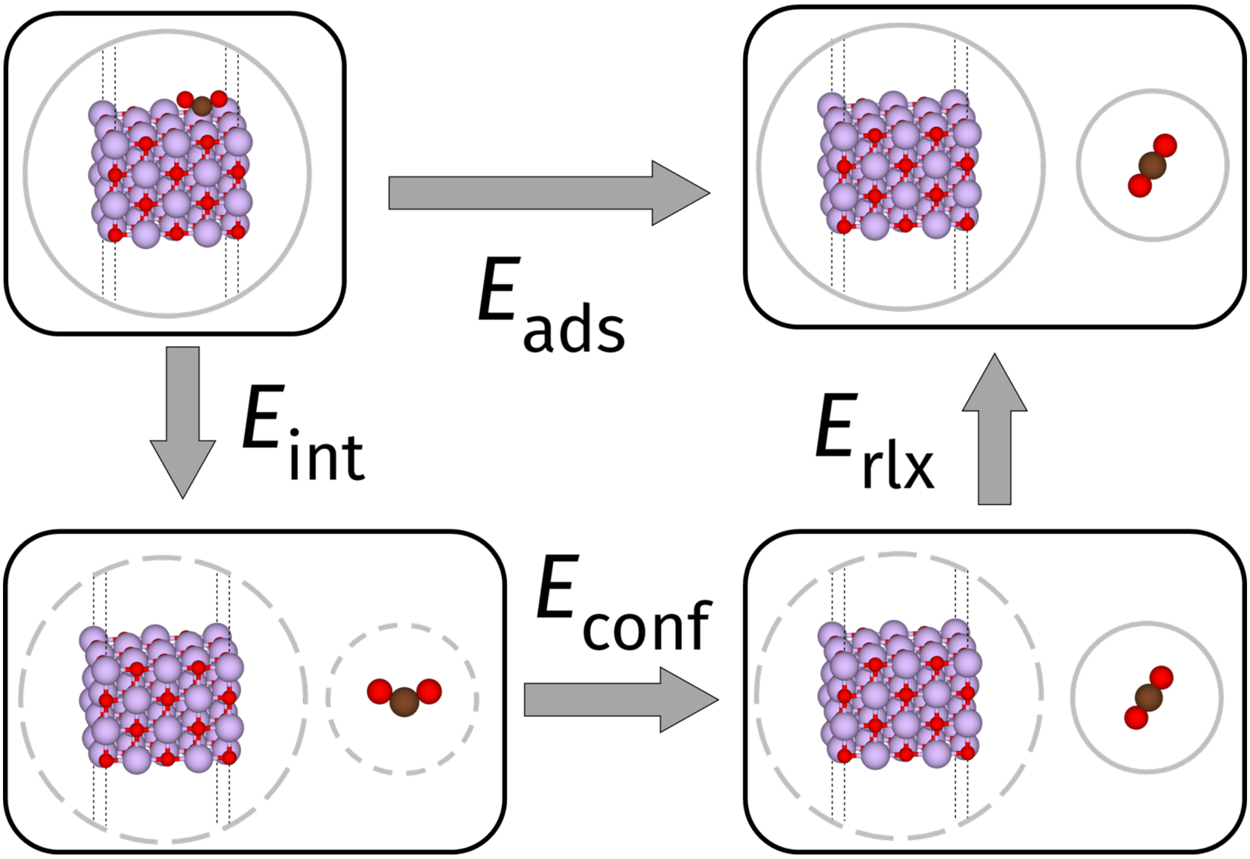

FIG. 7: The contributions to the adsorption energy E_{ads} for the chemisorbed CO_2 on MgO(001). Beyond the interaction energy E_{int}, there is E_{conf} - the relaxation energy to return the molecule in its geometry within the adsorbate–surface complex to its equilibrium geometry, leaving E_{rlx} to be the relaxation energy to bring the surface (at the geometry of the adsorbate–surface complex) into its equilibrium geometry. The circles represent a single system/calculation, with a dashed circle indicating a geometry fixed to that found in the adsorbate–surface complex while a line circle indicates an equilibrium geometry.

TABLE 17: Table showcasing how the final CCSD(T)-quality E_{coh} (in meV) is computed. CCSD(T) (and other WFT methods) is used to compute the two-body (2B) contribution to the many-body expansion of the E_{coh}^{gas} - the cohesive energy of the alkane monolayer in the absence of the MgO surface. This contribution is used to correct the 2B contribution to E_{coh}^{gas} for revPBE-D4 (i.e., it is used to account for 3B and beyond contributions). We reach the final E_{coh} by incorporating the effect of the MgO surface at the revPBE-D4 level (i.e., the difference between E_{coh} and E_{coh}^{gas}).

	CH_4 Monolayer			C_2H_6 Monolayer		
	$E_{coh}^{2B\ gas}$	E_{coh}^{gas}	E_{coh}	$E_{coh}^{2B\ gas}$	E_{coh}^{gas}	E_{coh}
PBE-D2[Ne]	-	-53	-40	-	-125	-106
revPBE-D4	-46	-42	-31	-79	-62	-48
vdW-DF	-	-72	-58	-	-88	-67
rev-vdW-DF2	-	-40	-43	-	-69	-83
PBE0-D4	-	-43	-34	-	-77	-64
B3LYP-D2[Ne]	-	-39	-37	-	-124	-124
MP2	-15	-11	-	-58	-41	-27
CCSD	-33	-29	-18	-59	-43	-29
CCSD(T)	-40	-37	-25	-92	-75	-61

B. Cohesive energy in CH_4 and C_2H_6 monolayers on MgO(001)

As illustrated in Fig. 8 for monolayer CH_4 on MgO(001), the E_{coh} cohesive energy term corresponds to the energetic contribution to E_{ads} arising from the lateral interactions of the molecules on the surface, with E_{int} corresponding to the interaction energy of the individual molecules (as monomers) on the MgO surface. The choice to compute E_{coh} and E_{int} separately, as opposed to computing their sum: E_{int}^{ML} directly is because the SKZCAM protocol, as an embedding approach, can only calculate E_{int} for localised phenomena such as the adsorption of monomers. Practically, E_{coh} is computed by first computing E_{int} and subtracting it from E_{int}^{ML}. The computed E_{coh} is shown in Table 17 and it can be seen that across the different functionals, it can vary by over 40 meV, clearly requiring the need to go beyond DFT to treat this interaction.

It is not directly possible to elevate E_{coh} up to CCSD(T) quality as it involves (minor effects) arising from the presence of the MgO surface. Instead, we have to consider the cohesive energy E_{coh}^{gas} of monolayer in the absence of the surface (i.e., in the gas phase). Specifically, it is possible

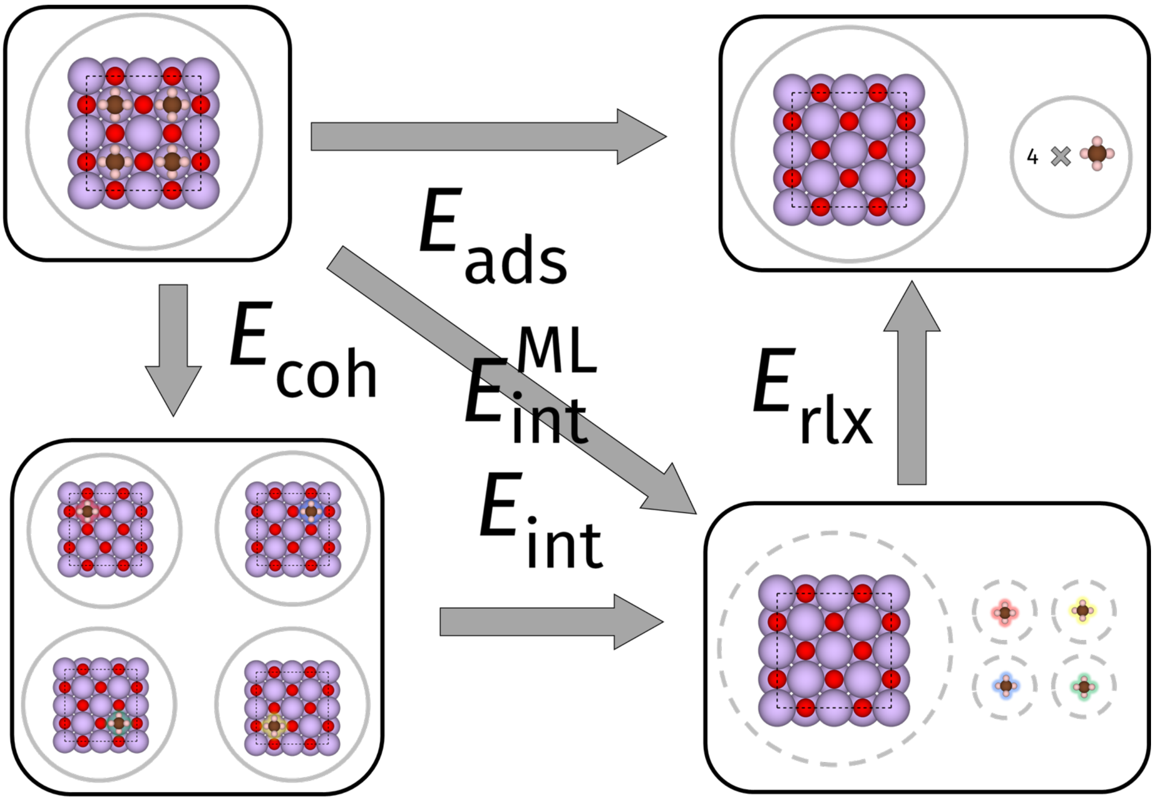

FIG. 8: The contributions to the adsorption energy E_{ads} for the monolayer CH_4 on MgO(001). Here, the E_{rlx} contribution remains the same as in Fig. 3. There is now an additional cohesive energy E_{coh} term which represents the lateral interactions between the molecules within the monolayer (in the presence of the MgO surface). Under this definition, the interaction energy E_{int} is calculated by treating the four molecules of the monolayer as individual monomers on the MgO(001) surface. The circles represent a single system/calculation, with a dashed circle indicating a geometry fixed to that found in the adsorbate–surface complex while a line circle indicates an equilibrium geometry.

to make a many-body expansion of E_{coh}^{gas}, incorporating 2-body (2B) contributions (see Fig. 9 and beyond. As we show for revPBE-D4, the 2B contribution ($E_{coh}^{2B\ gas}$) makes up the most dominant contribution of E_{coh}^{gas}, differing by 4 meV and 17 meV for the CH_4 and C_2H_6 monolayers respectively. We have computed the CCSD(T) $E_{coh}^{2B\ gas}$ contribution and from this, we can correct this (major) part of E_{coh}^{gas} and subsequently E_{coh}. We also make use of the frozen-natural orbital (FNO) approximation to speed up these calculations. This treatment has been performed before by Alessio *et al.* [53] and Tosoni *et al.* [49] and we come in excellent agreement; differences could arise from differing geometries or CCSD(T) being more accurate than MP2.

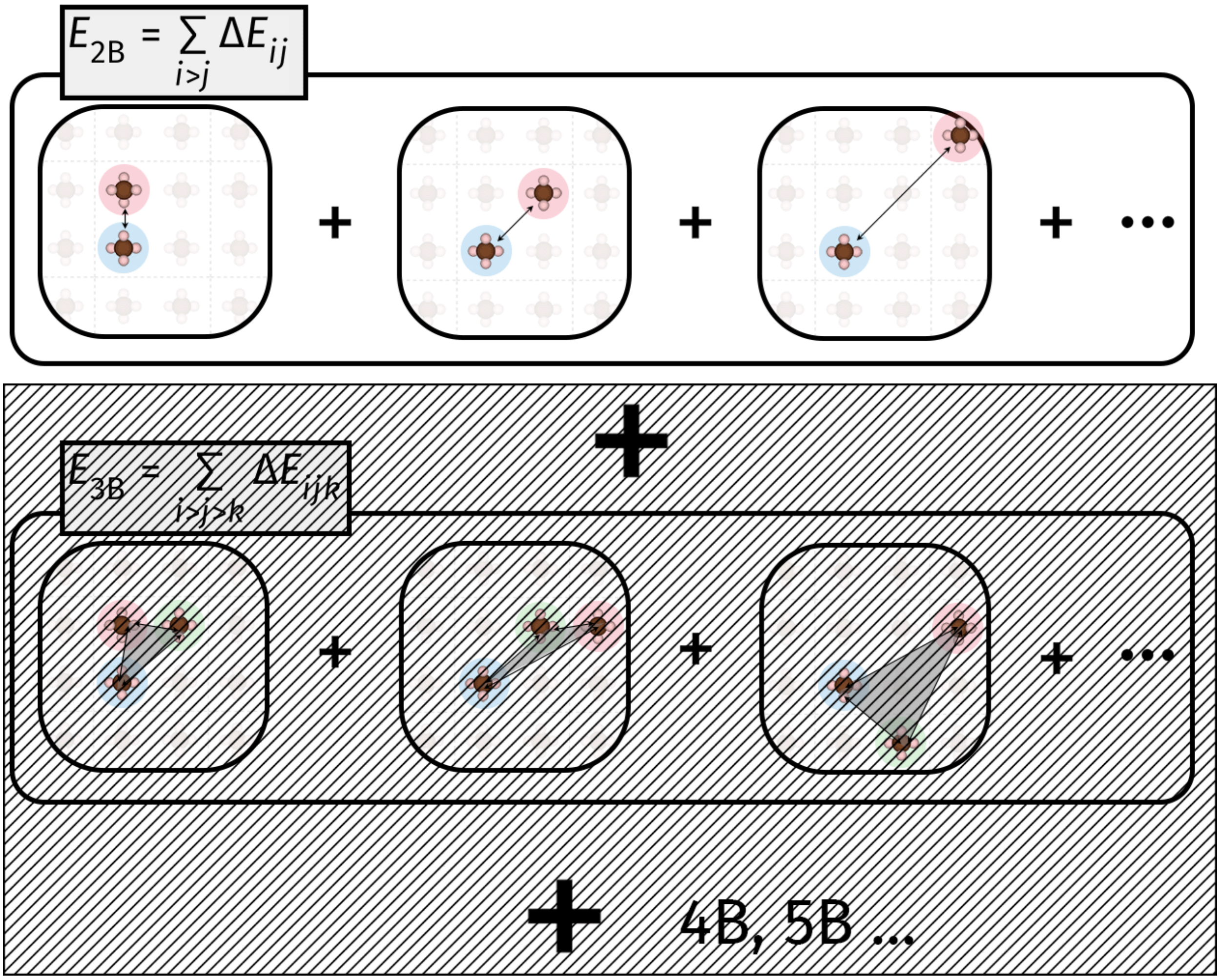

FIG. 9: Showcasing the many-body expansion to obtain $E_{\mathrm{coh}}^{\mathrm{gas}}$ of alkane monolayers (using CH_4 as an example). We compute the 2B contribution at the CCSD(T) level and treat the higher-body contributions with revPBE-D4.

C. Cohesive and dissociation energy in H_2O and CH_3OH clusters on MgO(001)

For the (non-dissociated) molecular H_2O and CH_3OH clusters on MgO(001), we partition the E_{ads} into contributions from E_{int}, E_{rlx} and E_{coh}, as seen in Fig. 10 Here, E_{int} is defined to be the interaction energy required to remove the entire cluster from the surface, taking the cluster to be the 'molecule'. The cohesive energy is then the binding energy to break the cluster into its separate molecules, all within the gas phase and having their geometries fixed to that within the cluster-surface complex. Finally E_{rlx} is the energy to relax the molecules and surface into their equilibrium geometries.

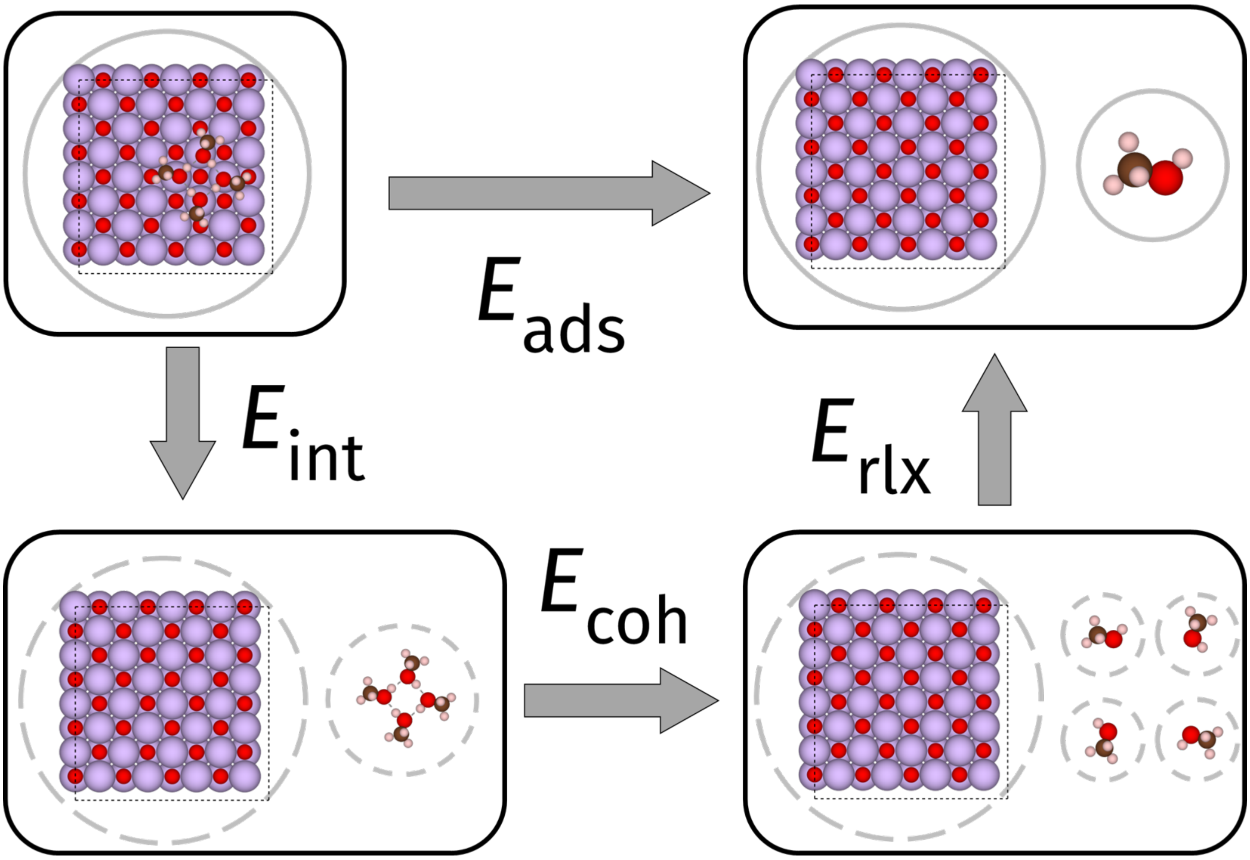

FIG. 10: The contributions to the adsorption energy E_{ads} for the tetramer CH_3OH cluster on MgO(001). Here, the E_{rlx} contribution remains the same as in Fig. 3. There is now an additional cohesive energy E_{coh} term which represents the lateral interactions between the molecules within the cluster (in the gas phase). Under this definition, the interaction energy E_{int} is calculated by treating the cluster as a single 'molecule' that first desorbs from the surface. The circles represent a single system/calculation, with a dashed circle indicating a geometry fixed to that found in the adsorbate–surface complex while a line circle indicates an equilibrium geometry.

TABLE 18: Comparison between several DFAs, MP2 and CCSD against CCSD(T) for the cohesive energy E_{coh} per monomer (in meV) of the tetramer CH_3OH and H_2O cluster.

	CH_3OH	H_2O
PBE-D2[Ne]	-380	-309
revPBE-D4	-328	-261
vdW-DF	-291	-240
rev-vdW-DF2	-357	-289
PBE0-D4	-367	-302
B3LYP-D2[Ne]	-371	-303
MP2	-341	-276
CCSD	-311	-269
CCSD(T)	-336	-281

The cohesive energy is computed for the revPBE-D4 geometry for the ensemble of DFAs as well as MP2, CCSD and CCSD(T) in Table 18. There is a range of 90 meV and 70 meV between the DFAs, highlighting the need to move towards CCSD(T) for high accuracy.

To calculate the adsorption energy E_{ads} of the partially dissociated clusters of H_2O and CH_3OH on MgO(001), we compute an additional term E_{diss} which accounts for the energy difference/stabilisation between the molecularly adsorbed and partially-dissociated forms of the H_2O and CH_3OH tetramers (see Fig. 11). We use the revPBE-D4 geometry of the molecular and partially-dissociated forms and have computed this energy using the ensemble of DFAs, as shown in Section 8 E.

D. Cohesive energy of NO dimer on MgO(001)

As illustrated in Fig. 12, the contributions to the E_{ads} of the NO dimer on MgO(001) differ slightly from those of the H_2O and CH_3OH tetramers. The definition of E_{int} remains the same, treating the dimer as the 'molecule'. However, the relaxation energy E_{rlx} now pertains to the the energy to bring the dimer from its geometry in the dimer-surface complex into its equilibrium geometry (together with the surface) and E_{coh} now represents the binding energy of the dimer against the separate monomers. This new definition of E_{coh} was chosen because the binding energy of the NO dimer is a well-studied topic both with experiments [100, 101] and computational

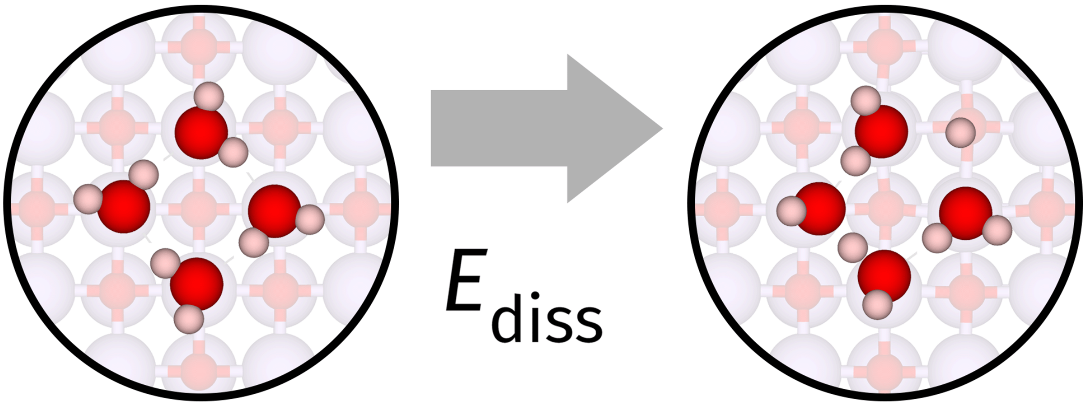

FIG. 11: The dissociation energy E_{diss} is defined as the energetic stabilisation to form a dissociated cluster from the original molecularly adsorbed cluster. We use the H_2O tetramer cluster as the example.

TABLE 19: Comparison of experiment and MRMP2 against several DFAs and CCSD(T) for the cohesive energy E_{coh} per monomer (in meV) of the NO dimer.

Method	E_{coh} [meV]
PBE-D2[Ne]	-239
revPBE-D4	-192
vdW-DF	-160
rev-vdW-DF2	-281
PBE0-D4	53
B3LYP-D2[Ne]	63
CCSD(T) [105]	-22
Experiment [104]	-61 to -82
MRMP2(18,14) [104]	-75

simulations [102, 103], with a complete summary of previous work found in Ref. 104.

As a multireference problem, DFT has trouble with getting E_{coh} right, clearly seen in Table 19 where there is an 344 meV range in E_{coh} across the DFAs that we have studied. Relative to the experimental E_{coh} window of −61 to −82 meV per monomer (or 121−165 meV in terms of binding energy), CCSD(T) has been shown to underbind this quantity with a value of −22 meV. Previous work with multireference second-order perturbation theory (MRMP2) using a large

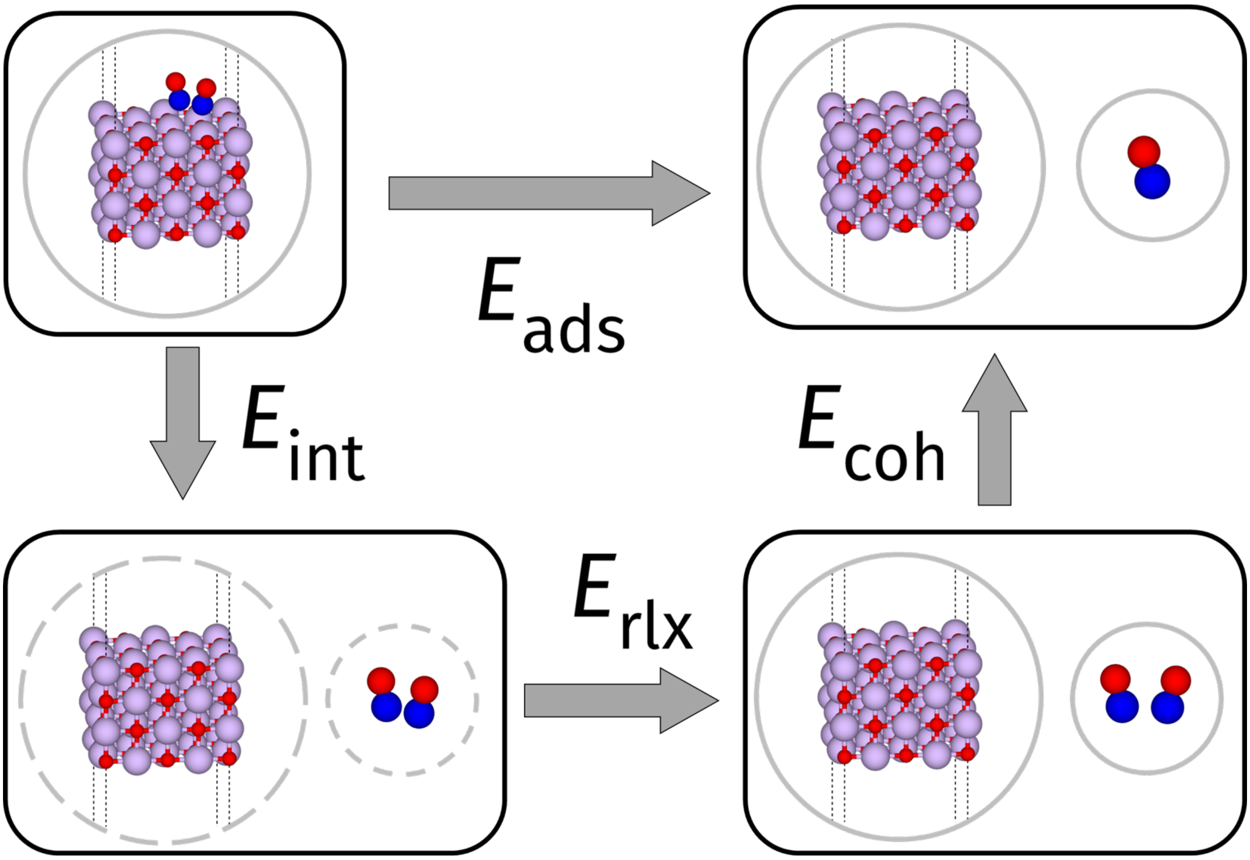

FIG. 12: The contributions to the adsorption energy E_{ads} for the NO dimer on MgO(001). Here, the interaction energy E_{int} is calculated by treating the dimer as a single 'molecule' that first desorbs from the surface. Then, E_{rlx} represents the relaxation energy for the surface into its equilibrium geometry and the dimer into its equilibrium geometry (as a dimer). The cohesive energy E_{coh} term which represents the binding energy to bring the dimer to two separate monomers, all in their equilibrium geometry. The circles represent a single system/calculation, with a dashed circle indicating a geometry fixed to that found in the adsorbate–surface complex while a line circle indicates an equilibrium geometry.

(18,14) active space have demonstrated much better agreement [104] with an E_{coh} of -75 meV. One of the advantages of the partitioning of the E_{ads} in Fig. 12 is that we have broken down E_{ads} into various contributions which can be tackled with different methods. Specifically, E_{int} can be reliably treated with CCSD(T), while E_{rlx} can be treated with DFT and E_{coh} can be treated with multireference methods. This allows us to use the previously computed MRMP2 value [104] of E_{coh} in our final E_{ads} and H_{ads}.

8. GEOMETRY RELAXATION AND ENTHALPIC CONTRIBUTIONS FROM A DFT ENSEMBLE

The autoSKZCAM framework extends the accurate E_{int} computed with the SKZCAM protocol towards computing an accurate H_{ads} estimate that can be compared directly with experiments. The aim is to make the calculation of H_{ads} affordable and to decrease the number of CCSD(T)-level calculations required, especially on the contributions where it isn't needed. Specifically, the autoSKZCAM framework uses DFT to compute the remaining geometrical relaxation E_{rlx}, zero-point vibrational E_{ZPV} and temperature E_{T} contributions to enable the calculation of H_{ads} within Eqs. 1 and 2. These terms make overall small contributions to the final H_{ads}, thus errors due to the approximate nature of DFT are not expected to alter the numbers significantly. Furthermore, these are quantities that do not depend on the absolute value of the potential energy surface (as in the case of E_{int}) but on 'relative' changes around the minima to e.g., compute vibrational frequencies or relaxation energies, which DFT can perform accurately for.

To ensure reliable estimates with error bars, we use an ensemble/set of density functional approximations (DFAs). For MgO, we used the 6 high-accuracy DFAs within the ensemble: PBE-D2[Ne] [49, 106], revPBE-D4 [4, 5], vdW-DF [107], rev-vdW-DF2 [108], PBE0-D4 [5, 109] and B3LYP-D2[Ne] [49, 110], where[Ne] denotes that the Neon D2 parameters [111] have been used on the Mg atom [49], or subsets of them as we discuss later. For the TiO_2 systems, we used the: PBE-TS/HI [106, 112], revPBE-D4, vdW-DF, rev-vdw-DF2, r^{2}SCAN-rVV10 [113] and HSE06-D4 [5, 114] functionals. These DFAs were chosen because they are generally expected to perform well for their respective surfaces for both E_{int} and unit cell lattice parameters (see Tables 20, 21 and 22 for the MgO, TiO_2 rutile(110) and anatase(101) surfaces respectively), allowing us to probe a sensible range of values as close as possible to the true answer. The use of an ensemble of DFAs allows us to take averages for improved estimates and to give error estimates on E_{rlx} and E_{ZPV} and E_{T} which we will discuss in Section 8 C and 8 D respectively. Their specific computational details are provided in Section 8 A.

A. Computational details for periodic density functional theory

Periodic DFT were performed with the Vienna *Ab-Initio* Simulation Package 6.3.0 [117, 118] (VASP). For each of the three surfaces, we used an ensemble of 6 DFAs to calculate the terms that make up H_{ads}. The electronic structure parameters [k-point grid, energy cutoff and projected

TABLE 20: Lattice parameter (in Å) and H_2O E_{int} for MgO(001) obtained from the DFT ensemble. These are compared to experiment [115] for the lattice parameter and the autoSKZCAM E_{int}.

	Lattice Parameter a	H_2O E_{int}
PBE-D2[Ne]	4.234	-693
revPBE-D4	4.220	-637
vdW-DF	4.273	-567
rev-vdW-DF2	4.220	-711
PBE0-D4	4.175	-695
B3LYP-D2[Ne]	4.202	-618
Reference	4.214	-702

TABLE 21: Lattice parameter (in Å) and H_2O E_{int} for TiO_2 rutile(110) obtained from the DFT ensemble. These are compared to experiment [116] for the lattice parameter and the autoSKZCAM E_{int}.

	Lattice Parameter a	Lattice Parameter c	H_2O E_{int}
PBE-TS/HI	4.611	2.970	-1282
revPBE-D4	4.598	2.958	-1214
vdW-DF	4.685	2.995	-1026
rev-vdW-DF2	4.618	2.961	-1344
r^2SCAN-rVV10	4.590	2.957	-1552
HSE06-D4	4.559	2.940	-1410
Reference	4.587	2.954	-1310

augmented wave (PAW) potentials] are provided in Table 23. An energy cutoff of 600 eV was used for most of the DFAs, although this was reduced to 520 eV for the hybrid HSE06-D4 calculations on the TiO_2 surface systems. For the HSE06-D4 calculation relaxations, we lower its cost by using a reduced Γ-point grid (via `NKRED`) for the exact exchange contribution to the total energy and use a 18 electron core PAW potential on the Ti atoms. A Γ-centered k-point mesh was used in all the systems, with the k-point grids chosen to converge E_{int} to 1 meV.

The three surfaces used to model the adsorbate–surface systems are shown in Fig. 13. For the MgO system, we perform calculations on a 4 layer slab, where the bottom two layers are fixed.

TABLE 22: Lattice parameter (in Å) and H_2O E_{int} for TiO_2 anatase(101) obtained from the DFT ensemble. These are compared to experiment [116] for the lattice parameter and the autoSKZCAM E_{int}.

	Lattice Parameter a	Lattice Parameter c	H_2O E_{int}
PBE-TS/HI	3.789	9.659	-1095
revPBE-D4	3.790	9.548	-1096
vdW-DF	3.839	9.767	-917
rev-vdW-DF2	3.798	9.590	-1179
r^2SCAN-rVV10	3.785	9.531	-1392
HSE06-D4	3.751	9.540	-1224
Reference	3.782	9.502	-1207

The majority of systems used a 4×4, with an 8×8 supercell used for C_6H_6 and the CH_3OH and H_2O clusters. The 2×2×1 k-point grid used with the 4×4 supercell was reduced to a Γ-point grid for the 8×8 supercell. The TiO_2 rutile(110) surface slab consisted of a p(4×2) supercell with 5 tri-layers (and the bottom three fixed), while the anatase(101) surface slab consisted of a (3×1) supercell with 4 tri-layers and the bottom layer fixed. All surfaces incorporated 15Å of vacuum and were generated through a geometrical relaxation performed with a force convergence cutoff of 0.01 eV/Å. Subsequently, a molecule was added onto the surface and relaxed with the same force convergence cutoff. Vibrational frequency calculations were performed for a subset of the DFT ensemble (see Section 8 D) using a finite-differences approach with `POTIM=0.01Å` displacements, one in the positive and negative direction along the three Cartesian directions (i.e., `NFREE=2`). The self-consistent field cycles were set to an energy convergence of 10^{-8} eV for the vibrational frequency calculations, with 10^{-7} eV as standard for geometry optimisations.

B. The relaxation energy

For the adsorption of monomers on the surfaces, the relaxation energy E_{rlx} is defined as the energy change when the surface and molecule are relaxed from their geometry in the adsorbate–surface complex to their equilibrium geometries. This definition persists for the tetramer CH_3OH and H_2O clusters and the monolayer alkanes on MgO(001), where the individual monomers in the cluster or monolayer are compared to their equilibrium geometires in the gas-phase. For

TABLE 23: DFT parameters used for the three different surfaces. The parameters for hybrids are also different from those for the metaGGA, GGA and vdW-inclusive functionals, grouped as (meta)GGA in the table. The number of layers in parentheses indicates the number of layers fixed at the bottom of the slab. The *k*-points in parenthesis indicates the *k*-point mesh used for the exact exchange potential. We used the PBE version 54 set of PAW potentials for all the calculations.

	MgO(001)		TiO_2 rutile(110)		TiO_2 anatase(101)	
	(meta)GGA	hybrid	(meta)GGA	hybrid	(meta)GGA	hybrid
Supercell Size	4×4	4×4	p(4×2)	p(4×2)	3×1	3×1
Number of Layers	4(2)	4(2)	5(3) O-Ti-O	5(2) O-Ti-O	8(2) O-Ti-O layers	8(2) O-Ti-O
k-point Mesh	2×2×1	2×2×1(2×2×1)	2×2×1	2×2×1(1×1×1)	3×3×1	3×3×1(1×1×1)
Energy cutoff	600	600	600	520	600	520
Vacuum	15	15	15	13	15	13
Metal PAW potential	Mg_pv (4e core)	Mg_pv (4e core)	Ti_pv (12e core)	Ti (18e core)	Ti_pv (12e core)	Ti (18e core)

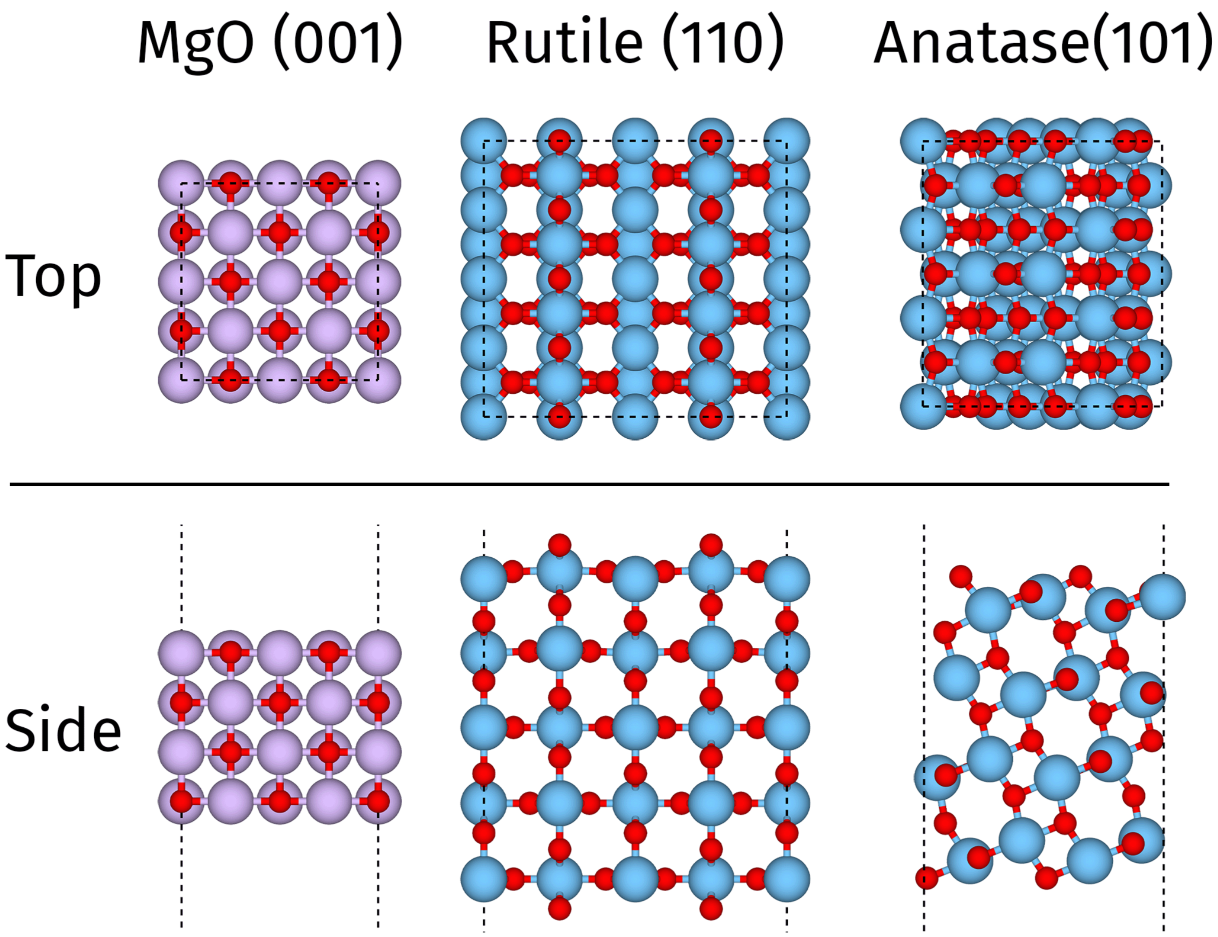

FIG. 13: Top and side views for the MgO(001), TiO_2 rutile(110) and TiO_2 anatase(101) surfaces.

the NO dimer, this definition slightly changes to be the energy change from the dimer on the MgO(001) surface to the dimer's equilibrium geometry for reasons discussed in Section 7 D. For the chemisorbed CO_2 on the MgO(001) surface, E_{rlx} now only corresponds to the relaxation of the surface, with the relaxation of the molecule encapsulated in the E_{conf} term that is treated at the CCSD(T) level, discussed in Section 7 A. The result is that E_{rlx} is a small quantity relative to the overall E_{ads}, as we show for the DFT ensemble in Tables 24 and 25 for the MgO and TiO_2 surfaces respectively. As discussed in the next section, we use revPBE-D4 to generate the adsorbate–surface geometries, and hence use the E_{rlx} term generated by this DFA. For the NO dimer system, we have opted to use the geometry and E_{rlx} from B3LYP-D2[Ne] as revPBE-D4 strongly overestimates the binding of the NO dimer.

C. Estimating geometrical errors

Obtaining energy gradients (for e.g., forces) is challenging with methods from cWFT such as CCSD(T) and even for codes where this is possible, it would be highly expensive. As such, it is common to use geometries generated by a lower level of theory such as DFT. There is thus an error in the resulting E_{ads} that arises from the use of a DFT geometry. Besides this geometrical error in E_{ads}, there is also an additional error because we use DFT to calculate E_{rlx} in Eq. 2; specifically with the DFA used to generate the geometry. There is an additional error associated with using this DFT value of E_{rlx} and we aim to capture the combination of these two errors within an error term dubbed ϵ_{geom}.

This ϵ_{geom} error can be estimated from the ensemble of DFAs. The application of a method, whether another DFA or one from cWFT, on the revPBE-D4 geometry can be denoted as Method//revPBE-D4 and the resulting approximate adsorption energy $E_{\text{ads}}^{\text{approx}}$ can be defined as:

$$\begin{aligned}E_{\text{ads}}^{\text{approx}}[\text{Method//revPBE-D4}] =& E_{\text{int}}[\text{Method//revPBE-D4}]+\\ & E_{\text{rlx}}[\text{revPBE-D4//revPBE-D4}]+\\ & \left(E_{\text{coh}}[\text{Method//revPBE-D4}] + E_{\text{conf}}[\text{Method//revPBE-D4}]\right),\end{aligned} \tag{6}$$

where E_{coh} and E_{conf} are included for the systems that have those terms, discussed in Section 7. The true adsorption energy $E_{\text{ads}}^{\text{true}}$ corresponds to the E_{ads} evaluated with the method with its corresponding geometry (i.e., Method//Method) and it is the quantity that we ultimately aim to approximate with $E_{\text{ads}}^{\text{approx}}$.

We chose revPBE-D4 as the functional to generate the geometries for subsequent E_{int} with the SKZCAM protocol because we find that it provides a low error on $E_{\text{ads}}^{\text{approx}}$ relative to $E_{\text{ads}}^{\text{true}}$ when evaluated across the other functionals in the ensemble. In Fig. 14, we have compared the 6 DFAs within the ensemble for their performance in reproducing $E_{\text{ads}}^{\text{true}}$. The chosen systems highlight a range of binding and surfaces and revPBE-D4 performs well across all the adsorbate–surface systems, with an MAD that are all less than 5% of $E_{\text{ads}}^{\text{true}}$. We note that the only system where we have chosen not to use the revPBE-D4 geometry is NO dimer as a hybrid such as B3LYP-D2[Ne] predicts the correct ground state and does not strongly overbind its cohesive energy E_{coh} like the GGAs [104].

In Tables 24 and 25, we have computed $E_{\text{ads}}^{\text{approx}}$ (including the corresponding E_{int}) and $E_{\text{ads}}^{\text{true}}$ across the entire DFT ensemble for the monomers adsorbed on MgO(001) and TiO_2 surfaces respectively. We take ϵ_{geom} to be 2 times the root mean squared error (2RMSE) of $E_{\text{ads}}^{\text{approx}}$

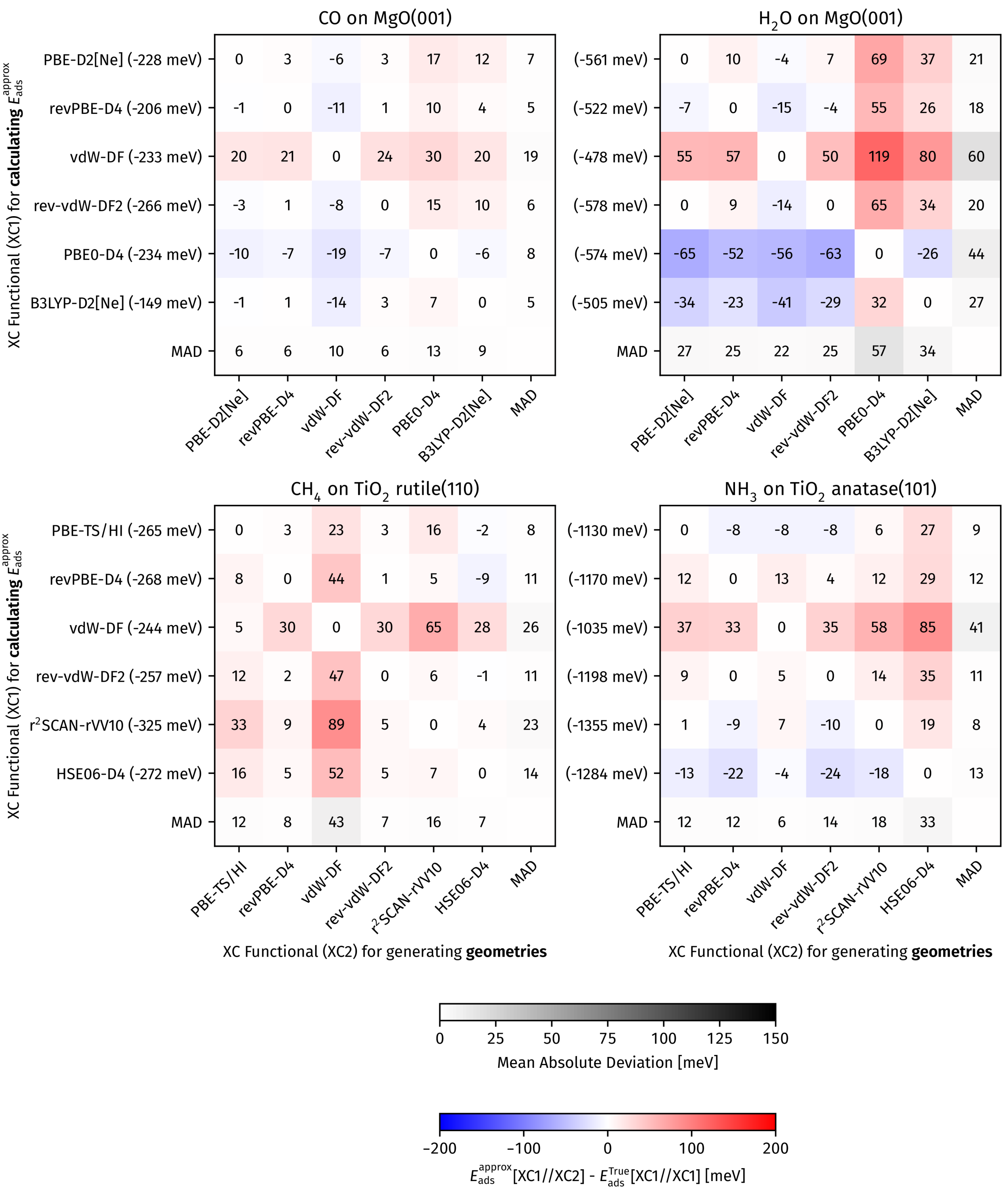

FIG. 14: Estimating the errors for using geometries generated by a DFA. The CO on MgO(001), H_2O on MgO(001), CH_4 on TiO_2 rutile(110) and NH_3 on TiO_2 anatase(101) adsorbate–surface systems were used as illustration. For the geometry generated by each DFA (on the x-axis), an approximate $E_{\text{ads}}^{\text{approx}}$ is calculated for the DFT ensemble along each column as defined in Eq. 6. This is compared to the true $E_{\text{ads}}^{\text{true}}$ from using the corresponding geometry of each DFA. The difference between $E_{\text{ads}}^{\text{approx}}$ and $E_{\text{ads}}^{\text{true}}$ is plotted, with a corresponding mean absolute deviation for each DFA's geometry is given in the bottom row.

against E_{ads}^{true} for the DFAs in the ensemble, excluding revPBE-D4 (which should have no error by definition). Assuming an even/normal distribution around E_{ads}^{true} from using the revPBE-D4 geometry for E_{ads}^{approx} , this error choice gives a 95% confidence interval on the final estimate.

We also compute this error for the monolayer and clusters on MgO in Table 26, where an additional cohesive energy term E_{coh} has evaluated with the corresponding DFA on the revPBE-D4 geometry. Table 26 also includes chemisorbed CO_2 on MgO(001), where there is an additional E_{conf} term to estimate E_{ads}^{approx} and obtain ϵ_{geom}.

TABLE 24: For the monomers on the MgO(001) surface, we estimate the errors for using the revPBE-D4 geometry and E_{rlx} in the final E_{ads} of the autoSKZCAM protocol using an ensemble of 6 different DFAs. The errors are calculated as the difference between the true E_{ads}^{true} (using the appropriate DFA) and the approximated E_{ads}^{approx} using the revPBE-D4 geometry and E_{rlx}.

		CH_4	C_2H_6	CO	C_6H_6	Parallel N_2O	Tilted N_2O	Vertical-Hollow NO	Vertical-Mg NO	Bent-Bridge NO	Bent-Mg NO	Bent-O NO	Monomer H_2O	Tilted CH_3OH	Parallel CH_3OH	NH_3	Physisorbed CO_2
PBE-D2[Ne]	E_{int}	-115	-157	-233	-278	-180	-137	-263	-170	-353	-207	-242	-666	-735	-460	-617	-238
	E_{rlx}	2	1	8	26	3	10	29	9	51	6	39	114	145	46	89	14
	E_{ads}^{approx}	-114	-156	-224	-252	-177	-127	-234	-161	-302	-201	-203	-552	-590	-414	-528	-225
	E_{ads}^{true}	-115	-158	-228	-261	-179	-132	-184	-162	-301	-203	-196	-561	-599	-417	-540	-227
	Error	2	2	3	9	2	5	-50	1	-1	2	-7	10	9	3	12	2
revPBE-D4	E_{int}	-143	-219	-215	-565	-192	-133	-184	-142	-346	-184	-234	-637	-770	-502	-636	-268
	E_{rlx}	2	1	8	26	3	10	29	9	51	6	39	114	145	46	89	14
	E_{ads}^{approx}	-142	-218	-206	-538	-189	-123	-154	-133	-294	-178	-195	-522	-625	-457	-547	-254
	E_{ads}^{true}	-142	-218	-206	-538	-189	-123	-154	-133	-294	-178	-195	-522	-625	-457	-547	-254
	Error	0	0	0	0	0	0	0	0	0	0	0	0	0	0	0	0
vdW-DF	E_{int}	-137	-197	-221	-391	-246	-181	-174	-179	-306	-227	-221	-535	-643	-405	-590	-258
	E_{rlx}	2	1	8	26	3	10	29	9	51	6	39	114	145	46	89	14
	E_{ads}^{approx}	-135	-196	-212	-365	-242	-171	-145	-170	-255	-221	-182	-421	-498	-360	-502	-245
	E_{ads}^{true}	-156	-233	-233	-466	-261	-220	-187	-191	-300	-243	-224	-478	-576	-408	-543	-277

Continued on next page

TABLE 24: (continued)

	Error	21	37	21	101	19	50	42	21	45	22	42	57	78	48	41	33
rev-vdW-DF2	E_{int}	-141	-207	-273	-460	-247	-192	-293	-254	-411	-260	-290	-683	-794	-490	-671	-303
	E_{rlx}	2	1	8	26	3	10	29	9	51	6	39	114	145	46	89	14
	$E_{\mathrm{ads}}^{\mathrm{approx}}$	-140	-206	-265	-433	-244	-182	-264	-245	-360	-254	-251	-568	-649	-444	-583	-289
	$E_{\mathrm{ads}}^{\mathrm{true}}$	-140	-207	-266	-449	-246	-183	-227	-208	-358	-255	-247	-578	-663	-451	-584	-288
	Error	0	1	1	16	2	1	-37	-37	-2	0	-5	9	14	6	1	-2
PBE0-D4	E_{int}	-159	-227	-250	-523	-246	-166	-103	-126	-259	-171	-167	-741	-853	-536	-701	-325
	E_{rlx}	2	1	8	26	3	10	29	9	51	6	39	114	145	46	89	14
	$E_{\mathrm{ads}}^{\mathrm{approx}}$	-158	-226	-241	-497	-243	-156	-74	-117	-208	-165	-128	-626	-708	-491	-613	-312
	$E_{\mathrm{ads}}^{\mathrm{true}}$	-158	-229	-234	-521	-251	-160	-102	-123	-192	-172	-123	-574	-659	-469	-576	-304
	Error	0	4	-7	24	8	4	28	6	-16	6	-5	-52	-49	-22	-37	-7
B3LYP-D2[Ne]	E_{int}	-89	-127	-156	-210	-168	-98	-37	-66	-171	-108	-100	-643	-714	-427	-599	-230
	E_{rlx}	2	1	8	26	3	10	29	9	51	6	39	114	145	46	89	14
	$E_{\mathrm{ads}}^{\mathrm{approx}}$	-87	-126	-148	-184	-164	-88	-7	-57	-120	-102	-61	-528	-569	-382	-510	-216
	$E_{\mathrm{ads}}^{\mathrm{true}}$	-88	-131	-149	-219	-173	-101	-70	-76	-136	-118	-81	-505	-552	-371	-492	-215
	Error	1	5	1	35	8	13	62	18	16	16	20	-23	-17	-11	-18	-1
	2RMSE	19	34	20	100	20	46	91	42	45	25	42	73	85	49	53	30

TABLE 25: For the monomers on the TiO_2 rutile(110) and anatase(101) surfaces, we estimate the errors for using the revPBE-D4 geometry and E_{rlx} in the final E_{ads} of the autoSKZCAM protocol using an ensemble of 6 different DFAs. The errors are calculated as the difference between the true E_{ads}^{true} (using the appropriate DFA) and the approximated E_{ads}^{approx} using the revPBE-D4 geometry and E_{rlx}.

		CH_4 on TiO_2 rutile(110)	Parallel CO_2 on TiO_2 rutile(110)	Tilted CO_2 on TiO_2 rutile(110)	H_2O on TiO_2 rutile(110)	CH_3OH on TiO_2 rutile(110)	H_2O on TiO_2 anatase(101)	NH_3 on TiO_2 anatase(101)
PBE-TS/HI	E_{int}	-283	-333	-400	-1236	-1560	-1108	-1351
	E_{rlx}	22	14	50	238	302	225	212
	E_{ads}^{approx}	-262	-318	-350	-998	-1258	-883	-1138
	E_{ads}^{true}	-265	-329	-366	-988	-1231	-860	-1130
	Error	-3	-11	-16	10	27	23	8
revPBE-D4	E_{int}	-289	-402	-441	-1214	-1549	-1096	-1382
	E_{rlx}	22	14	50	238	302	225	212
	E_{ads}^{approx}	-268	-388	-390	-976	-1247	-871	-1170
	E_{ads}^{true}	-268	-388	-390	-976	-1247	-871	-1170
	Error	0	0	0	0	0	0	0
vdW-DF	E_{int}	-235	-366	-437	-1080	-1370	-969	-1214
	E_{rlx}	22	14	50	238	302	225	212
	E_{ads}^{approx}	-214	-352	-387	-841	-1068	-744	-1002
	E_{ads}^{true}	-244	-381	-395	-849	-1090	-759	-1035
	Error	-30	-28	-8	-8	-23	-15	-33
rev-vdW-DF2	E_{int}	-276	-409	-472	-1300	-1619	-1171	-1410
	E_{rlx}	22	14	50	238	302	225	212
	E_{ads}^{approx}	-255	-395	-422	-1062	-1317	-946	-1198

Continued on next page

TABLE 25: (continued)

	E_{ads}^{true}	-257	-402	-430	-1050	-1312	-933	-1198
	Error	-2	-7	-7	12	5	13	0
r^2SCAN-rVV10	E_{int}	-338	-533	-584	-1501	-1832	-1367	-1577
	E_{rlx}	22	14	50	238	302	225	212
	E_{ads}^{approx}	-316	-519	-533	-1263	-1529	-1142	-1364
	E_{ads}^{true}	-325	-537	-557	-1232	-1491	-1124	-1355
	Error	-9	-18	-23	31	39	18	9
HSE06-D4	E_{int}	-288	-406	-481	-1368	-1688	-1229	-1518
	E_{rlx}	22	14	50	238	302	225	212
	E_{ads}^{approx}	-267	-392	-431	-1130	-1386	-1004	-1306
	E_{ads}^{true}	-272	-400	-447	-1092	-1333	-967	-1284
	Error	-5	-8	-16	38	52	36	22
	2RMSE	29	33	31	46	66	45	37

TABLE 26: For the clusters and monolayer systems as well as chemisorbed CO_2 on MgO(001) surface, we estimate the errors for using the DFT geometry and E_{rlx} in the final E_{ads} of the autoSKZCAM protocol using an ensemble of 6 different DFAs. The errors are calculated as the difference between the true E_{ads}^{true} (using the appropriate DFA) and the approximated E_{ads}^{approx} using the revPBE-D4 geometry and E_{rlx}. For the NO dimer, this is done with respect to the B3LYP-D2[N2] geometry and E_{rlx}. There is an additional cohesive energy E_{coh} term for the monolayer and cluster systems. For the chemisorbed CO_2, there is an additional conformational energy E_{conf} term.

		Monolayer CH_4	Monolayer C_2H_6	Dimer NO	Tetramer H_2O	Tetramer CH_3OH	Chemisorbed CO_2
PBE-D2[Ne]	E_{int}	-114	-142	-340	-397	-416	-2993
	E_{rlx}	1	4	26	52	68	663
	E_{coh} or E_{conf}	-40	-106	-234	-309	-380	1841

Continued on next page

TABLE 26: (continued)

	E_{ads}^{approx}	-153	-245	-548	-654	-729	-489
	E_{ads}^{true}	-153	-248	-551	-660	-733	-518
	Error	0	-3	-3	-6	-5	-29
revPBE-D4	E_{int}	-142	-207	-351	-404	-483	-2981
	E_{rlx}	1	4	26	52	68	663
	E_{coh} or E_{conf}	-31	-48	-186	-261	-328	1797
	E_{ads}^{approx}	-171	-251	-511	-613	-743	-521
	E_{ads}^{true}	-171	-251	-514	-613	-743	-521
	Error	0	0	-3	0	0	0
vdW-DF	E_{int}	-136	-200	-337	-376	-447	-2638
	E_{rlx}	1	4	26	52	68	663
	E_{coh} or E_{conf}	-58	-67	-145	-240	-291	1763
	E_{ads}^{approx}	-192	-263	-457	-564	-670	-212
	E_{ads}^{true}	-219	-319	-478	-597	-719	-361
	Error	-27	-55	-21	-33	-48	-149
rev-vdW-DF2	E_{int}	-140	-197	-398	-452	-519	-3082
	E_{rlx}	1	4	26	52	68	663
	E_{coh} or E_{conf}	-43	-83	-276	-289	-357	1836
	E_{ads}^{approx}	-181	-277	-648	-689	-809	-583
	E_{ads}^{true}	-182	-282	-654	-693	-811	-605
	Error	-1	-5	-5	-4	-2	-21
PBE0-D4	E_{int}	-158	-211	-290	-465	-526	-3713
	E_{rlx}	1	4	26	52	68	663
	E_{coh} or E_{conf}	-34	-64	62	-302	-367	2153
	E_{ads}^{approx}	-191	-271	-202	-715	-825	-898
	E_{ads}^{true}	-193	-274	-219	-692	-795	-764
	Error	-3	-2	-17	23	30	134
B3LYP-D2[Ne]	E_{int}	-87	-111	-231	-400	-429	-3250
	E_{rlx}	1	4	26	52	68	663

Continued on next page

TABLE 26: (continued)

E_{coh} or E_{conf}	-37	-124	63	-303	-371	2075
$E_{\text{ads}}^{\text{approx}}$	-124	-231	-143	-651	-732	-512
$E_{\text{ads}}^{\text{true}}$	-127	-238	-143	-643	-717	-460
Error	-4	-7	0	8	16	52
2RMSE	25	50	25	38	53	188

In general, we find that ϵ_{geom} is relatively small, being less than 40 meV for the majority of systems. For the monomers on MgO, the ϵ_{geom} range is between 20−100 meV, while this error range lowers to 29−66 meV for the TiO_2 surfaces. We find that this geometrical error is particularly dependent on the strength of the binding, going from 20 meV for CO on MgO - a system with weak physisorption to 66 meV for CH_3OH on TiO_2 rutile(110), a system with much stronger adsorption behaviour. In particular, the largest error is found for the chemisorbed CO_2 system with a value of 188 meV, arising from the large change to the surface and molecule electronic structure and geometry due to charge transfer.

D. Zero-point vibrational and enthalpic contributions

The DFT ensemble was also used to calculate the zero-point vibrational E_{ZPV} and thermal E_{T} contributions to the adsorption enthalpy H_{ads}. As both E_{ZPV} and E_{T} can be computed from vibrational modes, we will particular discuss overall enthalpic contributions: $\Delta H = E_{\text{ZPV}} + E_{\text{T}} - RT$, to add onto E_{ads} to make H_{ads} We utilised the rigid-rotor quasi-harmonic approximation (quasi-RRHO) to compute these terms based on the vibrational frequencies computed by DFT. These were first proposed by Grimme *et al.* [119] for entropies and then adapted for enthalpies by Li *et al.* [120]. Within the simple rigid-rotor harmonic oscillator (RRHO) models, the contribution of each vibrational mode i with frequency ν_i contributes $V_i^{RRHO} = \frac{1}{2}h\nu_i + \frac{h\nu_i}{1+e^{h\nu_i/k_BT}}$ to ΔH (including the contribution to E_{ZPV} in the first term and E_{T} in the second term). One deficiency of this model is that it is wrong for the zero-frequency (translation + rotation) modes as it predicts a k_BT contribution to ΔH rather than $\frac{1}{2}k_BT$. The quasi-RRHO fixes this issue by creating an interpolation between the RRHO model and the free rotor model (whereby each mode contributes $\frac{1}{2}k_BT$) at low frequencies:

$$V_i^{\text{quasi-RRHO}} = \omega(\nu_i) \times V_i^{\text{RRHO}} + (1 - \omega(\nu_i)) \times \frac{1}{2}k_BT, \quad (7)$$

$$\omega(\nu_i) = \frac{1}{1+(\nu_0/\nu_i)^4} \quad (8)$$

where we have set the interpolation to start at around $\nu_0 = 100$ cm^{-1}.

We have calculated the vibrational frequencies of the molecule (both on the surface and in the gas-phase) to calculate the ΔH contribution to H_{ads}. This is shown in Table 27, where we have computed E_{ZPV}, E_{T} and ΔH using four DFAs from the ensemble. For the MgO surface, the four DFAs were: XC 1 = PBE-D2[Ne], XC 2 = revPBE-D4, XC 3 = vdW-DF, XC 4 = rev-vdW-DF2. For the TiO_2 surfaces, the DFAs selected were: XC 1 = PBE-TS/HI, XC 2 = revPBE-D4, XC 3 = vdW-DF, XC 4 = rev-vdW-DF2. The final ΔH contribution was taken to be the average of the four functionals with the error estimated to be the 2σ standard deviation of the four estimates to ensure 95% confidence interval in the estimate. It can be seen that the ΔH contribution, relative to E_{ads} is overall small to H_{ads}, ranging from -10 to 75 meV. Moreover the error estimates are also small, ranging from 1 to 15 meV.

We expect nuclear quantum [121] contributions to ΔH to be small while anharmonic effects can have a potential effect for weakly binding molecules such as CH_4 on MgO(001) [122]. This effect is well-captured by the error bar, where CH_4 on MgO(001) gives a larger error bar on ΔH than the stronger binding systems. Another source of error is the freezing of surface vibrational modes when calculating ΔH. In Table 28, we have calculated ΔH for a select number of systems which include the surface degrees of freedom in the top two layers of MgO(001). Overall, there is negligible change of maximum 2 meV for the majority of systems. The only system where there is a significant effect is for chemisorbed CO_2 where it changes from 18 ± 5 meV to 31 ± 5 meV, due to changes in the surface electronic structure from charge transfer. In our final H_{ads} analysis, we will include the surface degrees of freedom for CO_2 on MgO(001) only, while neglecting it for all other systems.

E. Dissociation energy for the H_2O and CH_3OH clusters

As discussed in Section 1 A, we use the autoSKZCAM framework to obtain the adsorption enthalpy for a molecularly adsorbed (tetramer) cluster of CH_3OH and H_2O. The lowest energy geometry involves partial dissocation of the CH_3OH and H_2O clusters. In our final H_{ads} for these two systems, we compute an additional term E_{diss} which accounts for the (electronic) energetic stabilisation to form the partially dissociated cluster. This is performed using the DFT ensemble – using the revPBE-D4 geometry – shown in Table 29 for the 6 DFAs, where we obtain an average and calculate the error as the 2σ standard deviation. We have also computed the

TABLE 27: The zero-point vibrational energy (E_{ZPV}), thermal energy (E_{therm}), and overall enthalpy (ΔH) contributions (in meV) to H_{ads} in all studied systems for an ensemble of 4 DFAs. Errors in ΔH are given as the 2σ standard deviation.

	CH_4 on MgO(001)	Monolayer CH_4 on MgO(001)	C_2H_6 on MgO(001)	Monolayer C_2H_6 on MgO(001)	CO on MgO(001)	C_6H_6 on MgO(001)	Parallel N_2O on MgO(001)	Tilted N_2O on MgO(001)	Vertical-Hollow NO on MgO(001)	Vertical-Mg NO on MgO(001)	Bent-Bridge NO on MgO(001)	Bent-Mg NO on MgO(001)	Bent-O NO on MgO(001)	Dimer NO on MgO(001)
Temperature [K]	47	47	75	75	61	162	77	77	80	80	80	80	80	80
RT [meV]	4	4	6	6	5	14	7	7	7	7	7	7	7	7
E_{ZPV} [XC 1]	29	29	12	17	31	1	1	3	18	13	23	11	10	63
E_T [XC 1]	-5	-5	-2	-4	-6	2	-1	0	-5	-3	-6	-3	-4	-10
ΔH [XC 1]	20	20	3	7	20	-12	-6	-4	7	3	10	1	0	47
E_{ZPV} [XC 2]	23	28	13	37	28	9	2	6	22	14	26	13	11	64
E_T [XC 2]	-4	-5	-2	-5	-5	2	-1	-1	-5	-4	-6	-3	-4	-10
ΔH [XC 2]	15	20	4	26	18	-3	-5	-2	10	4	12	3	0	47
E_{ZPV} [XC 3]	10	10	0	28	28	-1	0	0	12	10	18	9	7	58
E_T [XC 3]	-2	-2	0	-5	-5	2	0	0	-3	-3	-5	-2	-3	-9
ΔH [XC 3]	3	4	-7	17	17	-14	-7	-7	2	0	6	0	-3	42
E_{ZPV} [XC 4]	15	17	4	22	32	-1	1	4	19	14	25	12	10	65
E_T [XC 4]	-4	-4	-1	-5	-6	2	-1	0	-5	-4	-6	-3	-4	-10
ΔH [XC 4]	8	8	-4	10	21	-13	-6	-3	7	4	12	2	0	48
Final ΔH	11	13	-1	15	19	-10	-6	-4	7	2	10	1	-1	46
Error	13	14	10	14	3	9	1	4	6	3	5	3	3	5

	Monomer H_2O on MgO(001)	Tetramer H_2O on MgO(001)	Tilted CH_3OH on MgO(001)	Parallel CH_3OH on MgO(001)	Tetramer CH_3OH on MgO(001)	NH_3 on MgO(001)	Physisorbed CO_2 on MgO(001)	Chemisorbed CO_2 on MgO(001)	CH_4 on TiO_2 rutile(110)	Parallel CO_2 on TiO_2 rutile(110)	Tilted CO_2 on TiO_2 rutile(110)	H_2O on TiO_2 rutile(110)	CH_3OH on TiO_2 rutile(110)	H_2O on TiO_2 anatase(101)	NH_3 on TiO_2 anatase(101)
Temperature [K]	203	235	286	286	286	160	120	230	85	177	177	303	370	257	410
RT [meV]	17	20	25	25	25	14	10	20	7	15	15	26	32	22	35
E_{ZPV} [XC 1]	84	98	36	22	61	72	2	47	17	7	9	110	68	113	111
E_T [XC 1]	-22	-19	3	1	1	-13	0	-8	-3	3	3	-18	10	-18	-2
ΔH [XC 1]	45	58	14	-1	38	46	-8	19	6	-5	-3	66	46	73	74
E_{ZPV} [XC 2]	85	100	38	24	63	72	1	47	25	9	12	111	74	112	115
E_T [XC 2]	-21	-19	3	1	1	-12	0	-8	-4	3	3	-15	9	-15	1
ΔH [XC 2]	47	61	17	0	39	46	-9	19	13	-3	0	69	51	75	80
E_{ZPV} [XC 3]	84	97	41	24	62	69	-2	44	6	5	7	96	71	90	105
E_T [XC 3]	-18	-16	5	1	5	-12	1	-8	-1	3	3	-7	13	-9	3
ΔH [XC 3]	48	60	21	1	43	43	-11	16	-3	-7	-5	63	52	59	72
E_{ZPV} [XC 4]	85	100	37	21	61	68	0	48	19	11	13	108	67	107	107
E_T [XC 4]	-21	-20	3	1	0	-13	0	-9	-5	3	3	-17	9	-15	0
ΔH [XC 4]	46	60	15	-3	37	42	-10	19	7	-2	0	64	44	70	71
Final ΔH	47	60	17	-1	39	44	-10	18	6	-4	-2	65	48	69	75
Error	3	2	6	3	5	3	2	2	11	4	4	5	7	13	7

TABLE 28: Comparing the effect of only the molecule vibrational degrees of freedom and the inclusion of surface degrees of freedom on the enthalpy (ΔH) contribution (in meV) to the adsorption enthalpy for a select few molecules adsorbed on MgO(001). ΔH is calculated as the mean from an ensemble of 4 DFAs (neglecting the hybrid DFAs) with 2σ error included.

	CO	H_2O Monomer	CO_2 Physisorbed	CO_2 Chemisorbed
Molecule	19 ± 6	47 ± 5	-10 ± 5	18 ± 5
Molecule+Surface	19 ± 1	48 ± 5	-8 ± 2	31 ± 3

TABLE 29: The dissociation energy E_{diss} (in meV) for the CH_3OH and H_2O tetramer calculated for the DFT ensemble. This is defined as the energy difference between the dissociated and molecular configurations of the tetramer.

	CH_3OH Tetramer	H_2O Tetramer
PBE-D2[Ne]	-76	-89
revPBE-D4	-78	-81
vdW-DF	-48	-52
rev-vdW-DF2	-65	-75
PBE0-D4	-80	-83
B3LYP-D2[Ne]	-81	-98
Average	-71	-80
Error	24	29
Final	-71 ± 24	-79 ± 29

enthalpic difference (-31 meV and -18 meV for H_2O and CH_3OH) between the dissociated and molecular configurations (with geometries taken from revPBE-D4) and added this contribution to the original ΔH calculated for the molecular configuration in Table 27; this leads to a final ΔH of 42 ± 2 meV and 8 ± 5 meV for the dissociated configuration of H_2O and CH_3OH respectively, as shown in Table 30.

9. FINAL AUTOSKZCAM ESTIMATES

In Table 30, we show the terms which make up the final E_{ads} and H_{ads} estimates in our autoSKZCAM framework. Robust error bars have been estimated which aim to encapsulate the major sources of errors (to at least a 95% confidence interval) within this estimate with respect to a fully converged CCSD(T) estimate. The $E_{\text{int}}^{\text{SKZCAM}}$ error estimate covers the potential finite-size errors from utilising the SKZCAM protocol in Section 6, while ϵ_{geom} (discussed in Section 8) covers the error from computing $E_{\text{rlx}}^{\text{DFT}}$ with DFT and the error from using a DFT geometry in all the individual terms of E_{ads}. We reach a final error estimate as the root squared sum of the errors within the individual terms. As seen in Table 30, ϵ_{geom} is in general the largest source of error out of all of the terms and serves as the major term to target for future improvements.

TABLE 30: The terms which make up the autoSKZCAM E_{ads} and H_{ads} estimates (in meV). E_{ads} is the sum of E_{int}^{SKZCAM}, E_{rlx}^{DFT}, $E_{coh}^{CCSD(T)}$ and $E_{conf}^{CCSD(T)}$, where the last two terms are only included for a subset of systems in Section 7. Errors due to E_{rlx}^{DFT} and from using the revPBE-D4 geometry is encapsulated in ϵ_{geom} using the ensemble of DFAs. ΔH is also calculated from the DFT ensemble.

	E_{int}^{SKZCAM}	E_{rlx}^{DFT}	$E_{coh}^{CCSD(T)}$	$E_{conf}^{CCSD(T)}$	ϵ_{geom}	$E_{diss.}^{DFT}$	$E_{ads}^{autoSKZCAM}$	ΔH^{DFT}	$H_{ads}^{autoSKZCAM}$
CH_4 on MgO(001)	-122 ± 2	2	-	-	19	-	-120 ± 19	11 ± 13	-109 ± 23
Monolayer CH_4 on MgO(001)	-121 ± 3	1	-25	-	25	-	-145 ± 25	13 ± 14	-132 ± 28
C_2H_6 on MgO(001)	-175 ± 4	1	-	-	34	-	-174 ± 34	-1 ± 10	-175 ± 35
Monolayer C_2H_6 on MgO(001)	-161 ± 3	4	-61	-	50	-	-218 ± 50	15 ± 14	-203 ± 52
CO on MgO(001)	-207 ± 4	8	-	-	20	-	-198 ± 20	19 ± 3	-180 ± 20
C_6H_6 on MgO(001)	-446 ± 9	26	-	-	100	-	-420 ± 100	-10 ± 9	-430 ± 100
Parallel N_2O on MgO(001)	-256 ± 3	3	-	-	20	-	-253 ± 20	-6 ± 1	-259 ± 20
Tilted N_2O on MgO(001)	-168 ± 4	10	-	-	46	-	-157 ± 46	-4 ± 4	-161 ± 47
Vertical-Hollow NO on MgO(001)	32 ± 4	29	-	-	91	-	61 ± 91	7 ± 6	68 ± 91
Vertical-Mg NO on MgO(001)	-62 ± 5	9	-	-	42	-	-53 ± 42	2 ± 3	-50 ± 42
Bent-Bridge NO on MgO(001)	-62 ± 8	51	-	-	45	-	-10 ± 45	10 ± 5	0 ± 46
Bent-Mg NO on MgO(001)	-126 ± 5	6	-	-	25	-	-120 ± 26	1 ± 3	-119 ± 26
Bent-O NO on MgO(001)	-3 ± 7	39	-	-	42	-	36 ± 43	-1 ± 3	35 ± 43
Dimer NO on MgO(001)	-246 ± 3	42	-75	-	59	-	-278 ± 59	46 ± 5	-232 ± 59
Monomer H_2O on MgO(001)	-703 ± 4	114	-	-	73	-	-588 ± 73	47 ± 3	-542 ± 73
Dissociated Tetramer H_2O on MgO(001)	-463 ± 7	52	-281	-	38	-80 ± 29	-772 ± 48	42 ± 2	-730 ± 48
Tilted CH_3OH on MgO(001)	-787 ± 5	145	-	-	85	-	-642 ± 86	17 ± 6	-625 ± 86
Parallel CH_3OH on MgO(001)	-506 ± 1	46	-	-	49	-	-461 ± 49	-1 ± 3	-461 ± 49
Dissociated Tetramer CH_3OH on MgO(001)	-511 ± 6	68	-336	-	53	-71 ± 24	-851 ± 59	8 ± 5	-843 ± 59
NH_3 on MgO(001)	-657 ± 9	89	-	-	53	-	-568 ± 53	44 ± 3	-524 ± 54
Physisorbed CO_2 on MgO(001)	-308 ± 2	14	-	-	30	-	-294 ± 30	-10 ± 2	-304 ± 30
Chemisorbed CO_2 on MgO(001)	-3504 ± 32	663	-	2094	188	-	-747 ± 191	18 ± 2	-729 ± 191
CH_4 on TiO_2 rutile(110)	-269 ± 2	22	-	-	29	-	-247 ± 29	6 ± 11	-241 ± 31
Parallel CO_2 on TiO_2 rutile(110)	-410 ± 5	14	-	-	33	-	-396 ± 33	-4 ± 4	-400 ± 34
Tilted CO_2 on TiO_2 rutile(110)	-493 ± 7	50	-	-	31	-	-442 ± 31	-2 ± 4	-445 ± 32
H_2O on TiO_2 rutile(110)	-1310 ± 33	238	-	-	46	-	-1072 ± 57	65 ± 5	-1007 ± 57
CH_3OH on TiO_2 rutile(110)	-1634 ± 37	302	-	-	66	-	-1332 ± 76	48 ± 7	-1284 ± 76
H_2O on TiO_2 anatase(101)	-1208 ± 16	225	-	-	45	-	-983 ± 48	69 ± 13	-913 ± 50
NH_3 on TiO_2 anatase(101)	-1377 ± 18	212	-	-	37	-	-1165 ± 41	75 ± 7	-1090 ± 42

A. Validating autoSKZCAM error estimates

In this section, we validate our chosen procedure for estimating errors on H_{ads} in the autoSKZCAM framework, using the chemisorbed CO_2 on MgO(001) as an example due to the relatively large estimated error bars for this system. As seen in Table 30, the major source of error comes from ϵ_{geom} – the error for utilising DFT to generate the geometry to calculate the adsorption energy E_{ads}, encapsulating the interaction energy E_{int}, relaxation energy E_{rlx} and additional terms (e.g., E_{coh} and E_{conf}). To understand how well our error estimates cover the errors from utilising DFT to generate the geometry, we have calculated H_{ads} with the autoSKZCAM framework using the geometries from the 6 different density functional approximations in the DFT ensemble in Table 31. Our final H_{ads} estimate (in Table 30) of -729 ± 191 meV covers a range from -538,meV to -920 meV. The actual range of values for using the six different DFAs goes from -599 meV for PBE0-D4 to -840,meV for vdW-DF. There is overall a faithful (and conservative) representation of the range of possible values of H_{ads} in Table 31, with no values lying outside this error bar.

TABLE 31: Final H_{ads} (in meV) for CO_2 chemisorbed on MgO using various DFAs as the geometry within the subsequent autoSKZCAM framework treatment. The final H_{ads} is the sum of $E_{int}^{MP2,bulk}$, Δ_{CC}, Δ_{basis}, E_{rlx}^{DFT}, $E_{conf}^{CCSD(T)}$ and ΔH^{DFT} terms as described in Section 8.

	$E_{int}^{MP2,bulk}$	Δ_{CC}	Δ_{basis}	E_{rlx}^{DFT}	$E_{conf}^{CCSD(T)}$	ΔH^{DFT}	H_{ads}^{final}
PBE-D2[Ne]	-3358	-225	24	693	2085	18	-763
revPBE-D4	-3309	-220	25	663	2094	18	-729
vdW-DF	-3457	-228	23	704	2100	18	-840
rev-vdW-DF2	-3296	-223	24	676	2055	18	-746
PBE0-D4	-3223	-224	24	773	2033	18	-599
B3LYP-D2[Ne]	-3338	-227	24	810	2029	18	-685

10. AUTOMATION OF THE AUTOSKZCAM FRAMEWORK

The autoSKZCAM framework is freely available and open-source on Github (https://github.com/benshi97/ We have developed a set of functions that automates the entire SKZCAM protocol to generate the clusters and inputs to obtain an accurate interaction energy E_{int}. The remaining contributions to the adsorption enthalpy is calculated with the DFT ensemble and this can involve a significant number of calculations/book-keeping. We make extensive use of the QuAcc computational materials science workflow library [123] to manage these calculations. All of this can be achieved within a single Jupyter Notebook, requiring minimal user intervention.

A. QuAcc computational workflow details

The QuAcc workflow library contains (user-defined) 'recipes' that represent specific tasks (i.e., calculating the static energy or performing a geometry optimisation) and 'flows' which represent workflows that combine these recipes to e.g., generate slabs from the bulk or make an adsorbed surface geometry. This allows for the study of surfaces to be automated and efficiently dispatched to any computing environment. We make use of many of the pre-existing recipes and flows within QuAcc and as part of this work, we have also developed new recipes and flows to enable calculations using a DFT ensemble (or arbitrary number of DFAs).

In the left panel of Fig. 15, we highlight the processes executed within QuAcc to calculate the DFT ensemble contributions to H_{ads}. Starting from a unit cell, the `bulk_to_slab_flow` will generate the necessary surface termination and relax the surface to its equilibrium geometry. Subsequently, the adsorbate is added onto the surface within the `slab_to_ads_flow` to generate the relaxed adsorbate–surface complex. Currently the flow accepts placing the adsorbates on the "ontop", "bridge", "hollow" and "subsurface" sites if the position of the molecule is known or expected to follow chemical intuition. We also provide a short function to perform random structure search to produce starting geometries that can be relaxed to identify unbiased low-energy adsorbate–surface adsorption configurations.

From the candidate adsorbate–surface structure(s), the QuAcc workflow will then submit a series of `static_job` calculations which aims to calculate E_{ads}, E_{int} and E_{rlx} across the ensemble of DFAs. In particular, it will calculate both $E_{\mathrm{ads}}^{\mathrm{true}}$ and $E_{\mathrm{ads}}^{\mathrm{approx}}$ (see Section 8) to estimate the error with using a DFT geometry for E_{ads}. Subsequently the vibrational frequencies of the molecule (on the surface and in the gas-phase) are calculated in order to determine E_{ZPV} and E_{T} across

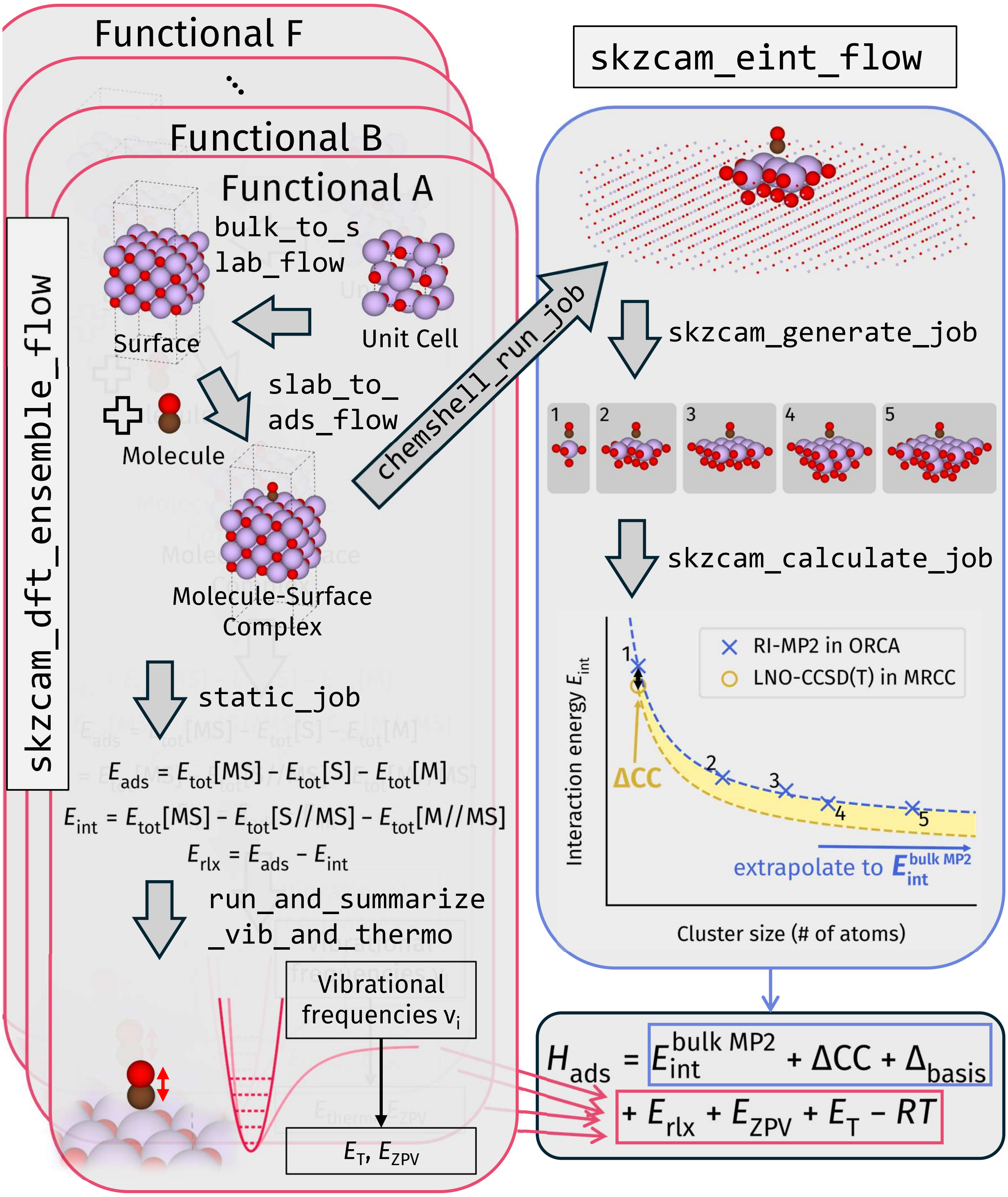

FIG. 15: The computational workflow for calculating an H_{ads} using the autoSKZCAM framework. It makes heavy use of the QuAcc computational materials science workflow library [123]. We make use of pre-existing 'flows' to generate the adsorbate–surface complex for the density functional approximations within the DFT ensemble and to subsequently calculate E_{ads}, E_{int} and E_{rlx} as well as E_{ZPV} and E_T. We have developed new Python modules to generate the series of clusters and inputs necessary to calculate a CCSD(T)-quality E_{int} with the SKZCAM protocol. The inputs can be either submitted directly on a computing cluster or managed through QuAcc. These terms are all combined to reach the final H_{ads} estimate.

the DFT ensemble.

B. Automated SKZCAM protocol

One of the key developments within the present work is the automatisation of the SKZCAM protocol, which significantly lowers its cost and requires minimal user intervention to operate. From an adsorbate-surface structure, either generated by the one of the DFAs in the DFT ensemble or taken from the literature, it will generate all of the inputs necessary to come to a final estimate of the E_{int}, with the choice of several (ONIOM) embedding layers within an intuitive interface.

We have developed a set of Python modules which runs through the key steps of the SKZCAM protocol. Firstly, it interfaces with the py-ChemShell [87] program to generate the necessary point charge environment using the `chemshell_run_job`. The generated output (a `.pun` file) is then read and used to generate the set of embedded clusters with `skzcam_calculate_job`. This will generate the approriate set of inputs (for the different levels of theory, basis set sizes and frozen core treatments, described in Section 6) that define all the individual calculations that need to be performed. At present, these inputs can be generated for either ORCA [68] or MRCC [69]. The inputs can be copied to the computing cluster of choice for the calculations to be performed. Alternatively, one can make use of the QuAcc computational workflow library to directly manage and submit the jobs. Once the MP2 and CCSD(T) calculations are complete, we provide analysis scripts to calculate the MP2 bulk limit $E_{\text{int}}^{\text{bulk MP2}}$, ΔCC contribution and further contributions for basis set Δ_{basis} and core contributions Δ_{core}.

11. ANALYSING EXPERIMENTAL ESTIMATES AND TECHNIQUES

Experimental techniques can obtain the adsorption enthalpy H_{ads} through methods such as single crystal adsorption calorimetry (SCAC), equilibrium adsorption isotherms (EAI) and temperature programmed desorption (TPD) experiments [3, 124]. SCAC are considered to provide the most reliable measurements of H_{ads} while EAI provides a straightforward means for measuring this quantity for reversible adsorption-desorption experiments using the Clausius-Clapeyron expression. Despite requiring more analysis than the other two methods, TPD experiments are by far the most common technique for measuring H_{ads} for metal-oxide surfaces [3] due to the simplicity and ready availability of TPD equipment.

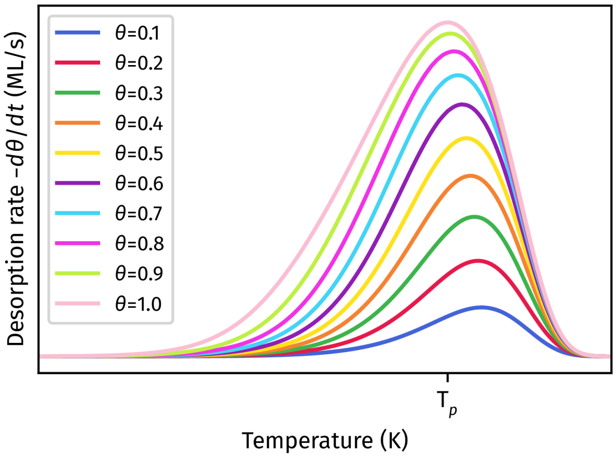

FIG. 16: A schematic of a typical temperature programmed desorption spectra as a function of concentration θ. Created with tools provided within Ref. 125.

In TPD experiments, the surface with preadsorbed molecules is heated at a constant heating rate. The rate of appearance of the gas desorbing from the surface is then monitored using a mass spectrometer. The desorption rate is measured as a function of the temperature to create plots such as shown in Fig. 16. The central equation for first order desorption TPD spectra is an Arrhenius type relationship which relates the desorption rate $-\frac{\mathrm{d}\theta}{\mathrm{d}t}$ to the activation energy E_{d} for desorption:

$$-\frac{\mathrm{d}\theta}{\mathrm{d}t} = \nu\theta \exp\left(-\frac{E_{\text{d}}}{RT_p}\right), \tag{9}$$

where ν is the pre-exponential factor, θ is the concentration and T_p is the temperature where the desorption rate is a maximum. One can then obtain E_{d} by inverting this first-order Wigner-Polanyi equation:

$$E_{\text{d}}(\theta) = -RT_p \ln\left(-\frac{\mathrm{d}\theta}{\mathrm{d}t}/(\nu\theta)\right). \tag{10}$$

From TPD curves plotted at various surface concentrations, the value of E_{d} and ν can also be obtained by finding the best fit to simulated TPDs. The errors on $\log(\nu)$ with this approach can be of the order of ± 2 [126].

The simplest method to determine E_{d} is through the Redhead equation [127], which relates the heating rate β and T_p to E_{d} by the following equation:

$$\frac{E_{\mathrm{d}}}{RT_p^2} = \left(\frac{\nu}{\beta}\right) \exp\left(-\frac{E_{\mathrm{d}}}{RT_p}\right), \tag{11}$$

Here, T_p and β are known but ν is normally estimated to be 10^{13}. Rearranging the equation and taking an empirical linear relationship between E_{d} and T_p gives the following relationship:

$$E_{\mathrm{d}} = RT_p \ln\left(\frac{\nu T_p}{\beta} - 3.64\right). \tag{12}$$

Thus, a ten order of magnitude change in ν is expected to change E_{d} by $\sim \ln(10)RT_p = 2.3RT_p$.

There can be three sources of discrepancy in reaching an experimental measurement of H_{ads}. The major source of error arises in the prescribed value of ν in the determination of E_{d} from the TPD spectra. This is not known but it can span between $\log(\nu) = 12$ and $\log(\nu) = 19$ [128] and most experiments will simply set $\log(\nu) = 13$, which can introduce large errors. Recently, Sellers and Campbell [128] have demonstrated a relationship between the entropy of the gas when adsorbed on a surface and in the gas phase, which has allowed for predictions of $\log(\nu)$ to a 2σ standard deviation of $\sim$1.72. The majority of experiments are taken from Campbell and Sellers [3], with H_{ads} using the predicted values of ν. We perform re-analysis of E_{d} with the predicted ν for systems not included within their work, indicated by systems where $\log(\nu) = 13.0 \rightarrow \ldots$ in Table 32. Second, there is also an error in E_{d} as it is not directly equal to H_{ads}. E_{d} is an activation energy, but its relationship to H_{ads} has differed between different studies. For example, the collection of experimental estimates [49, 61, 129, 130] re-analysed by Sauer and co-workers has used the relationship:

$$H_{\mathrm{ads}} = -E_{\mathrm{d}} + RT_p, \tag{13}$$

to compare to experiments, following on the approximate relation between the enthalpy and activation energy of reaction barriers [131]. On the other hand, the collection of work by Campbell [3, 132] and co-workers as well as others [133] have used the relationship:

$$H_{\mathrm{ads}} = -E_{\mathrm{d}} - \frac{1}{2}RT_p, \tag{14}$$

which was derived from relating the isosteric heat of adsorption (the negative of H_{ads}) from SCAC experiments to E_{d} in Ref. 134. In this work we opt to set H_{ads} to $-E_{\mathrm{d}}$, and add an RT_p error

contribution to account for the two potential directions which H_{ads} can point to with respect to E_{d}. The final source of error arises in differences in the concentration between simulation and experiment. For most work, for example from the re-analysis by Campbell and Sellers, a concentration dependence of E_{d} is provided and we opt to take the smallest concentration for the adsorption of monomers, while we take the highest concentration for the monolayers. For the clusters, we aim to take the average of the low and high concentration. We add the error bars as half the difference between the low and high concentration for the clusters. At the end, the final estimate of H_{ads} will have its error be the root squared sum of these three sources of errors.

TABLE 32: Experimental adsorption enthalpies (in meV) for the systems studied within this work.

Surface	Adsorbate	Temperature	log(ν)	H_{ads} (meV)	Error	Details
MgO(001)	CH_4	47	13.1 ± 2.0	-115	19	Dilute limit E_{d} estimate by Tait *et al.* [135, 136].
MgO(001)	C_2H_6	75	14.9 ± 2.0	-221	30	Dilute limit E_{d} estimate by Tait *et al.* [135, 136].
MgO(001)	CO	61	13.8 ± 1.6	-176	21	Average of the H_{ads} re-analysis by C&S for Refs. 126 and 30 at low coverage, with 0.5RT removed.
MgO(001)	N_2O	77	13.0 → 14.0 ± 2.0	-239	31	E_{d} measured by Lian *et al.* [137] with subsequent conversion to H_{ads}.
MgO(001)	C_6H_6	161.5	15.1 ± 1.6	-481	72	Average taken of H_{ads} re-analyzed by C&S between low and high coverage in Ref. 138, with 0.5RT removed.
MgO(001)	H_2O	203	-	-520	121	H_{ads} from Ferry *et al.* [11, 12, 130] estimated by subtracting lateral molecule-molecule interactions (-35.1 ± 9.6 kJ/mol) from the H_{ads} of H_2O monolayer on MgO, both obtained from LEED adsorption isotherms.
MgO(001)	NH_3	160	13.0 → 14.0 ± 2.0	-613	65	E_{d} measurement from Arthur *et al.* [139] with subsequent conversion to H_{ads}.
MgO(001)	Physisorbed CO_2	120	13.0 → 14.0 ± 2.0	-431	49	E_{d} measurement from Meixner *et al.* [13] with subsequent conversion to H_{ads}.
MgO(001)	Chemisorbed CO_2	230	13.0 → 14.0 ± 2.0	-664	125	E_{d} measurement from Chakradhar and Burghaus [14] as the average of the α and β peaks with subsequent conversion to H_{ads}.
MgO(001)	Monolayer CH_4	47	13.1 ± 2.0	-131	19	Monolayer E_{d} estimate by Tait *et al.* [135, 136].
MgO(001)	Monolayer C_2H_6	75	14.9 ± 2.0	-236	30	Monolayer E_{d} estimate by Tait *et al.* [135, 136].
MgO(001)	Cluster NO	79.5	14.0 ± 1.6	-232	31	Average taken of H_{ads} re-analyzed by C&S between low and high coverage in Ref. 30, with 0.5RT removed.
MgO(001)	Cluster H_2O	235	14.5 ± 1.6	-694	83	H_{ads} re-analysis by C&S for Ref. 140, with 0.5RT removed.
MgO(001)	Cluster CH_3OH	285.5	15.3 ± 1.6	-890	106	H_{ads} re-analysis by C&S for Ref. 2, with 0.5RT removed.
TiO_2 rutile(110)	CH_4	85	14.9 ± 2.0	-249	34	Low-coverage estimate of E_{d} by Chen *et al.* [141] with subsequent conversion to H_{ads}.
TiO_2 rutile(110)	CO_2	177	13.6 ± 2.0	-493	62	H_{ads} re-analysis by C&S for Ref. 142, with 0.5RT removed.
TiO_2 rutile(110)	H_2O	303	14.7 ± 1.6	-917	111	Average taken of H_{ads} re-analysis by C&S for Refs. 143 and 144 in low coverage limit
TiO_2 rutile(110)	CH_3OH	370	15.5 ± 1.6	-1197	130	H_{ads} re-analysis by C&S for Ref. 145, with 0.5RT removed.
TiO_2 anatase(101)	H_2O	257	14.6 ± 1.6	-786	90	H_{ads} re-analysis by C&S for Ref. 146, with 0.5RT removed.
TiO_2 anatase(101)	NH_3	410	13.3 ± 2.0	-1180	182	E_{d} estimate taken from Koust *et al.* [147] at the lowest studied coverage with subsequent conversion to H_{ads}, with 0.5RT removed.

12. COMPARISON OF H_{ads} BETWEEN AUTOSKZCAM AND EXPERIMENTS

With the autoSKZCAM approach, we have computed a final H_{ads} that aims to reach a converged CCSD(T)-level of accuracy. The error bars have been designed to capture the major sources of error to at least a 95% confidence interval: (1) finite size errors for calculating E_{int} with the SKZCAM protocol, (2) errors for using a DFT geometry and DFT E_{rlx} and (3) errors from using DFT to calculate zero-point vibrational and temperature contributions. Similarly, we have also analysed experimental TPD experiments to obtain H_{ads} estimates with reliable error bars that account for: (1) errors in the ν estimate, (2) errors between the measured Arrhenius activation energy and H_{ads} and (3) errors arising from concentration dependence.

We compare the autoSKZCAM predicted H_{ads} with experiment in Table 33 (and in Fig. 17). In particular, we calculate Δ_{min}, the smallest (absolute) deviation between autoSKZCAM and experiment within the limits of their error bars. If $\Delta_{min} = 0$, we expect the two estimates to be indistinguishable and to agree within their respective error bars. For all of the systems, we find the autoSKZCAM protocol to be indistinguishable from experiments.

In general, we find that the error bars from the autoSKZCAM framework are lower than experiment. For both the autoSKZCAM framework and experiment, the error bars increase when H_{ads} increases. For experiments, this arises because the errors are typically a function of RT_p and stronger binding correlates with a higher T_p. On the other hand, for the autoSKZCAM protocol, the errors rest mostly on ϵ_{geom} - the error arising from using a DFT geometry. A stronger binding typically means that the molecule has a stronger effect on the surface (electronic structure), hence leading to larger changes (and in turn E_{rlx}) to the geometry of the surface.

For several of the systems: CO_2, N_2O, NO, H_2O, CH_3OH on MgO(001) and CO_2 on TiO_2 rutile(110), we have studied multiple geometries using the autoSKZCAM framework in Section 1 and show in Table 33 the structure with the most negative H_{ads} value - the stable geometry we expect will be observed within experiments. Similarly, some systems have had several experimental H_{ads} estimates. For most of these systems, the experimental H_{ads} from different studies agree with each other, barring CO_2 on MgO(001), where two experiments have given H_{ads} that differ by more than 300 meV with one [13] suggesting a physisorbed structure while the other [14] predicting a chemisorbed structure. Our autoSKZCAM estimates have helped to shed further light on the discrepancies between these two experiments and provide evidence for the accuracy of one over the other, as discussed in Section 1 B.

We show in Table 34 the importance in using the correct analysis of experimental results,

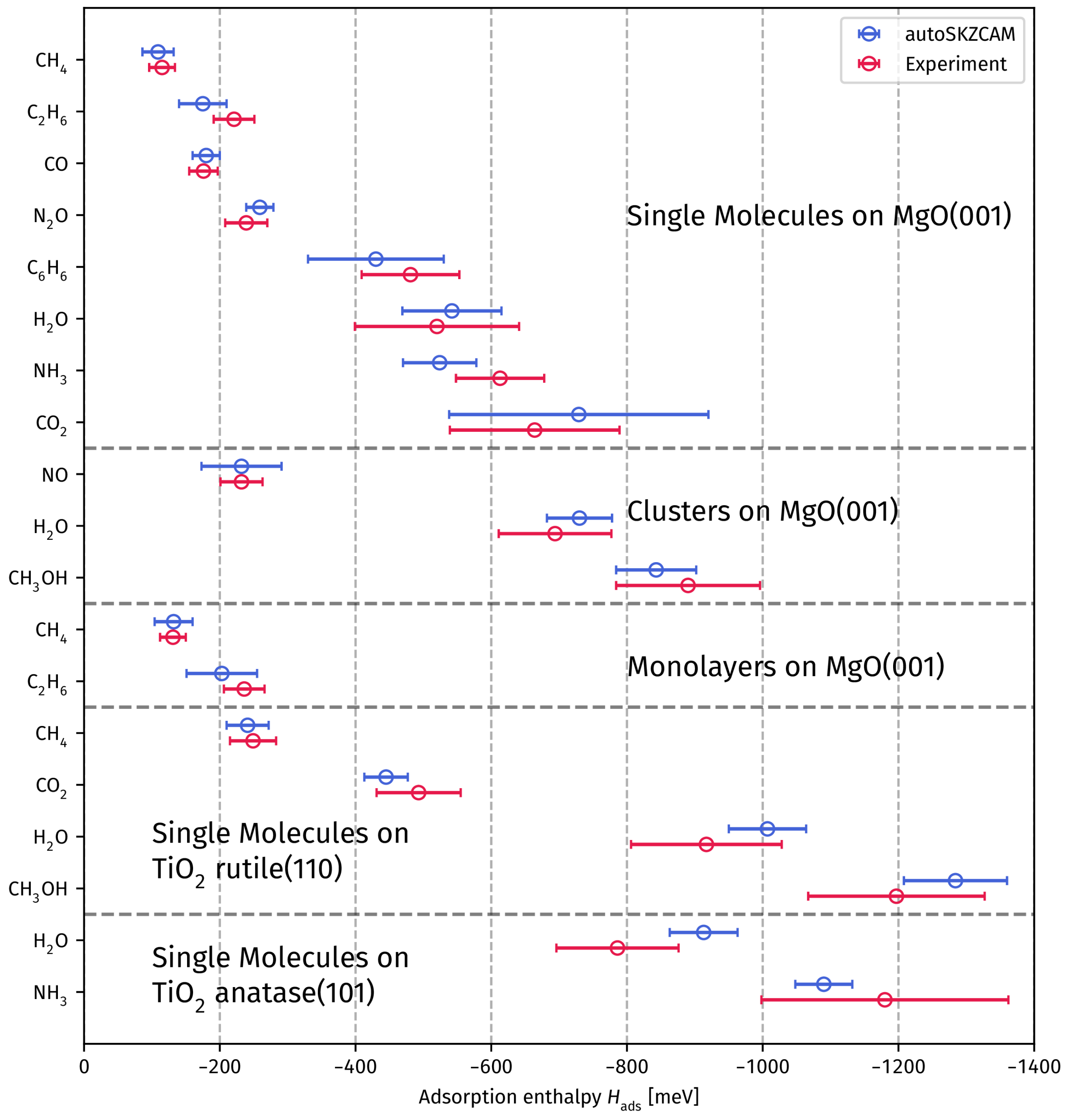

FIG. 17: A comparison of adsorption enthalpies computed with the autoSKZCAM framework against high-quality temperature programmed desorption experiments for a set of 19 adsorbate–surface combinations. These include single molecules adsorbed on the MgO(001) surface, monolayers adsorbed on MgO(001), single molecules adsorbed on TiO_2 rutile(110) and anatase(101) as well as clusters adsorbed on MgO(001). Experimental errors on H_{ads} are based on temperature programmed desorption analysis by Campbell and Sellers [3], taken as twice the standard deviation in the predicted pre-exponential factor against a test set of ∼20 adsorbed molecules (Section 11). Simulation errors are the root squared sum of several systematic contributions described in Section 9, with the majority arising from errors using a geometry optimised by density functional theory, which we estimate as twice the root-mean-squared-error from an ensemble of 6 density functional approximations.

TABLE 33: Comparison of the experimental and autoSKZCAM H_{ads} values (in meV) for the systems studied in this work. The Δ_{min} column shows the minimum difference between the experimental H_{ads} value and the autoSKZCAM H_{ads} value accounting for their error bars.

System	Expt. H_{ads}	Expt. ϵ	autoSKZCAM H_{ads}	autoSKZCAM ϵ	Δ_{min}
CH_4 on MgO(001)	-115	19	-109	23	0
C_2H_6 on MgO(001)	-221	30	-175	35	0
CO on MgO(001)	-176	21	-180	20	0
Parallel N_2O on MgO(001)	-239	31	-259	20	0
C_6H_6 on MgO(001)	-481	72	-430	100	0
Monomer H_2O on MgO(001)	-520	121	-542	73	0
NH_3 on MgO(001)	-613	65	-524	54	0
Chemisorbed CO_2 on MgO(001)	-664	125	-729	191	0
Monolayer CH_4 on MgO(001)	-131	19	-132	28	0
Monolayer C_2H_6 on MgO(001)	-236	30	-203	52	0
Dimer NO on MgO(001)	-232	31	-232	59	0
Dissociated Tetramer H_2O on MgO(001)	-694	83	-730	48	0
Dissociated Tetramer CH_3OH on MgO(001)	-890	106	-843	59	0
CH_4 on TiO_2 rutile(110)	-249	34	-241	31	0
Tilted CO_2 on TiO_2 rutile(110)	-493	62	-445	32	0
H_2O on TiO_2 rutile(110)	-917	111	-1007	57	0
CH_3OH on TiO_2 rutile(110)	-1197	130	-1284	76	0
H_2O on TiO_2 anatase(101)	-786	90	-913	50	0
NH_3 on TiO_2 anatase(101)	-1180	182	-1090	42	0

where the RMSD is increased from 58 meV to 102 meV when going from the system-specific ν approach suggested by Campbell and Sellers [3] to the standard approach of using $\nu = 10^{13}$.

TABLE 34: Comparison of the experimental H_{ads} values in meV with the autoSKZCAM H_{ads} values for the systems studied in this work. The H_{ads} values are compared with the re-analysed values in Table 32 and the values obtained by using the conventional $\log(\nu) = 13$ in temperature programmed desorption (TPD) experiments. The root mean squared deviation (RMSD) is also calculated against the autoSKZCAM H_{ads} values.

System	autoSKZCAM	Experiment (Table S1)	Experiment (ν=10^{13})
CH_4 on MgO(001)	-109 ± 23	-115	-114
C_2H_6 on MgO(001)	-175 ± 35	-221	-192
CO on MgO(001)	-180 ± 20	-176	-166
Parallel N_2O on MgO(001)	-259 ± 20	-239	-223
C_6H_6 on MgO(001)	-430 ± 100	-481	-413
Monomer H_2O on MgO(001)	-542 ± 73	-520	-520
NH_3 on MgO(001)	-524 ± 54	-613	-581
Chemisorbed CO_2 on MgO(001)	-729 ± 191	-664	-618
Monolayer CH_4 on MgO(001)	-132 ± 28	-131	-130
Monolayer C_2H_6 on MgO(001)	-203 ± 52	-236	-207
Dimer NO on MgO(001)	-232 ± 59	-232	-216
Dissociated Tetramer H_2O on MgO(001)	-730 ± 48	-694	-624
Dissociated Tetramer CH_3OH on MgO(001)	-843 ± 59	-890	-759
CH_4 on TiO_2 rutile(110)	-241 ± 31	-249	-216
Tilted CO_2 on TiO_2 rutile(110)	-445 ± 32	-493	-471
H_2O on TiO_2 rutile(110)	-1007 ± 57	-917	-814
CH_3OH on TiO_2 rutile(110)	-1284 ± 76	-1197	-1013
H_2O on TiO_2 anatase(101)	-913 ± 50	-786	-704
NH_3 on TiO_2 anatase(101)	-1090 ± 42	-1180	-1155
RMSD		58	102

13. PREVIOUS COMPUTATIONAL LITERATURE

Many of the adsorbate–surface systems within this work have been studied before by both DFT and cWFT. In particular, owing to its affordable nature and popularity, DFT has been widely used, providing several predictions for each system and we have collated some of this previous literature in Table 35. Much of the early work in the late 1990's and early 2000's have utilised embedded cluster calculations (often with hybrid functionals such as B3LYP), with the current DFT workhorse being planewave periodic codes. For each system, we observe large deviations across the DFT literature, varying by over 300 meV for most systems. These variations mostly arise from differences in the exchange-correlation functional but even for the same functional, predictions can significantly vary due to differences in the basis set and pseudopotential treatment, which are discussed within each cited literature.

Importantly, for some of these systems, even the predicted adsorption configuration of the adsorbate is not known and different studies may suggest different geometries. For example, whether CO_2 adopts a chemisorbed (bent) or physisorbed (linear) geometry on the MgO(001) has been under debate, with studies which point towards either. Similarly, a myriad of geometries have been predicted for NO on MgO(001) by different studies, many of which cite agreement to experiment. With DFT, this agreement can be fortuitous many times due to errors in the surface model, DFA, electronic structures or neglect of thermal contributions. These discrepancies have highlighted the need for accurate approaches such as the autoSKZCAM framework that can predict the correct geometries (with H_{ads} that match experiments) that get the right answers for the right reasons; we highlight its success in predicting the ground-state configuration for several systems in Section 1.

While less common, calculations with cWFT have also been applied before to a selection of the studied systems and we highlight these previous work (and the method used) in Table 35. As we showed in Fig. 2 of the main text, the deviations between methods from cWFT can be significant (to as large as 500 meV for some systems). These deviations can arise from errors in the method used and also from electronic structure parameters; methods from cWFT are severely affected by basis set and surface model, as has been discussed extensively for CO on MgO(001) in Ref. 85. Overall, these deviations highlight the need for going to a high level of theory, namely CCSD(T) and accurate surface models — the targets of the autoSKZCAM framework.

TABLE 35: Compilation of E_{ads} values (in meV) of previous density functional theory (DFT) and correlated wave-function theory (cWFT) literature for the systems studied within this work.

System	DFT	WFT
CH_4 on MgO(001)	-50 [148], 2 [45], 17 [149], -300 [20], -120 [23], -152 [122]	-134 [MP2+ΔCC] [150]
C_2H_6 on MgO(001)	-127 [150], -154 [150]	-196 [MP2+ΔCC] [150]
CO on MgO(001)	-9 to -282 [151], -175 to -408 [152]	-207 [LNO-CCSD(T)] [153], -199 [LNO-CCSD(T)] [85], -230 [MP2+ΔCC] [53], 70 [CCSD(T)] [23], -398 [CCSD] [154], -72 [RPA@PBE] [59], -310 [RPA@PBE+rSE] [59]
N_2O on MgO(001)	-258 [41], >0 [40], -137 [41]	
C_6H_6 on MgO(001)	-20 [148]	
Monomer H_2O on MgO(001)	-342 [155], -500 [156], -340 [157], -422 to -667 [158]	-574 [ΔCC] [53], -480 [DMC] [159], -608 [RPA@PBE+rSE] [59], -492 [RPA@PBE] [59]
NH_3 on MgO(001)	-668 [160], -867 [161]	-608 [PCT] [162]
CO_2 on MgO(001)	Chemisorbed: -1000 [20], 135 [16], -680 [22], -640 [23], -380 [21], Physisorbed: -320 [163], -90 [19], -126 [18], -120 [20]	Chemisorbed: 870 [MP2] [15], -492 [RPA@PBE] [This work], -494 [RPA@PBE+rSE] [This work], Physisorbed: -340 [MP2] [17]
NO on MgO(001)	Hollow: -312 [164], Bent-Mg: -520 [165], Upright-Mg: -260 [166], -297 [167], Bent-O: -464 [168], -312 [169, 170], Dimer: -30 [171], -40 [31], Bent-Bridge: -150 [172]	Bent-Mg: 29 [RPA@PBE] [59], -360 [RPA@PBE+rSE] [59]
Cluster CH_3OH on MgO(001)	-718 [173], -466 [174], -640 [175], -964 [7], -508 [6]	-598 [MP2] [8]
Cluster H_2O on MgO(001)	-610 [176], -592 [177], -720 [178]	
Monolayer CH_4 on MgO(001)	-18 [46, 179], -124 to -353 [152]	-79 [PCI-80] [45], -114 [LMP2] [180], -138 [MP2+ΔCC] [49], -145 [MP2+ΔCC] [53], -87 [RPA@PBE] [59], -140 [RPA@PBE+rSE] [59]
Monolayer C_2H_6 on MgO(001)	-234 to -568 [152]	-242 [MP2+ΔCC] [53]
CH_4 on TiO_2 rutile(110)	-48 to -412 [33], -360 [181]	-408 [CCSD(T)] [33]
CO_2 on TiO_2 rutile(110)	-95 to -559 [33], -640 [182]	-542 [CCSD(T)] [33]
H_2O on TiO_2 rutile(110)	-442 to -950 [33], -840 [183], -1638 [184], -1032 [39]	-1492 [MP2] [185], -984 [DLPNO-CCSD(T)] [33], -964 [LNO-CCSD(T)] [186], -1390 [CCSD] [187]
CH_3OH on TiO_2 rutile(110)	-468 to -1145 [33], -1490 [188], -1234 [189], -760 [190]	-1106 [DLPNO-CCSD(T)] [33]
H_2O on TiO_2 anatase(101)	-740 [191], -650 [192], -1088 [193], -977 [194]	-1170 [DLPNO-CCSD(T)] [195]
NH_3 on TiO_2 anatase(101)	-1200 [147], -1113 [193], -1153 [196], -1193 [197]	

14. BENCHMARKING THE COST OF THE AUTOSKZCAM FRAMEWORK

We perform an analysis of the cost for computing the interaction energy E_{int} from the autoSKZCAM framework against a GGA-based (PBE-D3) and hybrid-based (PBE0-TS/HI) DFA. In both cases, we have attempted to make the calculations efficient to the best of our abilities. For example, for periodic hybrid calculations, we make use of the Adaptively Compressed Exchange Operator [198] and use an initial wave-function coming from the GGA calculation to enable efficient self-consistent field (SCF) energy convergence to the cutoff of 10^{-5} eV (looser than our standard settings in Section 8 A). We also use a smaller energy cutoff of 520 eV for both the GGA and hybrid DFT calculations. The cost for the autoSKZCAM framework is the sum of all the individual contributions to E_{int} outlined in Table 7, with the same corresponding computational details described in Section 5. The efficiency of the autoSKZCAM framework calculations for CO_2 on MgO(001) is further enhanced by performing both MP2 and CCSD(T) calculations using the aVXZ basis sets rather than awCVXZ and only treating the valence electrons in the correlation treatment. A further Δ_{core} contribution is calculated by performing additional CBS(awCVTZ/awCVQZ) calculations for the first three clusters generated by the SKZCAM protocol, commensurate with the procedure used for H_2O on TiO_2 rutile(110).

The costs in CPU-hours (CPUh) are compared in Table 36 for CO_2 on MgO(001) and H_2O on TiO_2 rutile(110). Both periodic DFT and autoSKZCAM costs were assessed on 2.1 GHz, 18-core Intel Xeon E5-2695 (Broadwell) series processors on the Cirrus high-performance computing (HPC) cluster [https://www.cirrus.ac.uk/], barring hybrid DFT calculations for H_2O on TiO_2 rutile(110), which were evaluated on improved hardware, involving 3.1 (turbo-boosted to 3.9) GHz Intel Xeon Platinum 8174 (Skylake) processors on the Vienna Scientific Cluster (VSC-4) [https://vsc.ac.at//systems/vsc-4/]. In addition, for the hybrid DFT calculations involving H_2O on TiO_2 rutile(110), we have provided costs to perform $1 \times 1 \times 1$ and $2 \times 2 \times 1$ k-point grids are provided, as the former can achieve significant speed-ups while trading some accuracy ($\sim$20 meV).

We find that the cost with the autoSKZCAM framework for CO_2 on MgO(001) is slightly higher (less than 2 times) than hybrid DFT, which is in turn an order of magnitude more expensive than the GGA calculation. However, when moving towards the more complex H_2O on TiO_2 rutile(110) – involving heavier atoms and a larger number of atoms in the periodic model of its surface – the cost of the autoSKZCAM framework becomes comparable to periodic hybrid DFT calculation (with a $2 \times 2 \times 1$ k-point grid). The GGA calculation remains an order of magnitude cheaper than the autoSKZCAM framework for this system. It should be highlighted that the cost

TABLE 36: Computational cost in CPU hours for periodic DFT, both a GGA (PBE-D3) and hybrid (PBE0-TS/HI), compared to autoSKZCAM for the CO_2 on MgO(001) and H_2O on TiO_2 rutile(110) adsorbate–surface systems. Details of these calculations are described in the text.

	GGA	hybrid	autoSKZCAM
CO_2 on MgO(001)	200	1500	2900
H_2O on TiO_2 rutile(110)	300	700 to 10300	6700

moving from CO_2 on MgO(001) to H_2O on TiO_2 rutile(110) does not change significantly (less than 3 times increase) for the autoSKZCAM framework despite its increased complexity. This feature of the autoSKZCAM framework arises because the size of the clusters selected by the SKZCAM protocol (described in Section 6 A) does not depend on the complexity of the surface, and should remain similar in size between different types of surfaces since it is generated based on radial cutoffs.

It is also useful to highlight the cost of the autoSKZCAM framework in relation to previous high-level calculations. In particular, CO on MgO(001) has been the prototypical surface system for cWFT methods, as highlighted in Ref. 85. We gather previous estimates in Table 37; these should be taken as rough estimates as they were all performed on different computing systems. Out of all of these previous works, autoSKZCAM is by far the cheapest, with a cost of $\sim$600 CPUh. This is improved over the previous SKZCAM protocol calculations due to the described improvements in Section 6 G. Ye and Berkelbach previously performed periodic LNO-CCSD(T) calculations for this system and arrived at a cost of $\sim$18,000 CPUh. Compared to this, periodic CCSD(T) (without the LNO approximation) was shown to take $\sim$200,000 CPUh to perform in Ref. 85, with periodic DMC being even more costly at $\sim$1,000,000 CPUh. In the present study, the RPA calculations took $\sim$4,000 CPUh, relatively comparable to hybrid DFT at $\sim$1,000 CPUh.

TABLE 37: Rough computational cost in CPU hours for several methods from correlated wave-function theory applied to CO on MgO(001). Details are provided in their respective references. As a guide, hybrid DFT costs $\sim$1,000 CPUh [85] for this system.

Method	Cost [CPUh]
autoSKZCAM framework (This work)	$\sim$600
RPA (This work)	$\sim$4000
Periodic LNO-CCSD(T) [153]	$\sim$18000
Cluster CCSD(T) [85] [SKZCAM protocol]	$\sim$20000
Periodic CCSD(T) [85]	$\sim$200000
Periodic DMC [85]	$\sim$1000000

[1] B. X. Shi, Research data supporting: An accurate and efficient framework for predictive insights into ionic surface chemistry, https://github.com/benshi97/Data_autoSKZCAM (2024).

[2] J. Günster, G. Liu, J. Stultz, and D. W. Goodman, Interaction of methanol and water on MgO(100) studied by ultraviolet photoelectron and metastable impact electron spectroscopies, J. Chem. Phys. **110**, 2558 (1999).

[3] C. T. Campbell and J. R. V. Sellers, Enthalpies and entropies of adsorption on well-defined oxide surfaces: Experiment measurements, Chem. Rev. **113**, 4106 (2013).

[4] Y. Zhang and W. Yang, Comment on "Generalized gradient approximation made simple", Phys. Rev. Lett. **80**, 890 (1998).

[5] E. Caldeweyher, S. Ehlert, A. Hansen, H. Neugebauer, S. Spicher, C. Bannwarth, and S. Grimme, A generally applicable atomic-charge dependent London dispersion correction, J. Chem. Phys. **150**, 154122 (2019).

[6] H. Petitjean, K. Tarasov, F. Delbecq, P. Sautet, J. M. Krafft, P. Bazin, M. C. Paganini, E. Giamello, M. Che, H. Lauron-Pernot, and G. Costentin, Quantitative investigation of MgO Brønsted basicity: DFT, IR, and calorimetry study of methanol adsorption, J. Phys. Chem. C **114**, 3008 (2010).

[7] M. A. Sainna, S. Nanavati, C. Black, L. Smith, K. Mugford, H. Jenkins, M. Douthwaite, N. F. Dummer, C. R. A. Catlow, G. J. Hutchings, S. H. Taylor, A. J. Logsdail, and D. J. Willock, A combined periodic DFT and QM/MM approach to understand the radical mechanism of the catalytic production of methanol from glycerol, Faraday Discuss. **229**, 108 (2021).

[8] M. M. Branda, J. E. Peralta, N. J. Castellani, and R. H. Contreras, Theoretical study of charge transfer interactions in methanol adsorbed on magnesium oxide, Surf. Sci. **504**, 235 (2002).

[9] M. Trabelsi, S. Saidi, C. Chefi, C. Martin, S. Lucas, D. Ferry, and J. Suzanne, Thermodynamic and structural study of methanol thin films adsorbed on MgO(100), Surf. Sci. **566–568**, 789 (2004).

[10] J. Rudberg and M. Foster, Adsorption of methanol on the MgO(100) surface: An infrared study at room temperature, J. Phys. Chem. B **108**, 18311 (2004).

[11] D. Ferry, A. Glebov, V. Senz, J. Suzanne, J. P. Toennies, and H. Weiss, The properties of a two-dimensional water layer on MgO(001), Surf. Sci. **377–379**, 634 (1997).

[12] D. Ferry, S. Picaud, P. N. M. Hoang, C. Girardet, L. Giordano, B. Demirdjian, and J. Suzanne,

Water monolayers on MgO(100): Structural investigations by LEED experiments, tensor LEED dynamical analysis and potential calculations, Surf. Sci. **409**, 101 (1998).

[13] D. L. Meixner, D. A. Arthur, and S. M. George, Kinetics of desorption, adsorption, and surface diffusion of CO_2 on MgO(100), Surf. Sci. **261**, 141 (1992).

[14] A. Chakradhar and U. Burghaus, Carbon dioxide adsorption on MgO(001)–CO_2 kinetics and dynamics, Surf. Sci. **616**, 171 (2013).

[15] G. Pacchioni, J. M. Ricart, and F. Illas, Ab initio cluster model calculations on the chemisorption of CO_2 and SO_2 probe molecules on MgO and CaO (100) surfaces. A theoretical measure of oxide basicity, J. Am. Chem. Soc. **116**, 10152 (1994).

[16] M. B. Jensen, L. G. M. Pettersson, O. Swang, and U. Olsbye, CO_2 sorption on MgO and CaO Surfaces: A comparative quantum chemical cluster study, J. Phys. Chem. B **109**, 16774 (2005).

[17] G. Pacchioni, Physisorbed and chemisorbed CO_2 at surface and step sites of the MgO(100) surface, Surf. Sci. **281**, 207 (1993).

[18] R. Hammami, A. Dhouib, S. Fernandez, and C. Minot, CO_2 adsorption on (001) surfaces of metal monoxides with rock-salt structure, Catal. Today **139**, 227 (2008).

[19] D. Cornu, H. Guesmi, J.-M. Krafft, and H. Lauron-Pernot, Lewis acido-basic interactions between CO_2 and MgO surface: DFT and DRIFT approaches, J. Phys. Chem. C **116**, 6645 (2012).

[20] M. A. Manae, L. Dheer, S. Rai, S. Shetty, and U. V. Waghmare, Activation of CO_2 and CH_4 on MgO surfaces: Mechanistic insights from first-principles theory, Phys. Chem. Chem. Phys. **24**, 1415 (2022).

[21] J. Baltrusaitis, C. Hatch, and R. Orlando, Periodic DFT study of acidic trace atmospheric gas molecule adsorption on Ca- and Fe-doped MgO(001) surface basic sites, J. Phys. Chem. A **116**, 7950 (2012).

[22] C. A. Downing, A. A. Sokol, and C. R. A. Catlow, The reactivity of CO_2 on the MgO(100) surface, Phys. Chem. Chem. Phys. **16**, 184 (2013).

[23] A. Mazheika and S. V. Levchenko, Ni substitutional defects in bulk and at the (001) surface of MgO from first-principles calculations, J. Phys. Chem. C **120**, 26934 (2016).

[24] Y. Yanagisawa, K. Takaoka, S. Yamabe, and T. Ito, Interaction of CO_2 with magnesium oxide surfaces: A TPD, FTIR, and cluster-model calculation study, J. Phys. Chem. **99**, 3704 (1995).

[25] T. Ito, H. Kobayashi, and T. Tashiro, Roles of low-coordinated surface ions in adsorption of gases on MgO, Nouv. Cim. D **19**, 1695 (1997).

[26] N. McQueen, P. Kelemen, G. Dipple, P. Renforth, and J. Wilcox, Ambient weathering of magne-

sium oxide for CO2 removal from air, Nat. Commun. **11**, 3299 (2020).

[27] F. Donat and C. R. Müller, Prospects of MgO-based sorbents for CO_2 capture applications at high temperatures, Curr. Opin. Green Sustainable Chem. **36**, 100645 (2022).

[28] Y. Fu, L. Zhang, B. Yue, X. Chen, and H. He, Simultaneous characterization of solid acidity and basicity of metal oxide catalysts via the solid-state nmr technique, J. Phys. Chem. C **122**, 24094 (2018).

[29] J.-H. Du, L. Chen, B. Zhang, K. Chen, M. Wang, Y. Wang, I. Hung, Z. Gan, X.-P. Wu, X.-Q. Gong, and L. Peng, Identification of CO_2 adsorption sites on MgO nanosheets by solid-state nuclear magnetic resonance spectroscopy, Nat. Commun. **13**, 707 (2022).

[30] R. Wichtendahl, M. Rodriguez-Rodrigo, U. Härtel, H. Kuhlenbeck, and H.-J. Freund, Thermodesorption of CO and NO from vacuum-cleaved NiO(100) and MgO(100), Phys. Status Solidi A **173**, 93 (1999).

[31] C. Di Valentin, G. Pacchioni, M. Chiesa, E. Giamello, S. Abbet, and U. Heiz, NO monomers on MgO powders and thin films, J. Phys. Chem. B **106**, 1637 (2002).

[32] E. E. Platero, G. Spoto, and A. Zecchina, Spectroscopic study of NO adsorption on magnesium oxide, nickel oxide and their solid solutions, J. Chem. Soc., Faraday Trans. 1 **81**, 1283 (1985).

[33] A. Kubas, D. Berger, H. Oberhofer, D. Maganas, K. Reuter, and F. Neese, Surface adsorption energetics studied with "gold standard" wave-function-based ab initio methods: Small-molecule binding to TiO_2(110), J. Phys. Chem. Lett. **7**, 4207 (2016).

[34] D. C. Sorescu, J. Lee, W. A. Al-Saidi, and K. D. Jordan, CO_2 adsorption on TiO_2(110) rutile: Insight from dispersion-corrected density functional theory calculations and scanning tunneling microscopy experiments, J. Chem. Phys. **134**, 104707 (2011).

[35] J. Lee, D. C. Sorescu, X. Deng, and K. D. Jordan, Diffusion of CO_2 on the rutile TiO_2(110) surface, J. Phys. Chem. Lett. **2**, 3114 (2011).

[36] X. Lin, Y. Yoon, N. G. Petrik, Z. Li, Z.-T. Wang, V.-A. Glezakou, B. D. Kay, I. Lyubinetsky, G. A. Kimmel, R. Rousseau, and Z. Dohnálek, Structure and dynamics of CO_2 on rutile TiO_2(110)-1$\times$1, J. Phys. Chem. C **116**, 26322 (2012).

[37] Y. Cao, S. Hu, M. Yu, S. Yan, and M. Xu, Adsorption and interaction of CO_2 on rutile TiO_2(110) surfaces: A combined UHV-FTIRS and theoretical simulation study, Phys. Chem. Chem. Phys. **17**, 23994 (2015).

[38] R. C. E. Hamlyn, M. Mahapatra, D. C. Grinter, F. Xu, S. Luo, R. M. Palomino, S. Kattel, I. Waluyo, P. Liu, D. J. Stacchiola, S. D. Senanayake, and J. A. Rodriguez, Imaging the ordering of

a weakly adsorbed two-dimensional condensate: Ambient-pressure microscopy and spectroscopy of CO_2 molecules on rutile TiO_2 (110), Phys. Chem. Chem. Phys. **20**, 13122 (2018).

[39] D. C. Sorescu, J. Lee, W. A. Al-Saidi, and K. D. Jordan, Coadsorption properties of CO_2 and H_2O on TiO_2 rutile (110): A dispersion-corrected DFT study, J. Chem. Phys. **137**, 074704 (2012).

[40] A. Scagnelli, C. D. Valentin, and G. Pacchioni, Catalytic dissociation of N_2O on pure and Ni-doped MgO surfaces, Surf. Sci. **600**, 386 (2006).

[41] Z. Huesges, C. Müller, B. Paulus, and L. Maschio, Dispersion corrected DFT calculations for the adsorption of N_2O on MgO, Surf. Sci. **627**, 11 (2014).

[42] M. Causa, E. Kotomin, C. Pisani, and C. Roetti, The MgO(110) surface and CO adsorption thereon. II. CO adsorption, J. Phys. C: Solid State Phys. **20**, 4991 (1987).

[43] G. Pacchioni, G. Cogliandro, and P. S. Bagus, Molecular orbital cluster model study of bonding and vibrations of CO adsorbed on MgO surface, Int. J. Quantum Chem. **42**, 1115 (1992).

[44] A. Alavi, Molecular-dynamics simulation of methane adsorbed on MgO: Evidence for a Kosterlitz-Thouless transition, Mol. Phys. **71**, 1173 (1990).

[45] K. Todnem, K. J. Børve, and M. Nygren, Molecular adsorption of methane and methyl onto MgO(100) An embedded-cluster study, Surf. Sci. **421**, 296 (1999).

[46] M. L. Drummond, B. G. Sumpter, W. A. Shelton, and J. Z. Larese, Density functional investigation of the adsorption of a methane monolayer on an MgO(100) surface, Phys. Rev. B **73**, 195313 (2006).

[47] J. Z. Larese, J. M. Hastings, L. Passell, D. Smith, and D. Richter, Rotational tunneling of methane on MgO surfaces: A neutron scattering study, J. Chem. Phys. **95**, 6997 (1991).

[48] J. Z. Larese, D. M. y Marero, D. S. Sivia, and C. J. Carlile, Tracking the evolution of interatomic potentials with high resolution inelastic neutron spectroscopy, Phys. Rev. Lett. **87**, 206102 (2001).

[49] S. Tosoni and J. Sauer, Accurate quantum chemical energies for the interaction of hydrocarbons with oxide surfaces: CH_4/MgO(001), Phys. Chem. Chem. Phys. **12**, 14330 (2010).

[50] M. Sidoumou, T. Angot, and J. Suzanne, Ethane adsorbed on MgO(100) single crystal surfaces: A high resolution LEED study, Surf. Sci. **272**, 347 (1992).

[51] P. N. M. Hoang, C. Girardet, M. Sidoumou, and J. Suzanne, Structure of ethane monolayers adsorbed on MgO(100): Experiments and calculations, Phys. Rev. B **48**, 12183 (1993).

[52] M. Trabelsi, S. Saidi, and J. P. Coulomb, Structural study of C_2D_6 thin films adsorbed on MgO(100), Indian J. Pure Appl. Phys. **53**, 748 (2015).

[53] M. Alessio, F. A. Bischoff, and J. Sauer, Chemically accurate adsorption energies for methane

and ethane monolayers on the MgO(001) surface, Phys. Chem. Chem. Phys. **20**, 9760 (2018).

[54] T. Bligaard, R. M. Bullock, C. T. Campbell, J. G. Chen, B. C. Gates, R. J. Gorte, C. W. Jones, W. D. Jones, J. R. Kitchin, and S. L. Scott, Toward benchmarking in catalysis science: Best practices, challenges, and opportunities, ACS Catal. **6**, 2590 (2016).

[55] J. Řezáč, K. E. Riley, and P. Hobza, S66: A well-balanced database of benchmark interaction energies relevant to biomolecular structures, J. Chem. Theory Comput. **7**, 2427 (2011).

[56] L. Goerigk, A. Hansen, C. Bauer, S. Ehrlich, A. Najibi, and S. Grimme, A look at the density functional theory zoo with the advanced GMTKN55 database for general main group thermochemistry, kinetics and noncovalent interactions, Phys. Chem. Chem. Phys. **19**, 32184 (2017).

[57] J. Klimeš, M. Kaltak, E. Maggio, and G. Kresse, Singles correlation energy contributions in solids, J. Chem. Phys. **143**, 102816 (2015).

[58] M. Kaltak, J. Klimeš, and G. Kresse, Cubic scaling algorithm for the random phase approximation: Self-interstitials and vacancies in Si, Phys. Rev. B **90**, 054115 (2014).

[59] M. Bajdich, J. K. Nørskov, and A. Vojvodic, Surface energetics of alkaline-earth metal oxides: Trends in stability and adsorption of small molecules, Phys. Rev. B **91**, 155401 (2015).

[60] P. Sabatier, Hydrogénations et déshydrogénations par catalyse, Ber. Dtsch. Chem. Ges. **44**, 1984 (1911).

[61] J. Sauer, Ab initio calculations for molecule–surface interactions with chemical accuracy, Acc. Chem. Res. **52**, 3502 (2019).

[62] F. Berger, M. Rybicki, and J. Sauer, Molecular dynamics with chemical accuracy – Alkane adsorption in acidic zeolites, ACS Catal. **13**, 2011 (2023).

[63] C. Xia, T. Hong Chuong Nguyen, X. Cuong Nguyen, S. Young Kim, D. L. T. Nguyen, P. Raizada, P. Singh, V.-H. Nguyen, C. Chien Nguyen, V. Chinh Hoang, and Q. Van Le, Emerging cocatalysts in TiO_2-based photocatalysts for light-driven catalytic hydrogen evolution: Progress and perspectives, Fuel **307**, 121745 (2022).

[64] Q. Wang and K. Domen, Particulate photocatalysts for light-driven water splitting: Mechanisms, challenges, and design strategies, Chem. Rev. **120**, 919 (2020).

[65] S. Wang, R. Xiang, P. Liao, J. Kang, S. Li, M. Mao, L. Liu, and G. Li, Highly efficient one-pot electrosynthesis of oxime ethers from NO_x over ultrafine MgO nanoparticles derived from Mg-based metal-organic frameworks, Angew. Chem., Int. Ed. **63**, e202405553 (2024).

[66] Chr. Møller and M. S. Plesset, Note on an approximation treatment for many-electron systems, Phys. Rev. **46**, 618 (1934).

[67] K. Raghavachari, G. W. Trucks, J. A. Pople, and M. Head-Gordon, A fifth-order perturbation comparison of electron correlation theories, Chem. Phys. Lett. **157**, 479 (1989).

[68] F. Neese, F. Wennmohs, U. Becker, and C. Riplinger, The ORCA quantum chemistry program package, J. Chem. Phys. **152**, 224108 (2020).

[69] M. Kállay, P. R. Nagy, D. Mester, Z. Rolik, G. Samu, J. Csontos, J. Csóka, P. B. Szabó, L. Gyevi-Nagy, B. Hégely, I. Ladjánszki, L. Szegedy, B. Ladóczki, K. Petrov, M. Farkas, P. D. Mezei, and Á. Ganyecz, The MRCC program system: Accurate quantum chemistry from water to proteins, J. Chem. Phys. **152**, 074107 (2020).

[70] P. R. Nagy, G. Samu, and M. Kállay, Optimization of the linear-scaling local natural orbital CCSD(T) method: Improved algorithm and benchmark applications, J. Chem. Theory Comput. **14**, 4193 (2018).

[71] P. R. Nagy and M. Kállay, Approaching the basis set limit of CCSD(T) energies for large molecules with local natural orbital coupled-cluster methods, J. Chem. Theory Comput. **15**, 5275 (2019).

[72] C. Riplinger and F. Neese, An efficient and near linear scaling pair natural orbital based local coupled cluster method, J. Chem. Phys. **138**, 034106 (2013).

[73] C. Riplinger, B. Sandhoefer, A. Hansen, and F. Neese, Natural triple excitations in local coupled cluster calculations with pair natural orbitals, J. Chem. Phys. **139**, 134101 (2013).

[74] C. Riplinger and F. Neese, An efficient and near linear scaling pair natural orbital based local coupled cluster method, J. Chem. Phys. **138**, 034106 (2013).

[75] C. Riplinger, P. Pinski, U. Becker, E. F. Valeev, and F. Neese, Sparse maps—A systematic infrastructure for reduced-scaling electronic structure methods. II. Linear scaling domain based pair natural orbital coupled cluster theory, J. Chem. Phys. **144**, 024109 (2016).

[76] K. A. Peterson and T. H. Dunning, Accurate correlation consistent basis sets for molecular core–valence correlation effects: The second row atoms Al–Ar, and the first row atoms B–Ne revisited, J. Chem. Phys. **117**, 10548 (2002).

[77] N. B. Balabanov and K. A. Peterson, Systematically convergent basis sets for transition metals. I. All-electron correlation consistent basis sets for the 3d elements Sc–Zn, J. Chem. Phys. **123**, 064107 (2005).

[78] G. Bistoni, C. Riplinger, Y. Minenkov, L. Cavallo, A. A. Auer, and F. Neese, Treating subvalence correlation effects in domain based pair natural orbital coupled cluster calculations: An out-of-the-box approach, J. Chem. Theory Comput. **13**, 3220 (2017).

[79] F. Neese and E. F. Valeev, Revisiting the atomic natural orbital approach for basis sets: Robust

systematic basis sets for explicitly correlated and conventional correlated ab initio methods, J. Chem. Theory Comput. **7**, 33 (2011).

[80] F. Weigend, M. Häser, H. Patzelt, and R. Ahlrichs, RI-MP2: Optimized auxiliary basis sets and demonstration of efficiency, Chem. Phys. Lett. **294**, 143 (1998).

[81] A. Hellweg, C. Hättig, S. Höfener, and W. Klopper, Optimized accurate auxiliary basis sets for RI-MP2 and RI-CC2 calculations for the atoms Rb to Rn, Theor. Chem. Acc. **117**, 587 (2007).

[82] G. L. Stoychev, A. A. Auer, and F. Neese, Automatic generation of auxiliary basis sets, J. Chem. Theory Comput. **13**, 554 (2017).

[83] S. Lehtola, Straightforward and accurate automatic auxiliary basis set generation for molecular calculations with atomic orbital basis sets, J. Chem. Theory Comput. **17**, 6886 (2021).

[84] B. X. Shi, V. Kapil, A. Zen, J. Chen, A. Alavi, and A. Michaelides, General embedded cluster protocol for accurate modeling of oxygen vacancies in metal-oxides, J. Chem. Phys. **156**, 124704 (2022).

[85] B. X. Shi, A. Zen, V. Kapil, P. R. Nagy, A. Grüneis, and A. Michaelides, Many-body methods for surface chemistry come of age: Achieving consensus with experiments, J. Am. Chem. Soc. **145**, 25372 (2023).

[86] G. Pacchioni, P. S. Bagus, and F. Parmigiani, Cluster models for surface and bulk phenomena (Springer Science & Business Media, 2013).

[87] Y. Lu, K. Sen, C. Yong, D. S. D. Gunn, J. A. Purton, J. Guan, A. Desmoutier, J. A. Nasir, X. Zhang, L. Zhu, Q. Hou, J. Jackson-Masters, S. Watts, R. Hanson, H. N. Thomas, O. Jayawardena, A. J. Logsdail, S. M. Woodley, H. M. Senn, P. Sherwood, C. R. A. Catlow, A. A. Sokol, and T. W. Keal, Multiscale QM/MM modelling of catalytic systems with ChemShell, Phys. Chem. Chem. Phys. **25**, 21816 (2023).

[88] N. D. Drummond, R. J. Needs, A. Sorouri, and W. M. C. Foulkes, Finite-size errors in continuum quantum Monte Carlo calculations, Phys. Rev. B **78**, 125106 (2008).

[89] Y. S. Al-Hamdani, M. Rossi, D. Alfè, T. Tsatsoulis, B. Ramberger, J. G. Brandenburg, A. Zen, G. Kresse, A. Grüneis, A. Tkatchenko, and A. Michaelides, Properties of the water to boron nitride interaction: From zero to two dimensions with benchmark accuracy, J. Chem. Phys. **147**, 044710 (2017).

[90] T. Bredow, L. Giordano, F. Cinquini, and G. Pacchioni, Electronic properties of rutile TiO_2 ultrathin films: Odd-even oscillations with the number of layers, Phys. Rev. B **70**, 035419 (2004).

[91] M. Svensson, S. Humbel, R. D. J. Froese, T. Matsubara, S. Sieber, and K. Morokuma, ONIOM:

A multilayered integrated MO + MM method for geometry optimizations and single point energy predictions. A test for Diels-Alder reactions and $Pt(P(t\text{-}Bu)_3)_2 + H_2$ oxidative addition, J. Phys. Chem. **100**, 19357 (1996).

[92] J. A. Pople, Quantum chemical models (Nobel Lecture), Angew. Chem. Int. Ed. **38**, 1894 (1999).

[93] T. Tsatsoulis, F. Hummel, D. Usvyat, M. Schütz, G. H. Booth, S. S. Binnie, M. J. Gillan, D. Alfè, A. Michaelides, and A. Grüneis, A comparison between quantum chemistry and quantum Monte Carlo techniques for the adsorption of water on the (001) LiH surface, J. Chem. Phys. **146**, 204108 (2017).

[94] A. G. Cabello-Cartagena, J. Vogt, and H. Weiss, Structure and infrared absorption of the first layer C_2H_2 on the NaCl(100) single-crystal surface, J. Chem. Phys. **132**, 074706 (2010).

[95] S. K. Dunn and G. E. Ewing, Infrared spectra and structure of acetylene on sodium chloride (100), J. Phys. Chem. **96**, 5284 (1992).

[96] A. Allouche, Quantum ab initio study of acetylene adsorption on NaCl(100) I. Topology and adsorption energy, Surf. Sci. **374**, 117 (1997).

[97] S. Picaud, P. N. M. Hoang, C. Girardet, A. Glebov, R. E. Miller, and J. P. Toennies, Phonon-libron dynamics of acetylene adsorbed on NaCl(001), Phys. Rev. B **57**, 10090 (1998).

[98] B. X. Shi, D. J. Wales, A. Michaelides, and C. W. Myung, Going for gold(-standard): Attaining coupled cluster accuracy in oxide-supported nanoclusters, J. Chem. Theory Comput. **20**, 5306 (2024).

[99] K. R. Bryenton, A. A. Adeleke, S. G. Dale, and E. R. Johnson, Delocalization error: The greatest outstanding challenge in density-functional theory, WIREs Comput. Mol. Sci. **13**, e1631 (2023).

[100] A. Dkhissi, P. Soulard, A. Perrin, and N. Lacome, The NO dimer, J. Mol. Spectrosc. **183**, 12 (1997).

[101] E. A. Wade, J. I. Cline, K. T. Lorenz, C. Hayden, and D. W. Chandler, Direct measurement of the binding energy of the NO dimer, J. Chem. Phys. **116**, 4755 (2002).

[102] R. González-Luque, M. Merchán, and B. O. Roos, A theoretical determination of the dissociation energy of the nitric oxide dimer, Theoret. Chim. Acta **88**, 425 (1994).

[103] R. Sayós, R. Valero, J. M. Anglada, and M. González, Theoretical investigation of the eight low-lying electronic states of the cis- and trans-nitric oxide dimers and its isomerization using multiconfigurational second-order perturbation theory (CASPT2), J. Chem. Phys. **112**, 6608 (2000).

[104] J. Ivanic, M. W. Schmidt, and B. Luke, High-level theoretical study of the NO dimer and tetramer:

Has the tetramer been observed?, J. Chem. Phys. **137**, 214316 (2012).

[105] M. Tobita, S. A. Perera, M. Musial, R. J. Bartlett, M. Nooijen, and J. S. Lee, Critical comparison of single-reference and multireference coupled-cluster methods: Geometry, harmonic frequencies, and excitation energies of N_2O_2, J. Chem. Phys. **119**, 10713 (2003).

[106] J. P. Perdew, K. Burke, and M. Ernzerhof, Generalized gradient approximation made simple, Phys. Rev. Lett. **77**, 3865 (1996).

[107] K. Lee, É. D. Murray, L. Kong, B. I. Lundqvist, and D. C. Langreth, Higher-accuracy van der Waals density functional, Phys. Rev. B **82**, 081101 (2010).

[108] I. Hamada, Van der Waals density functional made accurate, Phys. Rev. B **89**, 121103 (2014).

[109] C. Adamo and V. Barone, Toward reliable density functional methods without adjustable parameters: The PBE0 model, J. Chem. Phys. **110**, 6158 (1999).

[110] A. D. Becke, Density-functional thermochemistry. III. The role of exact exchange, J. Chem. Phys. **98**, 5648 (1993).

[111] S. Grimme, Semiempirical GGA-type density functional constructed with a long-range dispersion correction, J. Comp. Chem. **27**, 1787 (2006).

[112] T. Bučko, S. Lebègue, J. G. Ángyán, and J. Hafner, Extending the applicability of the Tkatchenko-Scheffler dispersion correction via iterative Hirshfeld partitioning, J. Chem. Phys. **141**, 034114 (2014).

[113] J. Ning, M. Kothakonda, J. W. Furness, A. D. Kaplan, S. Ehlert, J. G. Brandenburg, J. P. Perdew, and J. Sun, Workhorse minimally empirical dispersion-corrected density functional with tests for weakly bound systems: r^2SCAN+rVV10, Phys. Rev. B **106**, 075422 (2022).

[114] J. Heyd, G. E. Scuseria, and M. Ernzerhof, Hybrid functionals based on a screened Coulomb potential, J. Chem. Phys. **118**, 8207 (2003).

[115] J. P. Singh, W. C. Lim, S. O. Won, J. Song, and K. H. Chae, Synthesis and characterization of some alkaline-earth-oxide nanoparticles, J. Korean Phys. Soc. **72**, 890 (2018).

[116] J. K. Burdett, T. Hughbanks, G. J. Miller, J. W. Richardson, and J. V. Smith, Structural-electronic relationships in inorganic solids: Powder neutron diffraction studies of the rutile and anatase polymorphs of titanium dioxide at 15 and 295 K, J. Am. Chem. Soc. **109**, 3639 (1987).

[117] G. Kresse and J. Furthmüller, Efficiency of ab-initio total energy calculations for metals and semiconductors using a plane-wave basis set, Comput. Mater. Sci. **6**, 15 (1996).

[118] G. Kresse and J. Furthmüller, Efficient iterative schemes for ab initio total-energy calculations using a plane-wave basis set, Phys. Rev. B **54**, 11169 (1996).

[119] S. Grimme, Supramolecular binding thermodynamics by dispersion-corrected density functional theory, Chem. - Eur. J. **18**, 9955 (2012).

[120] Y.-P. Li, J. Gomes, S. Mallikarjun Sharada, A. T. Bell, and M. Head-Gordon, Improved force-field parameters for QM/MM simulations of the energies of adsorption for molecules in zeolites and a free rotor correction to the rigid rotor harmonic oscillator model for adsorption enthalpies, J. Phys. Chem. C **119**, 1840 (2015).

[121] D. Alfè and M. J. Gillan, Ab initio statistical mechanics of surface adsorption and desorption. II. Nuclear quantum effects, J. Chem. Phys. **133**, 044103 (2010).

[122] G. Piccini and J. Sauer, Effect of anharmonicity on adsorption thermodynamics, J. Chem. Theory Comput. **10**, 2479 (2014).

[123] A. S. Rosen, Quacc – The Quantum Accelerator: https://zenodo.org/records/13921187, Zenodo (2024).

[124] C. T. Campbell, Energies of adsorbed catalytic intermediates on transition metal surfaces: Calorimetric measurements and benchmarks for theory, Acc. Chem. Res. **52**, 984 (2019).

[125] M. Schmid, G. S. Parkinson, and U. Diebold, Analysis of temperature-programmed desorption via equilibrium thermodynamics, ACS Phys. Chem Au **3**, 44 (2023).

[126] Z. Dohnálek, G. A. Kimmel, S. A. Joyce, P. Ayotte, R. S. Smith, and B. D. Kay, Physisorption of CO on the MgO(100) surface, J. Phys. Chem. B **105**, 3747 (2001).

[127] P. A. Redhead, Thermal desorption of gases, Vacuum **12**, 203 (1962).

[128] C. T. Campbell and J. R. V. Sellers, The entropies of adsorbed molecules, J. Am. Chem. Soc. **134**, 18109 (2012).

[129] A. D. Boese and J. Sauer, Accurate adsorption energies of small molecules on oxide surfaces: CO–MgO(001), Phys. Chem. Chem. Phys. **15**, 16481 (2013).

[130] M. Alessio, D. Usvyat, and J. Sauer, Chemically accurate adsorption energies: CO and H_2O on the MgO(001) surface, J. Chem. Theory Comput. **15**, 1329 (2019).

[131] J. H. Espenson, Chemical kinetics and reaction mechanisms (McGraw-Hill, 1995).

[132] J. Wellendorff, T. L. Silbaugh, D. Garcia-Pintos, J. K. Nørskov, T. Bligaard, F. Studt, and C. T. Campbell, A benchmark database for adsorption bond energies to transition metal surfaces and comparison to selected DFT functionals, Surf. Sci. **640**, 36 (2015).

[133] S. Rangarajan and M. Mavrikakis, A comparative analysis of different van der Waals treatments for molecular adsorption on the basal plane of 2H-MoS_2, Surf. Sci. **729**, 122226 (2023).

[134] W. A. Brown, R. Kose, and D. A. King, Femtomole adsorption calorimetry on single-crystal

surfaces, Chem. Rev. **98**, 797 (1998).

[135] S. L. Tait, Z. Dohnálek, C. T. Campbell, and B. D. Kay, N-alkanes on MgO(100). I. Coverage-dependent desorption kinetics of n-butane, J. Chem. Phys. **122**, 164707 (2005).

[136] S. L. Tait, Z. Dohnálek, C. T. Campbell, and B. D. Kay, N-alkanes on MgO(100). II. Chain length dependence of kinetic desorption parameters for small n-alkanes, J. Chem. Phys. **122**, 164708 (2005).

[137] J. C. Lian, E. Kieseritzky, A. Gonchar, M. Sterrer, J. Rocker, H.-J. Gao, and T. Risse, N_2O adsorption on the surface of MgO(001) thin films: An infrared and TPD study, J. Phys. Chem. C **114**, 3148 (2010).

[138] S. C. Street, Q. Guo, C. Xu, and D. W. Goodman, Adsorption and electronic states of benzene on ordered MgO and Al_2O_3 thin films, J. Phys. Chem. **100**, 17599 (1996).

[139] D. A. Arthur, D. L. Meixner, M. Boudart, and S. M. George, Adsorption, desorption, and surface diffusion kinetics of NH_3 on MgO(100), J. Chem. Phys. **95**, 8521 (1991).

[140] M. J. Stirniman, C. Huang, R. Scott Smith, S. A. Joyce, and B. D. Kay, The adsorption and desorption of water on single crystal MgO(100): The role of surface defects, J. Chem. Phys. **105**, 1295 (1996).

[141] L. Chen, R. S. Smith, B. D. Kay, and Z. Dohnálek, Adsorption of small hydrocarbons on rutile TiO_2(110), Surf. Sci. **650**, 83 (2016).

[142] T. L. Thompson, O. Diwald, and J. T. Yates, CO_2 as a probe for monitoring the surface defects on TiO_2(110) temperature-programmed desorption, J. Phys. Chem. B **107**, 11700 (2003).

[143] M. B. Hugenschmidt, L. Gamble, and C. T. Campbell, The interaction of H_2O with a TiO_2(110) surface, Surf. Sci. **302**, 329 (1994).

[144] Z. Dohnálek, J. Kim, O. Bondarchuk, J. M. White, and B. D. Kay, Physisorption of N_2, O_2, and CO on fully oxidized TiO_2(110), J. Phys. Chem. B **110**, 6229 (2006).

[145] Z. Li, R. S. Smith, B. D. Kay, and Z. Dohnálek, Determination of absolute coverages for small aliphatic alcohols on TiO_2(110), J. Phys. Chem. C **115**, 22534 (2011).

[146] G. S. Herman, Z. Dohnálek, N. Ruzycki, and U. Diebold, Experimental Investigation of the Interaction of Water and Methanol with Anatase-TiO_2(101), J. Phys. Chem. B **107**, 2788 (2003).

[147] S. Koust, K. C. Adamsen, E. L. Kolsbjerg, Z. Li, B. Hammer, S. Wendt, and J. V. Lauritsen, NH_3 adsorption on anatase-TiO_2(101), J. Chem. Phys. **148**, 124704 (2018).

[148] T. Trevethan and A. L. Shluger, Building blocks for molecular devices: Organic molecules on the MgO (001) surface, J. Phys. Chem. C **111**, 15375 (2007).

[149] A. M. Ferrari, S. Huber, H. Knözinger, K. M. Neyman, and N. Rösch, FTIR spectroscopic and density functional model cluster studies of methane adsorption on MgO, J. Phys. Chem. B **102**, 4548 (1998).

[150] A. D. Boese and J. Sauer, Accurate adsorption energies for small molecules on oxide surfaces: CH_4/MgO(001) and C_2H_6/MgO(001), J. Comp. Chem. **37**, 2374 (2016).

[151] R. Valero, J. R. B. Gomes, D. G. Truhlar, and F. Illas, Good performance of the M06 family of hybrid meta generalized gradient approximation density functionals on a difficult case: CO adsorption on MgO(001), J. Chem. Phys. **129**, 124710 (2008).

[152] F. R. Rehak, G. Piccini, M. Alessio, and J. Sauer, Including dispersion in density functional theory for adsorption on flat oxide surfaces, in metal–organic frameworks and in acidic zeolites, Phys. Chem. Chem. Phys. **22**, 7577 (2020).

[153] H.-Z. Ye and T. C. Berkelbach, Adsorption and vibrational spectroscopy of CO on the surface of MgO from periodic local coupled-cluster theory, Faraday Discuss. **254**, 628 (2024).

[154] A. Mitra, M. R. Hermes, M. Cho, V. Agarawal, and L. Gagliardi, Periodic density matrix embedding for CO adsorption on the MgO(001) surface, J. Phys. Chem. Lett. **13**, 7483 (2022).

[155] L. Giordano, J. Goniakowski, and J. Suzanne, Partial dissociation of water molecules in the (3x2) water monolayer deposited on the MgO(100) surface, Phys. Rev. Lett. **81**, 1271 (1998).

[156] J. Carrasco, F. Illas, and N. Lopez, Dynamic ion pairs in the adsorption of isolated water molecules on alkaline-earth oxide (001) surfaces, Phys. Rev. Lett. **100**, 016101 (2008).

[157] X. L. Hu, J. Carrasco, J. Klimeš, and A. Michaelides, Trends in water monomer adsorption and dissociation on flat insulating surfaces, Phys. Chem. Chem. Phys. **13**, 12447 (2011).

[158] G. G. Kebede, D. Spångberg, P. D. Mitev, P. Broqvist, and K. Hermansson, Comparing van der Waals DFT methods for water on NaCl(001) and MgO(001), J. Chem. Phys. **146**, 064703 (2017).

[159] O. Karalti, D. Alfè, M. J. Gillan, and K. D. Jordan, Adsorption of a water molecule on the MgO(100) surface as described by cluster and slab models, Phys. Chem. Chem. Phys. **14**, 7846 (2012).

[160] S. Pugh and M. J. Gillan, The energetics of NH_3 adsorption at the MgO(001) surface, Surf. Sci. **320**, 331 (1994).

[161] Y. Nakajima and D. J. Doren, Ammonia adsorption on MgO(100): A density functional theory study, J. Chem. Phys. **105**, 7753 (1996).

[162] A. Allouche, F. Corà, and C. Girardet, Vibrational infrared spectrum of NH_3 adsorbed on

MgO(100). I. Ab initio calculations, Chem. Phys. **201**, 59 (1995).

[163] G. Lv, S. Li, H. Zhang, W. Qian, J. Cheng, and P. Qian, CO_2 adsorption on a K-promoted MgO surface: A DFT theoretical study, Surf. Sci. **749**, 122575 (2024).

[164] Z. Song, B. Zhao, H. Xu, and P. Cheng, Remarkably strong chemisorption of nitric oxide on insulating oxide films promoted by hybrid structure, J. Phys. Chem. C **121**, 21482 (2017).

[165] Y. Yanagisawa, K. Kuramoto, and S.-i. Yamabe, Three types of adsorptions of nitric oxide on the MgO surface, J. Phys. Chem. B **103**, 11078 (1999).

[166] J. A. Rodriguez, T. Jirsak, M. Pérez, L. González, and A. Maiti, Studies on the behavior of mixed-metal oxides: Adsorption of CO and NO on MgO(100), $Ni_xMg_{1-x}O(100)$, and $Cr_xMg_{1-x}O(100)$, J. Chem. Phys. **114**, 4186 (2001).

[167] R. Añez, A. Sierraalta, and L. J. D. Soto, NO and NO_2 adsorption on subsurface doped MgO (100) and BaO (100) surfaces. A density functional study, Appl. Surf. Sci. **404**, 216 (2017).

[168] M. Miletic, J. L. Gland, K. C. Hass, and W. F. Schneider, First-principles characterization of NO_x adsorption on MgO, J. Phys. Chem. B **107**, 157 (2003).

[169] W. F. Schneider, K. C. Hass, M. Miletic, and J. L. Gland, Dramatic cooperative effects in adsorption of NO_x on mgo(001), J. Phys. Chem. B **106**, 7405 (2002).

[170] W. F. Schneider, Qualitative differences in the adsorption chemistry of acidic (CO_2, SO_x) and amphiphilic (NO_x) species on the alkaline earth oxides, J. Phys. Chem. B **108**, 273 (2004).

[171] X. Lu, X. Xu, N. Wang, and Q. Zhang, Adsorption and decomposition of NO on magnesium oxide: A quantum chemical study, J. Phys. Chem. B **103**, 5657 (1999).

[172] J. Y. Lim, K. Kim, E. Y. Kim, and J. W. Han, Density functional theory study of NO_x adsorption on alkaline earth metal oxide and transition metal surfaces, Korean J. Chem. Eng. **36**, 1258 (2019).

[173] A. H. Rodríguez, M. M. Branda, and N. J. Castellani, Adsorption of n-methanol molecules on MgO(100) with $n = 1$ to 4: A theoretical study, J. Phys. Chem. C **111**, 10603 (2007).

[174] I. D. Gay and N. M. Harrison, A density functional study of water and methanol chemisorption on MgO(110), Surf. Sci. **591**, 13 (2005).

[175] I.-C. Man, S. G. Soriga, and V. Parvulescu, Theoretical aspects of methyl acetate and methanol activation on MgO(100) and (501) catalyst surfaces with application in FAME production, Appl. Surf. Sci. **392**, 920 (2017).

[176] X. L. Hu, J. Klimeš, and A. Michaelides, Proton transfer in adsorbed water dimers, Phys. Chem. Chem. Phys. **12**, 3953 (2010).

[177] R. S. Alvim, I. J. Borges, D. G. Costa, and A. A. Leitão, Density-functional theory simulation of the dissociative chemisorption of water molecules on the MgO(001) surface, J. Phys. Chem. C **116**, 738 (2012).

[178] Z. Ding and A. Selloni, Hydration structure of flat and stepped MgO surfaces, J. Chem. Phys. **154**, 114708 (2021).

[179] P. J. Stimac and R. J. Hinde, Simulating CH_4 physisorption on ionic crystals, Eur. Phys. J. D **46**, 69 (2008).

[180] C. Pisani, L. Maschio, S. Casassa, M. Halo, M. Schütz, and D. Usvyat, Periodic local MP2 method for the study of electronic correlation in crystals: Theory and preliminary applications, J. Comp. Chem. **29**, 2113 (2008).

[181] M. J. Tillotson, P. M. Brett, R. A. Bennett, and R. Grau-Crespo, Adsorption of organic molecules at the TiO_2(110) surface: The effect of van der Waals interactions, Surf. Sci. **632**, 142 (2015).

[182] Ž. Kovačič, B. Likozar, and M. Huš, Electronic properties of rutile and anatase TiO_2 and their effect on CO_2 adsorption: A comparison of first principle approaches, Fuel **328**, 125322 (2022).

[183] L.-M. Liu, C. Zhang, G. Thornton, and A. Michaelides, Structure and dynamics of liquid water on rutile TiO_2(110), Phys. Rev. B **82**, 161415 (2010).

[184] A. V. Bandura, D. G. Sykes, V. Shapovalov, T. N. Troung, J. D. Kubicki, and R. A. Evarestov, Adsorption of water on the TiO_2 (rutile) (110) surface: A comparison of periodic and embedded cluster calculations, J. Phys. Chem. B **108**, 7844 (2004).

[185] E. V. Stefanovich and T. N. Truong, Ab initio study of water adsorption on TiO_2(110): Molecular adsorption versus dissociative chemisorption, Chem. Phys. Lett. **299**, 623 (1999).

[186] H.-Z. Ye and T. C. Berkelbach, Ab initio surface chemistry with chemical accuracy, Preprint at https://arxiv.org/abs/2309.14640 (2024).

[187] T. Schäfer, F. Libisch, G. Kresse, and A. Grüneis, Local embedding of coupled cluster theory into the random phase approximation using plane waves, J. Chem. Phys. **154**, 011101 (2021).

[188] L. Kieu, P. Boyd, and H. Idriss, Trends within the adsorption energy of alcohols over rutile TiO_2(110) and (011) clusters, J. Mol. Catal. A: Chem. **188**, 153 (2002).

[189] S. P. Bates, G. Kresse, and M. J. Gillan, The adsorption and dissociation of ROH molecules on TiO_2(110), Surf. Sci. **409**, 336 (1998).

[190] X. Lang, B. Wen, C. Zhou, Z. Ren, and L.-M. Liu, First-principles study of methanol oxidation into methyl formate on rutile TiO_2(110), J. Phys. Chem. C **118**, 19859 (2014).

[191] A. Vittadini, A. Selloni, F. P. Rotzinger, and M. Grätzel, Structure and energetics of water

adsorbed at tio$_2$ anatase (101) and (001) surfaces, Phys. Rev. Lett. **81**, 2954 (1998).

[192] K. L. Miller, C. B. Musgrave, J. L. Falconer, and J. W. Medlin, Effects of water and formic acid adsorption on the electronic structure of anatase TiO_2(101), J. Phys. Chem. C **115**, 2738 (2011).

[193] I. Onal, S. Soyer, and S. Senkan, Adsorption of water and ammonia on TiO_2-anatase cluster models, Surf. Sci. **600**, 2457 (2006).

[194] Z. Zhao, Z. Li, and Z. Zou, Structure and properties of water on the anatase TiO_2(101) surface: From single-molecule adsorption to interface formation, J. Phys. Chem. C **116**, 11054 (2012).

[195] T. Petersen and T. Klüner, Water adsorption on ideal anatase-TiO_2(101) – An embedded cluster model for accurate adsorption energetics and excited state properties, Z. Phys. Chem. **234**, 813 (2020).

[196] R. Wanbayor and V. Ruangpornvisuti, Adsorption of CO, H_2, N_2O, NH_3 and CH_4 on the anatase TiO_2(001) and (101) surfaces and their competitive adsorption predicted by periodic DFT calculations, Mater. Chem. Phys. **124**, 720 (2010).

[197] J.-G. Chang, S.-P. Ju, C.-S. Chang, and H.-T. Chen, Adsorption configuration and dissociative reaction of NH_3 on anatase(101) surface with and without hydroxyl groups, J. Phys. Chem. C **113**, 6663 (2009).

[198] L. Lin, Adaptively compressed exchange operator, J. Chem. Theory Comput. **12**, 2242 (2016).
